# Genomic Structure and Variation of Nuclear Factor (Erythroid-Derived 2)-Like 2

**DOI:** 10.1155/2013/286524

**Published:** 2013-07-10

**Authors:** Hye-Youn Cho

**Affiliations:** Laboratory of Respiratory Biology, National Institute of Environmental Health Sciences, National Institutes of Health, 111 TW Alexander Dr., Building 101, MD D-201, Research Triangle Park, NC 27709, USA

## Abstract

High-density mapping of mammalian genomes has enabled a wide range of genetic investigations including the mapping of polygenic traits, determination of quantitative trait loci, and phylogenetic comparison. Genome sequencing analysis of inbred mouse strains has identified high-density single nucleotide polymorphisms (SNPs) for investigation of complex traits, which has become a useful tool for biomedical research of human disease to alleviate ethical and practical problems of experimentation in humans. Nuclear factor (erythroid-derived 2)-like 2 (*NRF2*) encodes a key host defense transcription factor. This review describes genetic characteristics of human *NRF2* and its homologs in other vertebrate species. *NRF2* is evolutionally conserved and shares sequence homology among species. Compilation of publically available SNPs and other genetic mutations shows that human *NRF2* is highly polymorphic with a mutagenic frequency of 1 per every 72 bp. Functional at-risk alleles and haplotypes have been demonstrated in various human disorders. In addition, other pathogenic alterations including somatic mutations and misregulated epigenetic processes in *NRF2* have led to oncogenic cell survival. Comprehensive information from the current review addresses association of *NRF2* variation and disease phenotypes and supports the new insights into therapeutic strategies.

## 1. Overview

**Figure 1:**
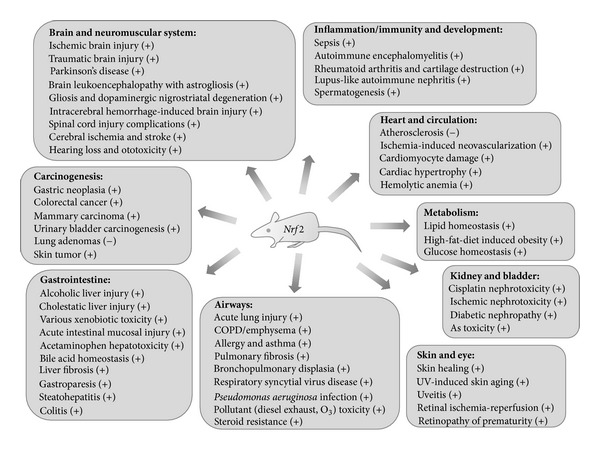
Role of Nrf2 in human pathogenesis learned from model studies using mice genetically deficient in *Nrf2*. (+) indicates protective or beneficial effects and (−) indicates aberrant roles.

The gene nuclear factor (erythroid-derived 2)-like 2 (*NFE2L2*) or more commonly the used synonym nuclear factor erythroid 2- (NF-E2-) related factor 2 (*NRF2*) and its mouse homolog (*Nfe2l2*, *Nrf2*) encode a ubiquitous transcription factor belonging to the basic leucine zipper (bZIP) protein family [1, 2]. NRF2 modulates downstream genes by binding to their *cis*-regulatory module antioxidant response elements (AREs). NRF2 targets include ARE-bearing effector genes such as reactive oxygen species (ROS) scavenging enzymes (e.g., superoxide dismutases, SODs), phase-2 defense enzymes (e.g., glutathione-S-transferase, GST; heme oxygenase-1, HO-1), drug efflux pumps (e.g., multidrug resistance proteins, MRPs), and various interacting and indirectly modulated proteins [3–6]. The NRF2-ARE pathway has emerged in mechanisms of human diseases in which oxidative stress is implicated. Importantly, three lines of gene-targeted (knockout) mice were generated by Drs. M. Yamamoto (*Nfe2l2* ^tm1Mym^), Y. W. Kan (*Nfe2l2* ^tm1Ywk^), and P. A. Ney (*Nfe2l2* ^tm1Ney^) [7–9], and 124 gene-trapped or gene-targeted cell lines have been established (http://www.informatics.jax.org/searches/allele_report.cgi?markerID=MGI:108420). During the last decade or more, wide application of the knockout mice to human disease models has led to new insights into disease pathogenesis and therapeutic potential (Figure 1). 

Kelch-like ECH-activating protein 1 (KEAP1 for humans, Keap1 for mice, or iNrf2 for rats) is a cytoplasmic suppressor of NRF2 and is critical in NRF2 homeostasis and activity [10]. Substantial efforts have led to the discovery of the molecular mechanisms of KEAP1-mediated NRF2 regulation. In unstressed conditions, the NRF2-bound KEAP1 homodimer is complexed to a ubiquitin ligase (Cullin 3-based E3 ligase), which polyubiquitinates NRF2 for proteasomal degradation and maintains NRF2 homeostasis (20 min half-life of cellular NRF2 [11]). However, modifications of KEAP1 (e.g., cysteine residues) and NRF2 (e.g., serine residues) under stressed conditions activate NRF2 by liberating it from a “hinge and latch” NRF2-KEAP1 affinity binding, allowing its nuclear translocation [12]. 

In the current review, I address genetic aspects of human *NRF2* and its homologs in other vertebrate species. Sequence variations in human *NRF2* and murine *Nrf2* including single-nucleotide polymorphisms (SNPs) were collected from public databases and compiled. Mutations that have been associated with disease risks are defined. Nongenetic variations including somatic mutations and epigenetic modifications are also described. Although the current review does not deal with mutations in other species, recent characterization of* nrf2* mutant zebrafish which were hypersensitive to environmental toxicants [13] also provides a useful investigational tool. 

## 2. Sequence of NF-E2-Related Factor 2 and Cross-Species Homology 

Homology scores of gene (coding DNA sequences, cds) and protein across 10 species were compared with human *NRF2*. The highest sequence homology (98%-99%) was with chimpanzee and rhesus monkey while the lowest similarity was found with zebrafish (Table 1). While there is approximately about 83% homology in cds and protein sequences of humans and rodents, 5′-untranslated regions (5′-UTR, UTR-5) of these strains extend differentially (114 bp in human, 233 bp in mouse, and 82 bp in rat), and the human 5′-UTR does not share significant sequence homology with either rat or mouse (the rat is 94% homologous with the 3′ portion of mouse 5′-UTR). 

Human *NRF2* is located in the cytogenetic band 2q31.2 of chromosome 2 spanning 178,095,031–178,129,859 bp as a complementary sequence (gene ID: 4780, Table 1). Murine *Nrf2* maps as a complementary sequence to chromosome 2 C3 (44.75 centimorgan) and spans 75,675,519–75,704,641 (gene ID: 18024, Table 1). The complete cds of *NRF2* is 2,859 bp, and there are 14 transcript variants reported (http://useast.ensembl.org/Homo_sapiens). Mouse Nrf2 mRNA spans 2,469 bp, and another variant has been reported (http://useast.ensembl.org/Mus_musculus). The human NRF2 protein (ID: NP_006155) contains 605 amino acid (aa) residues with molecular weight of 67.7 kDa (isoform 1), and total 12 isoforms are published (the National Center for Biotechnology Information, NCBI, http://www.ncbi.nlm.nih.gov/refseq/rsg/; e!Ensemble, http://useast.ensembl.org/index.html; UniProt Consortium, http://www.uniprot.org/uniprot/Q16236, http://www.uniprot.org/uniprot/Q60795). Mouse Nrf2 protein (ID: NP_035032) comprises 597 aa at 66.8 kDa (Table 1). Structurally, there are 6 NRF2-ECH homology (Neh) domains configuring the protein sequence of either species (Table 2), and the potential functions of each region, particularly the highly conserved KEAP1-binding Neh2 and DNA- (ARE-) binding Neh1 domains, have been intensively investigated [12, 14, 15]. 

## 3. Genetic Variation of NF-E2-Related Factor 2 in Human and Mouse

### 3.1. Evolution, Genome Sequence, and Polymorphism Discovery in Human and Mouse

While rare and monogenic Mendelian diseases are inheritable mutations in a single gene [16], many common diseases are complex traits, and the disease phenotypes are affected by variants in multiple genetic loci. Recent advancements in high-throughput technology have enabled sequencing of entire mammalian genomes [17–19], and information on DNA sequence and variation has facilitated the study of complex traits of human disorders. Genome-wide association studies (GWAS) examine whether SNPs are associated with important disease traits and ascertains “at-risk” genotypes that are significantly more prevalent in the affected group than in the nonaffected group. The HapMap Project (http://hapmap.ncbi.nlm.nih.gov/) has mapped combinations of alleles at specific loci (haplotypes), that is, common patterns of sequence variation in several human populations. It has supported efficient mapping of multiple loci for complex traits in GWAS. Candidate gene approaches based on findings from GWAS of similar disorders have also been useful for determining the potential genetic mechanisms of diseases. 

The evolutionary divergence of human and mouse lineages occurred for roughly 75 million years, and their genome sequences have been altered by nearly one substitution (or deletion or addition) for every two nucleotides [20]. However, this slow evolution process resulted in a high degree of conservation across the two species, which allows alignment of orthologous sequences: >90% of the human and mouse genomes is partitioned into corresponding regions of conserved synteny, and at the nucleotide level approximately 40% of the human genome is aligned to the mouse [20]. Due to this fact, biomedical studies of human genes are complemented by experimental manipulation of corresponding mouse genes, and they have aided functional understanding of genes in human health. Following the 2003 completion of the Human Genome Project of approximately 3.1 giga base pairs (Gbp), the Mouse Genome Project assembled the complete genome sequence of one strain (C57BL/6J; 2,716,965,481 bp) in 2011. Using this reference strain, whole genome sequencing data across 16 additional inbred strains were done (http://www.sanger.ac.uk/resources/mouse/genomes/, [21]). Discovery of high-density SNPs in the mouse genome supports evolutionary history of the strain and provides a tool to investigate models of human disease processes that cannot often be practically achieved through direct human studies. 

### 3.2. Genetic Mutations in Human *NRF2*

**Figure 2:**
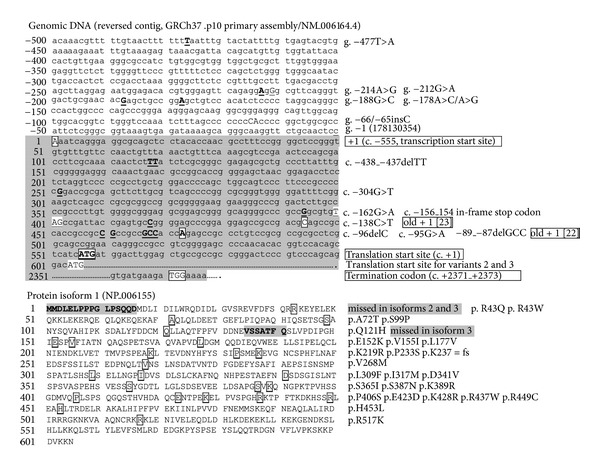
DNA (variant 1 partial promoter and exons 1 and 5) and protein (isoform 1) sequence of Human *NRF2*. SNPs and amino acid residues for nonsynonymous SNPs are marked.

Human *NRF2* codes three major isoforms of protein (Figure 2). Transcript variant 2 (NM_001145412.2, 2746 bp) has an alternate promoter, 5′-UTR, and a downstream start codon, compared to variant 1 (NM_006164.4, 2859 bp). It encodes an isoform 2 missing N-terminal 16 aa (NP_001138884 or Q16236-2, 589 aa) relative to isoform 1 (NP_006155.2 or Q16236, 605 aa). Isoform 3 (NP_001138885.1 or Q16236-3, 582 aa) is encoded by transcript variant 3 (NM_001145413.2; 2,725 bp) and lacks an internal segment relative to isoform 2 due to an alternate in-frame splice site in the 3′ coding region. In public databases, more than 583 sequence mutations are reported in *NRF2 *(34,827 bp) and 7,000 bp upstream (Table S1; data acquired as of December, 2012) (See Table S1 in Supplementary Material available on line at http://dx.doi.org/10.1155/2013/286524). *NRF2* locates on 178,130,354-178,129,304 bp of GRCh37.p10 Primary Assembly, and Figure 2 shows sequences of proximal promoter (−1 to −500), partial mRNA variant 1 including 5′-UTR (exon 1, up to TSS), and protein isoform 1 (NP_006155, encoded by variant 1 NM_006164.4: 556–2,373 bp). Based on the current assembly and sequence update, previous promoter positions −686 [22]/−653 [23] are identified as −214; −684 [22]/−651 [23] as −212; and −650 [22]/−617 [23] as −178. Overall frequency of *NRF2* SNPs and other mutations is about 1 per 72 bp. The genetic mutations include 37 in the 5′ flanking promoter and 59 in exons (Tables 3 and S1). Among exon SNPs, 26 are nonsynonymous (Cns) mutations. A triplet repeat variation (rs143406266; GCC_4_  versus GCC_5_, previously published as −20/−6) in the 5′-UTR was uniquely identified in Asian populations [22, 24]. 

### 3.3. SNPs, Haplotypes, and Association with Disease Risk

The use of gene knockout mice in model systems has provided potential insights into the role of *NRF2* in the pathogenesis of various human disorders (see Figure 1). Recent epidemiological and association studies have revealed significant associations of *NRF2* sequence variations with disease risks, which further supports *NRF2* as a susceptibility gene. Most of the phenotype-associated variants are in the promoter region and presumed to be involved in NRF2 gene regulation.  Table 4 summarizes *NRF2* SNP and/or haplotype alleles that have been associated with oxidant-related disease risks. Interestingly, there is no evidence for exon SNPs as at-risk alleles. For convenience and consistency, intronic and 3′ distal SNP alleles are presented as chromosome contig (HGVS) alleles while promoter and exon SNPs are presented as reversed contig alleles throughout the text.

*Pulmonary Diseases. NRF2* SNPs in the promoter and intron 1 sequences have been investigated for their potential associations with risk of pulmonary critical disorders including acute lung injury (ALI), cigarette smoke-induced chronic obstructive pulmonary disease (COPD), and asthma. A heterozygous C/A SNP at −178 position (rs6721961T>C or T>G, previously −617 or −650) significantly increased the risk for developing ALI following major trauma in European and African-American populations (odds ratio, OR 6.44; 95% confidence interval, CI 1.34–30.8; allelic frequency = 11.9% at 21/180) [23]. Promoter activity of the A allele (A/C or A/A) determined *in vivo* and *in vitro* was significantly lower than C/C allele at that locus (−178 in an ARE-like motif) indicating that it is a functional SNP for autoregulation [23]. The −178G/G were also nominally associated with ALI-related 28-day mortality following systemic inflammatory response syndrome [37]. In a Japanese cohort, SNP haplotype (rs2001350T/rs6726395A/rs1962142A/rs2364722A/rs6721961T) containing the −178A/A homozygote was associated with an annual decline of rapid forced expiratory volume in one second (FEV_1_) in relation to cigarette-smoking status [34]. In addition, a promoter and 5′-UTR SNP haplotype consisting of −214 G allele (52%, rs35652124, previously −686/−653), −212G/G (98%, rs67006649, previously −684/−651), −178C allele (73%), and GCC_4_ (53%) was predicted to increase respiratory failure development (hazard ratio = 0.95, CI 0.91–0.99) in German COPD patients [32]. Significant interaction was also identified between an intronic SNP G allele (rs6726395, g.178103229A>G, 88.4% frequency) and smoking status on FEV_1_ decline, relative to the reference A/A allele, in the above Japanese cohort [34]. Siedlinski et al. [39] reported that the C/C genotype of another intronic SNP (rs2364723, g.178126546G>C) was associated with a lower FEV_1_ level compared to the wild-type genotype (G/G) in two Netherland cohorts (CI, −63.6−17.8, frequency = 0.525, and pooled cohort size = 2,542). This SNP alone or as a haplotype with 4 more intronic SNPs (rs13001694G/rs1806649T/rs4243387T/rs6726395G) was also associated with high FEV_1_ levels in individuals that ever smoked [39]. In a Hungarian population of childhood asthma, SNPs at −178 (C/A) and 3′ flanking (rs2588882T/G) loci were inversely associated with infection-induced asthma (OR 0.437; CI 0.28–0.80, OR 0.290; CI 0.13–0.62, resp.), and these SNPs significantly influenced an asthma-environmental pollution interaction [38]. The intronic SNP rs1806649 (C>T) was associated but not significantly with an increased risk of hospitalization during high-level particulate matter (PM_10_) periods in asthma or COPD patients (*n* = 209) of the United Kingdom (UK) [41]. Asthma and COPD admission rates were related to the increase in environmental PM_10_ concentration. Importantly, effects of interaction between prenatal stress and *NRF2* SNPs on descendant pulmonary health were investigated by the Avalon Longitudinal Study in the UK: maternal smoking during pregnancy was not associated with lung function change determined by maximum mild expiratory flow (FEF_25–75_) or with asthma incidence in school-aged children, and this relation was not modified by *NRF2* SNP genotypes [42]. However, early gestation acetaminophen exposure significantly influenced the risk of asthma and wheezing at the age of 7 years in >4,000 mothers and >5,000 children [33]. When maternal copies of the −212A allele were present, association with asthma (1,137/4,891; OR 1.73, CI 1.22–2.45) and wheezing (1,149/4,949; OR 1.53, 95% CI 1.06–2.20) was significantly increased [33]. 

*Gastrointestinal Disorders.* While there was no evidence in lung cancer cases, studies in Japanese populations suggested a potential association of *NRF2 *variations with gastro-intestinal tumorigenesis. *Helicobacter pylori* (*H. pylori*) causes gastritis which can lead to gastric atrophy and cancer. In gastric epithelium from the Japanese cancer cohorts (39 gastric cancers, 46 controls), *H. pylori* infection was positively correlated with aberrant CpG island methylation of tumor suppressor genes (e.g., p14), and −214G/−212G or −214A/−212G *NRF2 *haplotype was significantly associated with increased (OR 2.90; 95% CI 1.14–7.36) or decreased (OR 0.33; 95% CI 0.13–0.88) risk of the CpG methylation, respectively, in the *H. pylori*-infected patients [29]. Further study from the same investigators determined that −214A/−212G allele carriers had significantly (*P* = 0.022) reduced risk of gastric cancer in *H. pylori*-negative cases [30]. The −214A/G−212A/G genotypes were negatively associated (OR 0.45, CI 0.22–0.93), and the −214G−212G genotypes were positively associated (chronic continuous phenotype; OR 2.57, CI 1.01–6.60) with ulcerative colitis (89 patients, 141 controls) in a Japanese population [31].

*Autoimmune Disorders.* Systemic lupus erythematosus (SLE) is a long-term autoimmune disease more frequently found in females than in males. It affects organs including skin, joints, kidneys, and brain, and nephritis is an aggressive characteristic in some patients. Genome-wide association studies in humans identified a suggestive quantitative trait locus near *NRF2* [43]. A study of a Mexican Mestizo population (362 patients with childhood-onset SLE, 379 controls, and 212 nephritis diagnosed) determined that lupus with nephritis was significantly (OR 1.81, CI 1.04–3.12) associated with the −214G/A SNP in females [25]. The same SNPs were not closely associated with SLE risk in a Japanese cohort [22]. Vitiligo is a skin condition in which there is a loss of brown color (pigment) from areas of skin, resulting in irregular white patches. It is thought to be an autoimmune disease caused by loss of cells (melanocytes) that produce brown pigment. A study indicated that the −178A allele increased the risk of vitiligo dose-dependently (OR 1.724, 95% CI 1.35–2.21 for C/A; OR 2.902, CI 1.62–5.19 for A/A) [35].

*Female Disorders.* It is well known that estrogen metabolites (e.g., catechols) cause ROS formation suggesting correlation of NRF2 and downstream effectors in postmenopausal mammary cancer. In a study of a Finish population (Kuopio Breast Cancer Project, *n* = 452 patients, 370 controls), the −178A/A homozygous genotype (OR 4.656; CI = 1.35–16.06) and 3′ flanking rs2706110 (T/T; OR 2.079, CI 1.18–3.68) genotype were associated with increased risk of breast cancer, while the 5′ flanking −3,306T/T homozygous allele was significantly associated with lower survival (frequency = 71/219, OR 1.687, CI 1.105–2.75) [27], suggesting that *NRF2 *genetic polymorphisms affect susceptibility and outcome of the patients. The −178A allele carriers together with intronic rs1962142A allele carriers were associated with lowered tissue levels of NRF2 proteins [27]. In postmenopausal women, the −178A allele (OR 17.9; 95% CI 3.70–85.70) appeared to modify the risk of venous thromboembolism caused by oral estrogen therapy (A/A or A/C frequency = 33.3%) as demonstrated by the French ESTHER study (161 cases, 474 controls) [36]. An intronic rs1806649C>T SNP did not associate with breast cancer risk in postmenopausal women [40]. However, when this SNP and other at-risk alleles of ARE-responsive genes (*NQO1*, *HO-1*, *NOS3*) were combined, there was a significant gene-dose effect on the breast cancer risk [40]. Although coding region SNPs in *NRF2* and *KEAP1* were identified in the Japanese endometrial adenocarcinoma patients, no association of *NRF2* SNPs with the disease was found [44]. 

*Neurodegenerative Diseases.*  Oxidative stress is known to be involved in Parkinson's disease (PD) presumably due to production of ROS from high-dopamine metabolism and low levels of antioxidants in the substantia nigra of the brain. Investigators found a protective *NRF2* haplotype consisting of four 5′ flanking SNPs (−5238G/−214A/−212G/−178C) and 4 intronic SNPs (rs2886161A/rs1806649A/rs2001350A/rs10183914A) from Swedish (OR 0.9, CI 0.60–1.40) and Polish (OR 0.4, CI 0.30–0.60) populations (total 165 + 192 PD cases, 190 + 192 controls) [26]. The investigators also suggested that *NRF2 *haplotype alleles were associated with 2 years earlier age of Alzheimer's disease (AD) onset, 4 years earlier age of posterior subcapsular cataract surgery, and 4 years later age of cortical cataract surgery while they were not significantly related to AD or age-related cataract risk [45].

### 3.4. Genetic Mutations in Mouse *Nrf2*

Tsang et al. [46] compiled 673 SNPs in 55 mouse strains and constructed their phylogenetic tree to correlate and clarify the origins of strains based on the assembled mouse genome sequence and SNP data [20, 47, 48]. Recently, using the complete genome sequence of C57BL/6J (B6) mouse as a reference, high-density SNP screening in other laboratory strains or in panels of strains has been published (see [17]). Although millions of mouse SNPs (>10,089,892 as of December 2012) and haplotype mappings from more than 120 strains have been published as valuable references for dissecting the genetic basis of complex traits [49–51], little attention has been paid to polymorphisms of *Nrf2* and their correlation with disease phenotypes.

**Figure 3:**
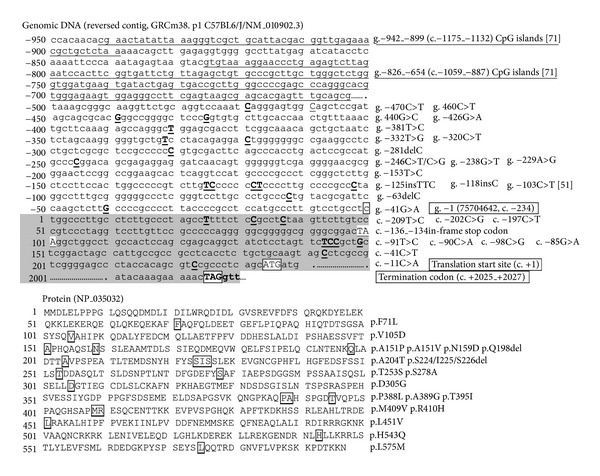
DNA (partial promoter and exons 1 and 5) and protein sequence of mouse Nrf2. SNPs and amino acid residues for non-synonymous SNPs are marked. Promoter regions bearing 5 CpG islands are underlined.

Figure 3 demonstrates the proximal promoter region (−1 to −950)/5′-UTR (exon 1, up to TSS) and protein sequence of mouse *Nrf2* based on GRCm38.p1 Primary Assembly (75,704,641–75,675,513 bp), mRNA variant 1, and protein (NP_035032, encoded by NM_010902: 234–2,027 bp) sequences. Genetic variations in the *Nrf2* genome of inbred strains collected from public databases are listed in Tables S2 and 5. (See Supplementary Table S2 in Supplementary Material available online at http://dx.doi.org/10.1155/2013/286524.) Overall, 968 genetic mutations are compiled for *Nrf2 *gene and 5 kb upstream/2 kb downstream regions: 785 SNPs between B6 and another 16 strains were acquired from the Mouse Phenome Database (MPD, http://phenome.jax.org/db/q?rtn=snp/ret1), and additional SNPs and other mutations were acquired from NCBI dbSNP (http://www.ncbi.nlm.nih.gov/snp/?term=mus+musculus%20nfe2l2). In total, 132 mutations are in the promoter (37 in proximal 1 kb), 49 in exons (38 in coding region, 19 Cns), 727 in introns, and 60 in the 3′ flanking region. Excluding mutations in the 5′ and 3′ flanking sequences, murine *Nrf2 *sequences appear to be more highly variable (1 variation per 37.5 bp) than much of the mouse genome which has an approximate frequency of one SNP per every 245 bp (http://www.informatics.jax.org/mgihome/homepages/stats/all_stats.shtml#allstats_snp). 

*Nrf2* was found to be a susceptibility gene from genome-wide linkage analysis in a murine model of hyperoxia-induced ALI [52]. A promoter SNP −103T>C (previously published as −336T>C) in *Nrf2* was found and predicted to add an additional Sp1 binding site in hyperoxia-susceptible B6 mice, but not in resistant C3H/HeJ mice [52]. Genotypes from the SNP and from simple-sequence length polymorphism markers of the *Nrf2* locus (D2Mit248 and D2Mit94) cosegregated in the B6C3F_2_ mouse cohort [52], and *Nrf2* deficient mice were significantly more susceptible to ALI sub-phenotypes caused by hyperoxia than similarly exposed wild-type mice, supporting *Nrf2* as a contributor to the phenotypic traits [53]. Although no other functional analyses on *Nrf2* SNPs or haplotype association studies have been conducted in inbred mice, strains bearing haplotypes such as multiple Cns in functional domains (e.g., F71L, L451V, H543Q, and L575M) may be useful to elucidate the role of Nrf2 in differential susceptibility to oxidative diseases. 

## 4. Oncogenic Somatic Mutations in Human NF-E2-Related Factor 2 

Somatic mutation is a change in the DNA of somatic cells that affects derived cells but is not inherited by offspring. Efforts to discover somatic mutations have provided insight into mutagenesis and cancer development. Lung cancer, particularly non-small cell lung cancer (NSCLC), is the leading cause of cancer death worldwide. Somatic mutations of *NRF2* and *KEAP1* discovered in lung cancer patients have determined the oncogenic potential of *NRF2* [54, 55]. *KEAP1* somatic mutations were associated with its reduced protein levels in lung cancer tissues and cells [56, 57]. Investigations of NSCLC in various ethnic populations as well as cancers in gastrointestine, breast, and prostate have coordinately demonstrated that multiple Cns somatic mutations in *KEAP1* cause dysfunction of the translated protein and in turn constitutive activation of NRF2, increasing risk of neoplasia and chemoresistance [12, 55, 58, 59]. Somatic mutations of *NRF2* have been detected in various cancer tissues (largely squamous cell carcinomas) in Asian populations (Table 6). *NRF2* mutations were significantly associated with NSCLC cases (squamous cell lung carcinoma, adenocarcinoma) of the Japanese (10.7%, [54]), the Chinese (23%, [60]), and the Koreans (8%, [61]) as well as with lung cancer cell lines. Smoking history was also correlated with mutation occurrence in all of the studies [54, 60, 61]. In addition to lung cancers, laryngeal squamous carcinoma (13% in [61]), esophageal squamous cancer (ESC, 22% in [60], 11.4% in [61]), head and neck cancers (25% in [54]), skin (1/17 case in [61]), and oral cancer cell lines had somatic changes in *NRF2*. In contrast to wide-spread *KEAP1* mutations, mutations in *NRF2* were clustered in DLG/ETGE motifs of the Neh2 domain, which are critical in the “hinge and latch” model of KEAP1 binding [12]. Similar to *KEAP1* somatic mutations, it has been postulated that *NRF2* mutations in cancer cells lead to NRF2 accumulation by suppressing its ubiquitination or KEAP1 binding, which eventually confers malignant potential and resistance to chemotherapy.

**Figure 4:**
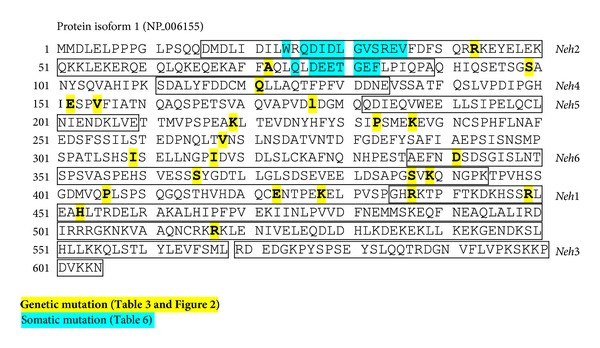
Genetic and somatic mutation loci in human NRF2 protein.

Most variable sites in NRF2 included aa residues 29 (Asp, D), 31 (Gly, G), 77 (Asp, D), and 79 (Glu, E) (Table 6). Residue 33 (Ser, S) in the Neh2 domain is mutated by either genetic or somatic processes (Figure 4). Cns in the EDGF motif of *NRF2* was experimentally determined to impair recognition of KEAP1 [54]. *NRF2* mutations were significantly correlated with increased (2.5-fold) copy number (31% of mutants versus 3% wild types) in Japanese NSCLC cases [63]. Aberrant mutation of *NRF2* also led to increased expression of downstream effectors including *RagD* known to be involved in squamous lung cancer cell proliferation [64], suggesting that the mutation is functional and overcomes KEAP1 inhibition. Singh et al. [65] determined *in vitro *that RNAi-mediated depletion of *NRF2* in lung cancer cells enhanced ROS production and susceptibility to cell death by ionizing radiation. These studies support the concept that elevated NRF2 and ARE responsiveness provides cancer cells with proliferative advantage for malignant transformation and undue protection from anti-cancer therapy. Oncogenic epidermal growth factor receptor (EGFR) signaling is recently found to be critical in NRF2-mediated proliferation of NSCLC cells [66]. 

Collectively, “gain of function” mutations in *NRF2* that reduce KEAP1 recognition are suggested to be predictive markers for poor responsiveness to chemotherapy and radiation therapy. Although NRF2-mediated cellular defense processes are essential in the initiation stage, enhanced NRF2-ARE activity in advanced stages of cancer development may create a favorable intracellular environment for tumor cell growth and survival [67, 68]. In this context, NRF2 may be a potential molecular target for the treatment of radio-resistance cancers, especially those that have “loss of function” mutations in *EGFR*, *KRAS,* or *KEAP1* as well as “gain of function” mutations in *NRF2*. 

## 5. Epigenetic Alterations of NF-E2-Related Factor 2 

Epigenetic modifications are alterations of molecules interacting with genes without changes to the primary DNA sequence. They include post-translational modification of histones, DNA methylation events, chromatin conformational changes, and alterations to noncoding regulatory RNAs. Epigenetic alterations are stable and often inheritable but are reversible and may affect expression of the gene. Dysregulation or defects in epigenetic processes, particularly hypermethylation of tumor suppressor gene promoters (e.g., CpG islands) or histone modifications, are thought to be associated with carcinogenesis. Investigators have reported hypermethylation in CpG islands of *KEAP1* which were associated with reduced *KEAP1* expression in human cancers from lung, prostate, colon, and so forth, [69–72]. Similar to somatic mutations, epigenetic changes on *KEAP1* impaired the function of its encoded protein leading to constitutive NRF2 activation. 

Supporting the role of “pathogenic mutations” in *NRF2*, expression of Nrf2 and downstream Nqo1 was suppressed in prostate tumors of mice (transgenic adenocarcinoma of mouse prostate, TRAMP). Among 15 promoter CpG islands located between −942 and −654 (c.−1175_c.−1132 and c.−1059_c.−887, gap in c.−1131_c.−1060; see Figure 3), hypermethylation of the first 5 CpG islands (−942_−899 and c.−1175_c.−1132) was significantly associated with tumorigenesis [73]. Moreover, treatment with inhibitors for DNA methyltransferase and histone deacetylase restored Nrf2 expression in these tumor cells [73]. A dietary phytochemical curcumin known as a DNA hypomethylation agent restored epigenetically silent *Nrf2* expression through CpG demethylation in carcinogen-induced mouse tumor cells [74]. 

The whole genome epigenetic datasets for 5 species are publicly accessible at NCBI Epigenomics [75, 76]. The human *NRF2* epigenome of primary cells (breast, penis) and H1 stem cell line as well as mouse *Nrf2* CpG island methylation data for sperm, blood, and cerebellum are currently available (http://www.ncbi.nlm.nih.gov/epigenomics). Although no direct evidence of disease-associated epigenetic modulation has been identified in human *NRF2*, various phytochemical NRF2 agonists such as sulforaphane and curcumin have shown their roles in DNA methylation and histone modification (see reviews by Lee and colleagues, e.g., [77]). Taken together, epigenetic modifications of the *NRF2/KEAP1* axle are predicted to cause dysregulation of ARE-mediated cellular defense leading to deleterious health effects, and phytochemical antioxidants as epigenetic modulators for *NRF2* are suggested to be useful in cancer prevention. 

## 6. Conclusions

*NRF2* is evolutionally conserved with high-sequence homology in many species. However, it is a highly mutable gene, and numerous genetic variants have been discovered in human ethnic groups. Importantly, certain SNPs or haplotypes have been identified in various diseases as “at-risk” alleles and are related to functional alterations. In addition to genetic variations, multiple somatic mutations identified in the KEAP1 recognition domain of *NRF2* in cancer cells have been found to be oncogenic due to dysregulation of NRF2 homeostasis by its excess “gain of function”. Epigenetic alteration of the *NRF2* is under investigation and is predicted to have pathogenic influences as learned from mouse and phytochemical agonist studies. Continuous updates of *Nrf2* allelic variants in inbred mouse strains will provide a useful tool for effective experimental designs for models of oxidative disorders to provide insight into the disease mechanisms and intervention strategies.

## Supplementary Material

Supplementary Table S1 includes genetic mutations in human NRF2 focus (7 kb upstream included) compiled from public database. Supplementary Table S2 includes genetic mutations in murine NRF2 focus (5 kb upstream and 2 kb downstream included) collected from public database for 17 inbred strains.

## Figures and Tables

**Table 1:** Gene orthology of NF-E2-related factor 2 across the species.

Species	Human *Homo sapiens *	Cat *Felis catus *	Chimpanzee *Pan troglodytes *	Dog *Canis lupus familiaris *	Cattle *Bos taurus *

Official full name	nuclear factor (erythroid-derived 2)-like 2				
Anthor name	nuclear factor erythroid 2-related factor 2, NF-E2-related factor 2, NFE2-related factor 2				
Anthor name	HEBP1	—	—	—	—
Gene synonyms	*NFE2L2, NRF2 *	*NFE2L2, NRF2 *	*NFE2L2, NRF2 *	*NFE2L2, NRF2 *	*NFE2L2, NRF2 *
Chromosome map	2q31.2	C1	2B	36	2
Genome sequence (NCBI Build)	NC_000002.11 (GRCh37.p10)	NC_108730.1 (Felis_catus-6.2)	NC_006470.3 (Pan_troglodytes-2.1.4)	NC_006618.3 (CanFam3.1)	AC_000159.1 (Bos_taurus_UMD_3.1)
Primary source	HGNC: 7782	—	—	—	BOS_1716
Gene ID mapping (orientation*)	**4780** 178095031−178129859 (−)	**101098812** 165356974−165392728 (−)	**742622** 181721941−181756837 (−)	**478813** 20989206−21021893 (−)	**497024** 19659540−19692048
Ensemble gene ID (mapping)	ENSG00000116044 (178092323−178257425)	—	ENSPTRG00000012677 (181721949−181756285)	ENSCAFG00000013506 (20987569−21022230)	ENSBTAG00000019255 (19659540−19692048)
RefSeq ID, mRNA size	NM_006164.4 (GI: 372620346), 2859 bp	XM_003990893.1 (GI: 410968909), predicted, 2394 bp	XM_001145876.2 (GI: 332814815), predicted, 2850 bp	XM_535975.3 (GI: 345797173), predicted, 2570 bp	NM_001011678.2 (GI: 147904941), 2409 bp
Protein ID (aa/MW^§^)	NP_006155.2 (605/67696)	XP_003990942.1 (606/67797)	XP_001145876.2 (605/67686)	XP_535975.1 (601/67087)	NP_001011678.2 (607/67813)
UniProt ID	Q16236	—	A2T6Y9	—	Q5NUA6.2
Variants^#^	14 transcripts, 12 isoforms	3 transcripts, 3 isoforms	5 transcripts, 5 isoforms	2 transcripts, 2 isoforms	—
Homology% gene/protein	100/100	89.3/91.4	99.9/99.9	89.3/88.8	90.5/89.1

Species	Rhesus monkey *Macaca mulatta *	Chicken *Gallus gallus *	Zebrafish *Danio rerio *	Mouse *Mus musculus *	Rat *Rattus norvegicus *

Official full name	nuclear factor (erythroid-derived 2)-like 2			nuclear factor, erythroid-derived 2, like 2	
Anthor name	nuclear factor erythroid 2-related factor 2, NF-E2-related factor 2, NFE2-related factor 2				
Anthor name	HEBP1	—	—	—	—
Gene synonyms	*NFE2L2, NRF2, Mmu.966 *	*NFE2L2, NRF2 *	*nfe2l2*, *Nrf2*, *Nrf2a*, wu:fc15g09, wu:fj67e03	*Nfe2l2, Nrf2, *AI194320	*Nfe2l2, Nrf2 *
Chromosome map	12	7	9	2C3 (44.75 cM)	3q23
Genome sequence (NCBI Build)	NC_007869.1	NC_006094.3	NC_007120.5 (Zv9)	NC_000068.7 (GRCm38.p1)	NC_005102.3 (Rnor_5.0)
Primary Source	—	CGNC: 49604	ZFIN: ZDB-GENE-030723-2	MGI: 108420	RGD: 620360
Gene ID mapping (orientation*)	**707606** 40848940−40884600 (−)	**396014** 15304546−15320356	**360149** 1643746−1670288	**18024** 75675519−75704641 (−)	**83619** 69041647−69069070 (−)
Ensemble gene ID (mapping)	ENSMMUG00000001861 (40848948−40884174)	ENSGALG00000009240 (16897956−16922637)	ENSDARG00000042824 (1643631−1672715)	ENSMUSG00000015839 (75675513−75704641)	ENSRNOG00000001548 (58366693−58394118)
RefSeq ID, mRNA size	NM_001257607.1 (GI: 383872375), 2335 bp	NM_205117.1 (GI: 45384113), 2555 bp	NM_182889.1 (GI: 33504557), 2149 bp	NM_010902.3 (GI: 76573877), 2469 bp	NM_031789.2 (GI: 402692377), 2305 bp
Protein ID (aa/MW^§^)	NP_001244536.1 (606 /67703)	NP_990448.1 (582/65160)	NP_878309.1 (586/65757)	NP_035032.1 (597/66770)	NP_113977.1 (597/66825)
UniProt ID	F7GPD8	Q90834	Q8JIM1	Q60795	O54968
Variants^#^	2 transcripts, 2 isoforms	1 transcript, 1 isoform	1 transcript, 1 isoform	1 transcript	1 transcript, 1 isoform
Homology% gene/protein	98.8/99.0	72.6/67.4	54.6/49.1	83.4/82.5	83.8/83.2

Refer to http://www.ncbi.nlm.nih.gov/homologene/2412 and reference [14] for cross-species homology. Details of homology scores and sequences can be also obtained by blast against human sequence (http://blast.ncbi.nlm.nih.gov).^#^Further information on transcript variants and protein isoforms are viewable by gene ID at NCBI (http://www.ncbi.nlm.nih.gov/refseq/rsg) and e!Ensemble (http://useast.ensembl.org/index.html) or by protein ID at UniProt (http://www.uniprot.org). ^§^MW: predicted molecular weight from NCBI (MW in UniProt varies slightly.). RefSeq: reference sequence, aa: amino acids, and *gene in complement orientation.

**Table 2:** Protein domains of NF-E2-related factor 2.

Domains	Amino acid positions*		Predicted functions
	Human (605 aa)	Mouse (597 aa)	
Neh2	16–89 DLG motif 17–32, ETGF motif 77–82	16–89	KEAP1 repression through DLG/ETGF motif-DC motif binding, fast, redox-sensitive proteasomal degradation.
Neh4	111–134	111–134	Translocation and transactivation. Phosphorylation or CBP binding.
Neh5	182–209	172–201	
Neh6	337–394 (or 338–388)	328–385	Degron motif-associated constitutive turnover, slow, redox-insensitive (Keap1-independent).
Neh1	435–568 basic motif 503–518 leucine zipper 525–539	427–560 basic motif 494–509 leucine zipper 517–531	Dimerization for nuclear translocation, DNA binding through basic motif-leucine zipper.
Neh3	569–605	561–597	CHD6 binding, stability, or transactivation.

*Varies slightly among publications.

**Table 3:** Genetic mutations in promoter and exons of human *NRF2*.

ID	Map on chromosome 2* (HGVS name)	Position in *Nfe2l2* ^#^ (HGVS name)	Regions (position from mRNA)^#^	Variation class and consequences (HGVS name)^†^	Minor allele frequency (MAF)/ MA counts^‡^ (cohort size)	MAF sources
rs16865105	g.178136629A>C	c.−555−6770T>G	5′Flanking (−6770)	SNP	C = 0.1928/421	1000 Genomes
rs7557529	g.178135097C>T	c.−555−5238G>A	5′Flanking (−5238)	SNP	na	
rs6750320	g.178131796C>T	c.−555−1937G>A	5′Flanking (−1937)	SNP	T = 0.0302/66	1000 Genomes
rs181162518	g.178131774T>C	c.−555−1915A>G	5′Flanking (−1915)	SNP	C = 0.0005/1	1000 Genomes
rs190044775	g.178131746C>T	c.−555−1887G>A	5′Flanking (−1887)	SNP	T = 0.0005/1	1000 Genomes
rs185117338	g.178131704A>G	c.−555−1845T>C	5′Flanking (−1845)	SNP	G = 0.0005/1	1000 Genomes
rs149947189	g.178131697C>T	c.−555−1838G>A	5′Flanking (−1838)	SNP	T = 0.0009/2	1000 Genomes
rs139771244	g.178131625A>G	c.−555−1766T>C	5′Flanking (−1766)	SNP	G = 0.0009/2	1000 Genomes
rs6747203	g.178131604C>G	c.−555−1745G>C	5′Flanking (−1745)	SNP	G = 0.006/13	1000 Genomes
rs193101749	g.178131504T>C	c.−555−1645A>G	5′Flanking (−1645)	SNP	C = 0.0046/10	1000 Genomes
rs190630762	g.178131495G>C	c.−555−1636C>G	5′Flanking (−1636)	SNP	C = 0.0009/2	1000 Genomes
rs183764402	g.178131366C>A	c.−555−1507G>T	5′Flanking (−1507)	SNP	A = 0.0005/1	1000 Genomes
rs191222964	g.178131211G>A	c.−555−1352C>T	5′Flanking (−1352)	SNP	A = 0.0009/2	1000 Genomes
rs187137522	g.178131165T>G	c.−555−1306A>C	5′Flanking (−1306)	SNP	G = 0.0009/2	1000 Genomes
rs182620359	g.178131158A>G	c.−555−1299T>C	5′Flanking (−1299)	SNP	G = 0.0005/1	1000 Genomes
rs4893819 (rs61433302)	g.178131134C>T	c.−555−1275G>A	5′Flanking (−1275)	SNP	C = 0.4263/931	1000 Genomes
rs191547130	g.178131017C>T	c.−555−1158G>A	5′Flanking (−1158)	SNP	T = 0.0005/1	1000 Genomes
rs188422217	g.178131003A>G	c.−555−1144T>C	5′Flanking (−1144)	SNP	G = 0.0014/3	1000 Genomes
rs143047764	g.178130865A>G	c.−555−1006T>C	5′Flanking (−1006)	SNP	G = 0.0069/15	1000 Genomes
rs74432849	g.178130766C>A	c.−555−907G>T	5′Flanking (−907)	SNP	na	
rs11679252	g.178130691C>G	c.−555−832G>C	5′Flanking (−832)	SNP	na	
rs12993217	g.178130516A>G	c.−555−657T>C	5′Flanking (−657)	SNP	na	
rs115644826	g.178130442T>A	c.−555−583A>T	5′Flanking (−583)	SNP	A = 0.0151/33	1000 Genomes
rs140803524	g.178130431G>A	c.−555−572C>T	5′Flanking (−572)	SNP	A = 0.0046/10	1000 Genomes
rs77684420	g.178130427T>C	c.−555−568A>G	5′Flanking (−568)	SNP	C = 0.0339/74 C = 0.190 (84)	1000 Genomes [24]
rs183651094	g.178130336A>T	c.−555−477T>A	5′Flanking (−477)	SNP	T = 0.0018/4	1000 Genomes
rs35652124^§^ (rs57695243)	g.178130073T>C	c.−555−214A>G	5′Flanking (−214)	SNP	C = 0.3512/767 T = 0.429 (84) T = 0.413/181^‡^ C = 0.338/27^‡^ C = 0.351/769	1000 Genomes [24] [22] [23] [25]
rs6706649^§^	g.178130071C>T	c.−555−212G>A	5′Flanking (−212)	SNP	T = 0.078/170 T = 0.048 (84) T = 0.048/21^‡^ T = 0.075/3^‡^	1000 Genomes [24] [22] [23]
rs150648896	g.178130047C>G	c.−555−188G>C	5′Flanking (−188)	SNP	G = 0.0023/5 G = 0.006 (84)	1000 Genomes [24]
rs6721961^§^ (rs117801448)	g.178130037T>C, T>G	c.−555−178A>C, A>G	5′Flanking (−178)	SNP	T = 0.150/328 T = 0.283/124^‡^ T = 0.313/25^‡^ T = 0.321 (84)	1000 Genomes [22] [23] [24]
rs201345604	g.178129924_178129925insG	c.−555-66_−555-65insC	5′Flanking (−66/−65)	Insertion	G = 0.0179/39	1000 Genomes
rs200432479	g.178129741_178 129742delAA	c.−438_−437delTT	exon 1/UTR-5′(118−119)	Deletion	- = 0.0037/8 - = 0.006 (84)	1000 Genomes [24]
rs75485459	g.178129608C>A	c.−304G>T	Exon 1/UTR-5′ (252)	SNP	na	
rs192086766	g.178129466C>T	c.−162G>A	Exon 1/UTR-5′ (394)	SNP	T = 0.022/48	1000 Genomes
—	g.178129442G>A	c.−138C>T	Exon 1/UTR-5′ (418)	SNP	A = 0.012 (84)	[24]
rs71668246	g.178129400delG	c.−96delC	Exon 1/UTR-5′ (460)	Deletion	na	
rs187291840	g.178129399C>T	c.−95G>A	Exon 1/UTR-5′ (461)	SNP	T = 0.0549/120	1000 Genomes
rs143406266	g.178129391_178129393delGGC	c.−89_−87delGCC	Exon 1/UTR-5′ (467−469)	Deletion	- = 0.644/282^‡^ - = 0.589 (84)	[22] [24]
rs182428269	g.178098918G>A	c.127C>T	Exon 2 (682)	Cns (p.Arg43Trp)	A = 0.0005/1	1000 Genomes
rs35248500	g.178098917C>T	c.128G>A	Exon 2 (683)	Cns (p.Arg43Gln)	T = 0.006/13	1000 Genomes
rs1135118	g.178098831C>T	c.214G>A	Exon 2 (769)	Cns (p.Ala72Thr)	T^‡^	[23]
rs199691660	g.178098829A>T	c.216T>A	Exon 2 (771)	Cs (p.Ala72=)	na	
—	g.178098769G>A	c.276C>T	Exon 2 (831)	Cs (p. Ile 92=)	T^‡^	[23]
rs5031039	g.178098750A>G	c.295T>C	Exon 2 (850)	Cns (p.Ser99Pro)	G = 0^‡^	[23]
rs200239262	g.178098017C>G	c.363G>C	Exon 3 (918)	Cns (p.Gln121His)	na	
rs183034165	g.178098008T>C	c.372G>A	Exon 3 (927)	Cs (p.Ala124=)	T = 0.0009/2 T = 0.006 (84)	1000 Genomes [24]
rs199970826	g.178097996C>T	c.384G>A	Exon 3 (939)	Cs (p.Pro128=)	T = 0.0005/1	1000 Genomes
rs201992337	g.178097260C>T	c.454G>A	Exon 4 (1009)	Cns (p.Glu152Lys)	na	
rs201589693	g.178097251C>T	c.463G>A	Exon 4 (1018)	Cns (p.Val155Ile)	T = 0.0005/1	1000 Genomes
rs35577826	g.178097185A>C	c.529T>G	Exon 4 (1084)	Cns (p.Leu177Val)	C = 0.0014/3 A < 0.005^‡^	1000 Genomes [23]
rs181513314	g.178096710C>T	c.621G>A	Exon 5 (1176)	Cn (p.Leu207=)	T = 0.0005/1	1000 Genomes
rs60132461	g.178096675T>C	c.656A>G	Exon 5 (1211)	Cns (p.Lys219Arg)	C = 0.0018/4	1000 Genomes
rs139187151	g.178096634G>A	c.697C>T	Exon 5 (1252)	Cns (p.Pro233Ser)	A = 0.0005/1 A = 0.012^‡^ (84)	1000 Genomes [24]
rs35557421	g.178096620delT	c.711delA	Exon 5 (1266)	Frame shift/deletion (p.Lys237 = fs)	na	
rs34154613	g.178096529C>T	c.802G>A	Exon 5 (1357)	Cns (p.Val268Met)	T = 0.0018/4	1000 Genomes
rs141363120	g.178096406G>A	c.925C>T	Exon 5 (1480)	Cns (p.Leu309Phe)	A = 0.0037/8	1000 Genomes
rs201661476	g.178096380A>C	c.951T>G	Exon 5 (1506)	Cns (p.Ile317Met)	na	
rs199673454	g.178096309T>A	c.1022A>T	Exon 5 (1577)	Cns (p.Asp341Val)	A = 0.0005/1	1000 Genomes
rs35007548	g.178096299G>A	c.1032C>T	Exon 5 (1587)	Cs (p.Ser344=)	A = 0.0009/2	1000 Genomes
rs200209692	g.178096287T>C	c.1044A>G	Exon 5 (1599)	Cs (p.Leu348=)	C = 0.0005/1	1000 Genomes
—	g.178096237C>A	c.1094G>T	Exon 5 (1649)	Cns (p.Ser365Ile)	A = 0.125/273 A = 0.006^‡^ (84)	PubMed [24]
rs201214197	g.178096171C>T	c.1160G>A	Exon 5 (1715)	Cns (p.Ser387Asn)	T = 0.0005/1	1000 Genomes
rs200494292	g.178096165T>C	c.1166A>G	Exon 5 (1721)	Cns (p.Lys389Arg)	na	
rs186171287	g.178096115G>A	c.1216C>T	Exon 5 (1771)	Cns (p.Pro406Ser)	A = 0.0005/1	1000 Genomes
rs182276775	g.178096062C>A	c.1269G>T	Exon 5 (1824)	Cns (p.Glu423Asp)	A = 0.0005/1	1000 Genomes
rs201560221	g.178096048T>C	c.1283A>G	Exon 5 (1838)	Cns (p.Lys428Arg)	na	
rs189238236	g.178096043A>G	c.1288T>C	Exon 5 (1843)	Cs (p.Leu430=)	G = 0.0005/1	1000 Genomes
rs184287392	g.178096022G>A	c.1309C>T	Exon 5 (1864)	Cns (p.Arg437Trp)	A = 0.0005/1	1000 Genomes
rs201871588	g.178095986G>A	c.1345C>T	Exon 5 (1900)	Cns (p.Ag449Cys)	na	
rs181294188	g.178095985T>C	c.1346G>A	Exon 5 (1901)	Cns (p.Arg449His)	T = 0.0009/2	1000 Genomes
rs201690466	g.178095973T>A	c.1358A>T	Exon 5 (1913)	Cns (p.His453Leu)	A = 0.0005/1	1000 Genomes
rs1057044 (rs52789869)	g.178095781C>T	c.1550G>A	Exon 5 (2105)	Cns (p.Arg517Lys)	T^‡^	[23]
rs200750800	g.178095603A>G	c.1728T>C	Exon 5 (2283)	Cs (p.Tyr576=)	na	
rs200175942	g.178095567A>G	c.1764T>C	Exon 5 (2319)	Cs (p.Asp588=)	G = 0.0005/1	1000 Genomes
rs77547666	g.178095495G>C	c.*18C>G	Exon 5/UTR-3′ (2391)	SNP	C = 0.0069/15	1000 Genomes
rs73031353	g.178095425T>C	c.*88A>G	Exon 5/UTR-3′ (2461)	SNP	C = 0.0018/4	1000 Genomes
rs6759443	g.178095345T>C	c.*168A>G	Exon 5/UTR-3′ (2541)	SNP	C = 0.0041/9	1000 Genomes
rs188674558	g.178095279C>A	c.*234G>T	Exon 5/UTR-3′ (2607)	SNP	A = 0.0005/1	1000 Genomes
rs77685897	g.178095247A>G	c.*266T>C	Exon 5/UTR-3′ (2639)	SNP	G = 0.0009/2	1000 Genomes
rs1057092	g.178095162T>G	c.*351A>C	Exon 5/UTR-3′ (2724)	SNP	na	
rs3197704	g.178095162T>G	c*351A>C	Exon 5/UTR-3′ (2724)	SNP	na	
rs184701151	g.178095159T>C	c.*354A>G	Exon 5/UTR-3′ (2727)	SNP	C = 0.0027/6	1000 Genomes
rs11543307	g.178095153A>G	c.*360T>C	Exon 5/UTR-3′ (2733)	SNP	na	
rs111874043	g.178095146A>G	c.*367T>C	Exon 5/UTR-3′ (2740)	SNP	na	
rs34012004	g.178095102A>C	c.*411T>G	Exon 5/UTR-3′ (2784)	SNP	C = 0.071	[24]
rs201481890	g.178095090_178095091insT	c.*422_*423insA	Exon 5/UTR-3′ (2795−2796)	Insertion	na	
rs3082500	g.178095089_178095090delTT, delTTinsT	c.*423_*424delA AinsA	Exon 5/UTR-3′ (2796−2797)	Deletion, insertion	na	
rs71792546 (rs71796710)	g.178095079delT	c.*425delA	Exon 5/UTR-3′ (2798)	Deletion	na	
rs1057106	g.178095078A>C, A>T	c.*435T>A, T>G	Exon 5/UTR-3′ (2808)	SNP	na	
rs34176791	g.178095076A>C	c.*437T>G	Exon 5/UTR-3′ (2810)	SNP	C = 0.0023/5	1000 Genomes
rs35911553	g.178095045C>T	c.*468G>A	Exon 5/UTR-3′ (2841)	SNP	T = 0.0069/15	1000 Genomes

Sequence variations in upstream and exons of human *NRF2* from 655 variations available in public database as of December, 2012 (583 active, some SNPs merged, ≥14 SNPs cited in PubMed). *NCBI reference sequence NC_000002.11 (Homosapiens chromosome 2, GRCh37.p10 primary assembly) spanning 178,095,033–178,129,859 bp (complement, 34,827 bp for exons and introns). 5′-Flanking regions start at 178,129,860 bp (−1) in transcript variant 1. HGVS: Human Genome Variation Society. ^#^Positions in variant 1 (NM_006164.4), 2859 bp. ^§^SNPs cited in PubMed. Exon 1: 178,129,859–178,129,260 (600 bp, TTS = 178,129,304), exon 2: 178,098,999–178,098,733 (267 bp), exon 3: 178,098,067–178,097,978 (90 bp), exon 4: 178,097,311–178,097,120 (192 bp), and exon 5: 178,096,736–178,095,033 (1704 bp). ^†^Protein amino acid (aa) residues in isoform 1 (NP_006155.2, 605 aa). Cns: coding-nonsynonymous. Cs: coding-synonymous.^‡^heterozygosity detected. na: not available.

**Table 4:** Human *NRF2 *SNPs associated with disease risk.

ID	Map on chromosome 2 (HGVS name)	Region/class	Disease association and references	Risk alleles and statistics	Ethnic group (number of case)

rs7557529	g.178135097C>T	−5238**G>A**	Parkinson's disease (2010 [26])	G = 0.718/252^‡^ in haplotype OR = 0.90 or 0.40; CI = 0.60–1.40 or 0.3–0.6	Swedish/Polish Caucasian (357)

rs2886162	g.178133165A>G	−3306**T>C**	Breast cancer survival (2012 [27])	T/T = 0.324 OR = 1.687; CI = 1.105–2.75	Finland KBCP (452)

			Chronic gastritis, gastric ulcer (2007 [28])	G^‡^ G^‡^ in haplotype for ulcer (OR = 2.52; CI = 1.19–5.45)	Japanese (159)
			P14 methylation in gastric cancer *H*. pylori infection (2008 [29])	G in haplotype OR = 2.90; CI = 1.14–7.36	Japanese (209)
			Gastric cancer in *H. *pylori-negative cases (2008 [30])	A in haplotype *P* = 0.022	Japanese (209)
rs35652124 (rs57695243)	g.178130073T>C	−214**A>G** (previously −653 [23] or −686 [22])	Ulcerative colitis (2008 [31])	A/G (OR = 0.45; CI = 0.22–0.93) G (OR = 2.57; CI = 1.01–6.60)	Japanese (89)
			Lupus with nephritis in female (2010 [25])	G/A OR = 1.81; CI = 1.04–3.12	Mexican mestizo (362)
			Parkinson's disease (2010 [26])	A = 0.884/312^‡^ in haplotype OR = 0.9 or 0.4; CI = 0.60–1.40 or 0.30–0.60	Swedish/Polish Caucasian (357)
			COPD (2010 [32])	G = 0.52^‡^ hazard ratio = 0.95; CI = 0.91–0.99 (Haplotype)	German (69)

			Gastric ulcer (2007 [28])	G^‡^ in haplotype OR = 2.52; CI = 1.19–5.45	Japanese (159)
			P14 methylation in gastric cancer *H. * pylori infection (2008 [29])	G in haplotype OR = 2.90; CI = 1.14–7.36	Japanese (209)
			Gastric cancer in *H. * pylori -negative cases (2008 [30])	G in haplotype, *P* = 0.022	Japanese (209)
rs6706649	g.178130071C>T	−212**G>A** (previously −651 [23] or −684 [22])	Ulcerative colitis (2008 [31])	A/G (OR = 0.45; CI = 0.22–0.93) G (OR = 2.57; CI = 1.01–6.60)	Japanese (89)
			Maternal acetaminophen and asthma (2010 [33])	A = 0.232/1137^‡^ OR = 1.73; CI = 1.22, 2.45	UK ALSPAC (>4000 mothers, >5000 children)
			COPD (2010 [32])	G = 0.98^‡^ in haplotype hazard ratio = 0.95; CI = 0.91–0.99	German (69)
			Parkinson's disease (2010 [26])	G = 0.972/343^‡^ in haplotype OR = 0.9 or 0.4; CI = 0.60–1.40 or 0.3–0.6	Swedish/Polish Caucasian (357)

			Acute lung injury following trauma (2007 [23])	C/A = 0.119^‡^ OR = 6.44; CI = 1.34–30.80	Caucasian/African-American (164)
			Annual FEV_1_ decline (2011 [34])	A = 0.082 in haplotype	Japanese (915)
			Vitiligo (2008 [35])	C/A, A/A OR = 1.724; CI = 1.35–2.21	Chinese (300)
			COPD (2010 [32])	G = 0.73^‡^ in haplotype hazard ratio = 0.95; CI = 0.91–0.99	German (69)
rs6721961 (rs117801448)	g.178130037T>C, T>G	−178**A>G**, **A>C** (previously −617 [23] or −650 [22])	Parkinson's disease (2010 [26])	C = 0.980/346^‡^ in haplotype OR = 0.9 or 0.4; CI = 0.60–1.40 or 0.30–0.60	Swedish/Polish Caucasian (357)
			Postmenopausal venous thromboembolism (2011 [36])	A = 0.333/11 OR = 2.5; CI = 3.70–85.70	Caucasian (161)
			Breast cancer survival and NRF2 protein expression (2012 [27])	A/A OR = 4.656; CI = 1.35–16.06	Finland KBCP (452)
			Acute lung injury-related mortality following systemic inflammatory response syndrome (2012 [37])	G/G OR = 9.73; CI = 1.27–74.80	Caucasian (750)
			Infection-induced asthma (2012 [38])	A/C OR = 0.437; CI = 0.28–0.80	Hungarian Caucasian/Gypsy (307)

rs143406266	g.178129391_178 129393delGGC	Exon 1 (467–469**GCC**)	COPD (2010 [32])	GCC_4_ = 0.53^‡^ hazard ratio = 0.95; CI = 0.91–0.99	Taiwanese (69)

rs2886161	g.178127839T>C*	Intron 1	Parkinson's disease (2010 [26])	T = 0.878/309^‡^ in haplotype OR = 0.9 or 0.4; CI = 0.6–1.4 or 0.3–0.6	Swedish/Polish Caucasian (357)

rs2364723	g.178126546**G>C**	Intron 1	Basal and smoker FEV_1_ (2009 [39])	CI = −63.60~−17.80, C = 0.525^‡^ Also as haplotype	Netherland (2542)

rs2364722	g.178124787**A>G**	Intron 1	Annual FEV_1_ decline (2011 [34])	A = 0.082 in haplotype	Japanese (915)

rs13001694	g.178118990A**>G**	Intron 1	Basal and smoker FEV_1_ (2009 [39])	G = 0.401/1578^‡^ in haplotype	Netherland (2542)

			Breast cancer [40]	T with NQO1/NOS3/HO1 risk alleles: OR = 1.56; CI = 0.97–2.51	Caucasian and others (505)
rs1806649 (rs58745895)	g.178118152**C>T**	Intron 1	Basal and smoker FEV_1_ (2009 [39])	T = 0.263/1119^‡^ in haplotype CI = −87.30–(−1.70)	Netherland (2542)
			Parkinson's disease (2010 [26])	T = 0.422/148^‡^ in haplotype OR = 0.9 or 0.4; CI = 0.60–1.40 or 0.30–0.60	Swedish/Polish Caucasian (357)
			Particulate matter and asthma/COPD admission (2012 [41])	C with low vitamin C level (OR = 3.1; CI = 1.50–6.30)	UK (209)

rs4243387 (rs60038464)	g.178117765**C>T**	Intron 1	Basal and smoker FEV_1_ (2009 [39])	T = 0.091/425^‡^ in haplotype	Netherland (2542)

rs1962142 (rs58448508)	g.178113484**A>G**	Intron 1	Annual FEV_1_ decline (2011 [34])	A = 0.082 in haplotype	Japanese (915)
			Breast cancer NRF2 and ARE expression (2012 [27])	A (*P* = 0.036)	Finland KBCP (452)

rs6726395 (rs57309289)	g.178103229**A>G**	Intron 1	Smoking-related FEV_1_ decline and annual FEV_1_ decline (2011 [34])	G = 0.884^‡^ A = 0.082 in haplotype	Japanese (915)
			Basal and smoker FEV_1_ (2009 [39])	G = 0.464/1764^‡^ in haplotype	Netherland (2542)

rs2001350 (rs17515179, rs60883775)	g.178100425**C>T**	Intron 1	Annual FEV_1_ decline (2011 [34])	T = 0.082 in haplotype	Japanese (915)
			Parkinson's disease (2010 [26])	T = 0.986/350^‡^ in haplotype OR = 0.9 or 0.4; CI = 0.60–1.40 or 0.30–0.60	Swedish/Polish Caucasian (357)

rs10183914 (rs58731187, rs61374844)	g.178097666**C>T**	Intron 3	Parkinson's disease (2010 [26])	T = 0.536/188^‡^ in haplotype OR = 0.9 or 0.4; CI = 0.60–1.40 or 0.30–0.60	Swedish/Polish Caucasian (357)

rs2706110	g.178092162**T>C**	3′Flanking	Breast cancer (2012 [27])	T/T OR = 2.079; CI = 1.18–3.68	Finland KBCP (452)

rs2588882	g.178087165**G>T**	3′Flanking	Infection-induced asthma (2012 [38])	T/G OR = 0.290; CI = 0.13–0.62	Hungarian Caucasian/Gypsy (307)

−686 in reference [22] = −653 in reference [23] = currently −214; −684 in reference [22] = −651 in reference [23] = currently −212; −651 in reference [22] = −617 in reference [23] = currently −178. Chromosome contig (intron, 3′flanking) or reversed (5′flanking promoter) alleles in bold have been used in the text and Table. OR: odds ratio. CI: 95% confidence interval.

**Table 5:** Mouse upstream and exon variations of *Nfe2l2* locus in 17 inbred strains. Reference sequence is C57BL/6J (B6, strain 1) genome (GI: 149338249, 75547698–75513576). SNP allele and genotypes are shown as chromosome contig sequence. Strains 2: 129S1/SvlmJ, 3: A/J, 4: AKR/J, 5: BALB/cJ, 6: C3H/HeJ, 7: C57BL6/NJ, 8: CAST/EiJ, 9: CBA/J, 10: DBA/2J, 11: FVB/NJ, 12: LP/J, 13: NOD/ShiLtJ, 14: NZO/HILtJ, 15: PWK/PhJ, 16: SPRET/EiJ, 17: WSB/EiJ.

SNP ID	Chr2 location (bp)	SNP allele	Region	Location from TSS/reversed SNP allele	Consequences	Strains																
						1 B6	2	3	4	5	6	7	8	9	10	11	12	13	14	15	16	17
rs256608517	75705565	G/A (C/T)	5′Flanking	−924	NC	G	G	G	G	G	G	G	G	G	G	G	G	G	G	A	A	G
rs47274959	75705562	A/T (T/A)	5′Flanking	−921	NC	A	T	T	A	T	T	A	A	T	T	A	T	A	T	T	T	A
rs216940398	75705554–75705555	T add (A add)	5′Flanking	−913 : −914	NC	CG																
rs239114134	75705540	C/T (G/A)	5′Flanking	−899	NC	C	T	T	C	T	T	C	C	T	T	C	T	C	T	C	T	C
rs46461765	75705528	T/G (A/C) ?oA/C?	5′Flanking	−887	NC	T	G	G	T	G	G	T	T	G	G	T	G	T	G	G	G	T
rs51449853	75705525	C/A (G/T)	5′Flanking	−884	NC	C	A	A	C	A	A	C	C	A	A	C	A	C	A	C	C	C
rs249093111	75705512	C/T (G/A)	5′Flanking	−871	NC	C	T	T	C	T	T	C	C	T	T	C	T	C	T	T	T	C
rs263745200	75705510	C/G (G/C)	5′Flanking	−869	NC	G	G	G	G	G	G	G	G	G	G	G	G	G	G	C	G	G
rs45651867	75705498	T/G (A/C)	5′Flanking	−857	NC	T	G	G	T	G	G	T	T	G	G	T	G	T	G	G	G	T
rs219997531	75705495	G/A (C/T)	5′Flanking	−854	NC	G	G	G	G	G	G	G	G	G	G	G	G	G	G	G	A	G
rs251918379	75705451	T/A (A/T)	5′Flanking	−810	NC	T	T	T	T	T	T	T	T	T	T	T	T	T	T	T	A	T
rs27978306	75705449	T/A (A/T)	5′Flanking	−808	NC	T	A	A	T	A	A	T	T	A	A	T	A	T	A	T	T	T
rs216087412	75705429	T/G (A/C)*	5′Flanking	−788	NC	T*	T*	T*	T*	T*	T*	T*	T*	T*	T*	T*	T*	T*	T*	G	T*	T*
rs261229914	75705423–75705424	AG del (CT del)	5′Flanking	−783 : −784	NC	AG																
rs27978307	75705400	C/T (G/A)	5′Flanking	−759	NC	C	C	C	C	C	C	C	C	C	C	C	C	C	C	T	C	C
rs243167395	75705359	G/A (C/T)	5′Flanking	−718	NC	G	G	G	G	G	G	G	G	G	G	G	G	G	G	G	A	G
rs27978308	75705307	G/A (C/T)	5′Flanking	−666	NC	G	G	G	G	G	G	G	G	G	G	G	G	G	G	A	G	G
rs254744098	75705294	A/T (T/A)	5′Flanking	−653	NC	A	A	A	A	A	A	A	A	A	A	A	A	A	A	A	T	A
rs228133419	75705179	G/T (C/A)	5′Flanking	−538	NC	G	G	G	G	G	G	G	G	G	G	G	G	G	G	G	T	G
rs214719520	75705111	G/A (C/T)	5′Flanking	−470	NC	G	G	G	G	G	G	G	G	G	G	G	G	G	G	G	A	G
rs244087730	75705101	G/A (C/T)	5′Flanking	−460	NC	G	G	G	G	G	G	G	G	G	G	G	G	G	G	G	A	G
rs227781699	75705081	C/G (G/C)	5′Flanking	−440	NC	C	C	C	C	C	C	C	C	C	C	C	C	C	C	G	C	C
rs257870353	75705067	C/T (G/A)	5′Flanking	−426	NC	C	C	C	C	C	C	C	C	C	C	C	C	C	C	T	C	C
rs244877440	75705022	A/G (T/C)	5′Flanking	−381	NC	A	A	A	A	A	A	A	A	A	A	A	A	A	A	A	G	A
rs234628138	75704973	A/C (T/G)	5′Flanking	−332	NC	A	A	A	A	A	A	A	A	A	A	A	A	A	A	A	C	A
rs247275247	75704961	G/A (C/T)	5′Flanking	−320	NC	G	G	G	G	G	G	G	G	G	G	G	G	A	G	G	G	G
rs247519047	75704922	G del (C del)	5′Flanking	−281	NC	G																
rs27978309 (rs51915758)	75704887	G/A/C (C/T/G)	5′Flanking	−246	NC	G	A	A	G	A	A	G	G	A	A	G	A	G	A	C	C	G
rs218139102	75704879	C/A (G/T)	5′Flanking	−238	NC	C	C	C	C	C	C	C	C	C	C	C	C	C	C	A	C	C
rs251990355	75704870	T/C (A/G)	5′Flanking	−229	NC	T	T	T	T	T	T	T	T	T	T	T	T	T	T	T	C	T
rs238746955	75704794	A/G (T/C)	5′Flanking	−153	NC	A	A	A	A	A	A	A	A	A	A	A	A	A	A	G	A	A
rs221664405	75704786	A/G (T/C)	5′Flanking	−145	NC	A	A	A	A	A	A	A	A	A	A	A	A	A	A	A	G	A
rs217054035	75704766–75704767	GAA add (TTC add)	5′Flanking	−125 : −126	NC	GA																
rs248182931	75704759–75704760	G add (C add)	5′Flanking	−118 : −119	NC	AG																
rs217197904	75704744	G/A (C/T)	5′Flanking	−103	(+)**Sp1**[23]	G	A	A	G	A	A	G	G	A	A	G	A	A	A	G	G	A
rs264493649	75704704	G del (C del)	5′Flanking	−63	NC	G																
rs240481112	75704682	C/T (G/A)	5′Flanking	−41	NC	C	C	C	C	C	C	C	C	C	C	C	C	C	C	C	T	C
rs27978310	75704617	A/G (T/C)	UTR-5	25	NC	A	A	A	A	A	A	A	A	A	A	A	A	A	A	G	G	A
rs27978311	75704610	C/G (G/C)	UTR-5	32	NC	G	G	G	G	G	G	G	G	G	G	G	G	G	G	G	G	C
rs27978312	75704605	G/A (C/T)	UTR-5	37	NC	G	A	A	G	A	A	G	G	A	A	G	A	G	A	G	G	G
rs214784220	75704499	A/G (T/C)	UTR-5	143	NC	A	A	A	A	A	A	A	A	A	A	A	A	A	A	G	G	A
rs244146318	75704498	G/T (C/A)	UTR-5	144	NC	G	G	G	G	G	G	G	G	G	G	G	G	G	G	G	T	G
rs27978313	75704497	G/C (C/G)	UTR-5	145	NC	G	C	C	G	C	C	G	C	C	C	G	C	C	C	C	C	C
rs215431944	75704493	C/T (G/A)	UTR-5	149	NC	C	C	C	C	C	C	C	C	C	C	C	C	C	C	T	C	C
rs257015697	75704449	G/A (C/T)	UTR-5	193	NC	G	G	G	G	G	G	G	G	G	G	G	G	G	G	G	A	G
rs252234782	75704419	G/T (C/A)	UTR-5	223	NC	G																
rs13460861	75679262	A/G (T/C)	Exon2	446	Cns F71L	A	G	G	A	G	G	A	G	G	G	A	G	A	G	G	G	G
rs13460859	75679202	G/A (C/T)*	Exon2	506	Cs H91=	G	A	A	G	A	A	G	G	A	A	G	A	G	A	G	G	G
rs27978436	75679187	G/A (C/T)*	Exon2	521	Cs T96=	G	G	G	G	G	G	G	A	G	G	G	G	G	G	A	G	G
rs226131070	75679178	G/A (C/T)	Exon2	530	Cs S99=	G	G	G	G	G	G	G	G	G	G	G	G	G	G	G	A	G
rs227683834	75678576	A/T (T/A)	Exon3	547	Cns V105D	A	A	A	A	A	A	A	A	A	A	A	A	A	A	A	T	A
rs257321187	75678506	T/C (A/G)	Exon3	617	Cs P128=	T	T	T	T	T	T	T	C	T	T	T	T	T	T	T	T	T
rs27978444	75678500	T/A (A/T)	Exon3	623	Cs V130=	T	T	T	T	T	T	T	A	T	T	T	T	T	T	A	T	T
rs227743136	75677686	G/A (C/T)	Exon4	662	Cs H143=	G	G	G	G	G	G	G	G	G	G	G	G	G	G	G	A	G
rs215327202	75677664	C/G (G/C)	Exon4	684	Cns A151P	C	C	C	C	C	C	C	G	C	C	C	C	C	C	C	C	C
rs247539755	75677663	G/A (C/T)	Exon4	685	Cns A151V	G	G	G	G	G	G	G	A	G	G	G	G	G	G	G	G	G
rs233164668	75677640	T/C (A/G)	Exon4	708	Cns N159D	T	T	T	T	T	T	T	T	T	T	T	T	T	T	T	C	T
rs234095816	75677162–75677160	GCT del (AGC del)	Exon5	826−828	Cds-indel Q198	GCT																
rs27978452	75677145	C/T (G/A)	Exon5	843	Cns A204T	C	C	C	C	C	C	C	C	C	C	C	C	C	C	C	C	T
rs225047937	75677086–75677078	GAGATCGAT del (ATGCATCTC del)	Exon5	902−910	Cds-indel (S224/I225/S226)	GAG ATC GAT																
rs241469868	75676998	T/A (G/T)	Exon5	990	Cn T253S	T	T	T	T	T	T	T	T	T	T	T	T	T	T	T	A	T
rs221602571	75676923	A/C (T/G)	Exon5	1065	Cns S278A	A	A	A	A	A	A	A	A	A	A	A	A	A	A	A	C	A
rs252576472	75676876	G/A (C/T)	Exon5	1112	Cs S293=	G	G	G	G	G	G	G	G	G	G	G	G	G	G	G	A	G
rs250802933	75676841	T/C (A/G)	Exon5	1147	Cns D305G	T	T	T	T	T	T	T	T	T	T	T	T	T	T	T	C	T
rs225425698	75676792	C/T (G/A)	Exon5	1196	Cs P321=	C	C	C	C	C	C	C	C	C	C	C	C	C	C	C	T	C
rs13459064	75676732	C/T (G/A)	Exon5	1256	Cs T341=	C	T	T	C	T	T	C	T	T	T	C	T	C	T	T	C	C
rs27978453	75676717	C/T (G/A)	Exon5	1271	Cs A346=	C	C	C	C	C	C	C	C	C	C	C	C	C	C	T	C	C
rs214335034	75676636	A/G (T/C)	Exon5	1352	Cs D373=	A	A	A	A	A	A	A	A	A	A	A	A	A	A	A	G	A
rs249583644	75676603	A/C (T/G)	Exon5	1385	Cs P384=	A	A	A	A	A	A	A	A	A	A	A	A	A	A	A	C	A
rs27978454	75676592	G/A (C/T)	Exon5	1396	Cns P388L	G	G	G	G	G	G	G	G	G	G	G	G	G	G	A	G	G
rs215384328	75676589	G/C (C/G)	Exon5	1399	Cns A389G	G	G	G	G	G	G	G	G	G	G	G	G	G	G	G	C	G
rs251728286	75676571	G/A (C/T)	Exon5	1417	Cns T395I	G																
rs247602334	75676567	T/C (A/G)	Exon5	1421	Cs V396=	T	T	T	T	T	T	T	T	T	T	T	T	T	T	T	C	T
rs234216231	75676530	T/C (A/G)	Exon5	1458	Cns M409V	T	T	T	T	T	T	T	T	T	T	T	T	T	T	T	C	T
rs212904337	75676526	C/T (G/A)	Exon5	1462	Cns R410H	C	C	C	C	C	C	C	C	C	C	C	C	C	C	C	T	C
rs252650779	75676522	T/C (A/G)	Exon5	1466	Cs E411=	T	T	T	T	T	T	T	T	T	T	T	T	T	T	T	C	T
rs231273560	75676516	T/C (A/G)	Exon5	1472	Cs Q413=	T	T	T	T	T	T	T	T	T	T	T	T	T	T	T	C	T
rs257886949	75676413	G/T (C/A)	Exon5	1575	Cs R448=	G	G	G	G	G	G	G	G	G	G	G	G	G	G	G	T	G
rs241537608	75676404	G/C (C/G)	Exon5	1584	Cns L451V	G	G	G	G	G	G	G	G	G	G	G	G	G	G	G	C	G
rs227619071	75676312	T/C (A/G)	Exon5	1676	Cs Q481=	T	T	T	T	T	T	T	T	T	T	T	T	T	T	T	C	T
rs258913831	75676290	A/G (T/C)	Exon5	1698	Cs L489=	A	A	A	A	A	A	A	A	A	A	A	A	A	A	A	G	A
rs225593319	75676126	A/T (T/A)	Exon5	1862	Cns H543Q	A																
rs4223233	75676105	G/A (C/T)	Exon5	1883	Cs S550=	G	G	G	G	G	G	G	A	G	G	G	G	G	G	G	G	G
rs4223232	75676032	G/T (C/A)	Exon5	1956	Cns L575M	G																
rs4223231	75675816	C/G	UTR-3	2172	NC	G	G	G	G	G	G	G	G	G	G	G	G	G	G	G	C	G
rs1346860	75675682	A/T	UTR-3	2306	NC	A																

Sequence variations in mouse *Nrf2* were obtained from Mouse Phenome Database (http://phenome.jax.org/SNP) and NCBI SNP database (http://www.ncbi.nlm.nih.gov/SNP). NCBI reference sequence is *Mus Musculus* strain C57BL/6J chromosome 2, GRCm38.p1 (NC_000068.7, GI: 372099108. 75,704,641–75,675,519,29,123 bp). Total 968 genetic mutations are reported in *Nrf2* gene and 5 kb upstream (≥75,704,642; −1) and 2 kb downstream (≤75,675,512) sequences as of January 2003. All SNPs and alleles are presented as genomic contig (reversed sequence indicated). Exon 1: 75,704,641–75,704,364 (278 bp, TTS = 75,704,408), exon 2: 75,679,431–75,679,165 (267 bp), exon 3: 75,678,579–75,678,490 (90 bp), exon 4: 75,677,713–75,677,546 (168 bp), and exon 5: 75,677,184–75,678,849 (1,666 bp). Protein amino acid (aa) residues in NP_006155.2 (597 aa). Cs: coding-synonymous. Cns: coding-nonsynonymous. Amino acid A-alanine, D-aspartic acid, E-glutamic acid, F-phenylalanine, G-glycine, H-histidine, I-isoleucine, L-leucine, and M-methionine. N-asparagine, P-proline, Q-glutamine, R-arginine, S-serine, T-threonine, and V-valine. *Errors in databases fixed. NC: not confirmed.

**Table 6:** *NRF2* somatic mutations revealed in various human cancers.

Domain	Amino acid residues			DNA mutation	Cancer types (cases)	References
	Locus	Wild type	Mutant			
DLG motif	24	W (Trp)	C (Cys)	c.72G>C/G>T	NSCLC, neck, ESC	[54, 62]
			K (Lys)	c.72T>C	ESC	[62]
	26	Q (Gln)	E (Glu)	c.76C>G	NSCLC, ESC	[54, 62]
	27	D (Asp)	G (Gly)*	c.80G>A	NSCLC	[60]
			Y (Tyr)	c.79G>T	ESC	[61]
	28	I (Ile)	T (Thr)	c.83C>T	NSCLC	[54]
	29	D (Asp)	G (Gly)	c.86A>G	Head and neck, ESC	[54, 62]
			H (His)	c.85G>C	NSCLC, laynx	[60, 61]
	30	L (Leu)	F (Phe)	c.88C>T	NSCLC, ESC	[54, 62]
	31	G (Gly)	A (Ala)	c.92G>C	NSCLC, ESC, skin	[54, 60–62]
	32	V (Val)	T (Thr)	c.95T>G	NSCLC	[54]
			del	c.93_95delAGT	ESC	[61]
	33–36	S-R-E-V	S-R-E-V-S-R-E-V*	c.97_108dupAGTCGAGCCGTA	ESC	[61]
	34	R (Arg)	Q (Gln)	c.101G>A	NCSCL	[54, 60, 61]
			P (Pro)	c.101G>C	NSCLC	[63]

ETGF motif	75	Q (Gln)	H (His)	c.225A>C	Head and neck, ESC	[54, 62]
	77	D (Asp)	V (Val)	c.230A>T	NSCLC, ESC	[54, 60, 62]
			G (Gly)	c.230A>G	ESC	[62]
			A (Ala)	c.230A>C	NSCLC	[61]
			N (Asn)	c.229G>A	Larynx	[61]
	78	E (Glu)	K (Lys)	c.232G>A	NSCLC, ESC	[54, 62]
	79	E (Glu)	K (Lys)	c.235G>A	NSCLC, ESC	[54, 61, 62]
			Q (Gln)	c.235G>C	NSCLC, ESC	[54, 60–62]
			G (Gly)	c.236A>G	Larynx	[61]
			E-E	c.234_236dupAGA	ESC	[61]
	80	T (Thr)	K (Lys)	c.239C>A/C>G	NSCLC, ESC	[54, 61, 62]
	80	T (Thr)	P (Phe)	c.238A>C	ESC	[62]
			I (Ile)	c.239C>T	Head and neck	[54]
			A (Ala)	c.238A>G	NSCLC	[63]
	81	G (Gly)	V (Val)	c.242G>T	ESC	[61]
			D (Asp)	c.242G>A	NSCLC, ESC	[60–62]
	82	E (Glu)	D (Asp)	c.246A>T	ESC, oral cancer cell line	[54, 62]
			G (Gly)	c.245A>G	NSCLC	[54]
			Q (Gln)*	c.244G>C	NSCLC, ESC	[60, 61]
			V (Val)*	c.245A>T	ESC	[61]
	83	F (Phe)	L (Leu)*	c.247T>C	NSCLC	[60]

NSCLC: non-small cell lung cancer, ESC: esophageal squamous cancer, and *errors in reference fixed. Number of cases: 82 NSCLC and 10 ESC in [62]; 125 NSCLC, 70 ESC, 23 larynx, and 17 skin in [61]; 103 NSCLC and 12 head and neck in [54]; 90 NSCLC in [63]; 103 NSCLC in [60].

## Abbreviations

AD: Alzheimer's disease
ALI: acute lung injury
ARE: antioxidant response element
bZIP: basic leucine zipper
cds: coding DNA sequences
CI: confidence interval
COPD: chronic obstructive pulmonary disease
ESC: esophageal squamous cancer
FEV_1_: forced expiratory volume in one second
Gbp: giga base pairs
GST: glutathione S-transferase
GWAS: genome-wide association study
HGVS: Human Genome Variation Society
HO-1: heme oxygenase-1
*H. pylori*: *Helicobacter pylori*
KEAP1: Kelch-like ECH activating protein 1
MPD: Mouse Phenome Database
MRP: multidrug resistance protein
NCBI: National Center for Biotechnology Information
Neh: NRF2-ECH homology
Nfe2l2: nuclear factor (erythroid-derived 2)-like 2
Nrf2: NF-E2-related factor 2
NSCLC: nonsmall cell lung cancer
NQO1: NAD(P)H:quinone oxidoreductase 1
OR: odds ratio
PD: Parkinson's disease
PM: particulate matter
ROS: reactive oxygen species
SLE: systemic lupus erythematosus
SNP: single nucleotide polymorphism
SOD: superoxide dismutase
URT: untranslated region.
