# Supplementary material for: Genomic Structure and Variation of Nuclear Factor (Erythroid-Derived 2)-Like 2
Source: Oxid Med Cell Longev. 2013 Jul 10;2013:286524. doi: 10.1155/2013/286524 (PMC3723247; doi:10.1155/2013/286524)
Supplement: Supplementary file 1 — Supplementary Table S1 includes genetic mutations in human NRF2 focus (7 kb upstream included) compiled from public database. Supplementary Table S2 includes genetic mutations in murine NRF2 focus (5 kb upstream and 2 kb downstream included) collected from public database for 17 inbred strains. [file 286524.f1.pdf]

	A	B	C	D	E	F	G
1	**Supplementary Table S1**. Genetic mutations in human *NRF2* .						
2	**ID**	**Map on chromosome 2*/HGVS Name**	**Position at mRNA¶/HGVS Name**	**Regions**	**Summary**	**Minor Allele Frequency (MAF)/Count**	**MAF sources**
3	rs16865105	g.178136629A>C	-	5'flanking	-6770	C=0.1928/421	1000 Genomes
4	rs7557529	g.178135097C>T	-	5'flanking	-5238	C=0.718/252	PubMed
5	rs2886162	g.178133165A>G	-	5'flanking	-3306	T=0.324	PubMed
6	rs6750320	g.178131796C>T	c.-555-1937G>A	5'flanking	-1937	T=0.0302/66	1000 Genomes
7	rs181162518	g.178131774T>C	c.-555-1915A>G	5'flanking	-1915	C=0.0005/1	1000 Genomes
8	rs190044775	g.178131746C>T	c.-555-1887G>A	5'flanking	-1887	T=0.0005/1	1000 Genomes
9	rs185117338	g.178131704A>G	c.-555-1845T>C	5'flanking	-1845	G=0.0005/1	1000 Genomes
10	rs149947189	g.178131697C>T	c.-555-1838G>A	5'flanking	-1838	T=0.0009/2	1000 Genomes
11	rs139771244	g.178131625A>G	c.-555-1766T>C	5'flanking	-1766	G=0.0009/2	1000 Genomes
12	rs6747203	g.178131604C>G	c.-555-1745G>C	5'flanking	-1745	G=0.006/13	1000 Genomes
13	rs193101749	g.178131504T>C	c.-555-1645A>G	5'flanking	-1645	C=0.0046/10	1000 Genomes
14	rs190630762	g.178131495G>C	c.-555-1636C>G	5'flanking	-1636	C=0.0009/2	1000 Genomes
15	rs183764402	g.178131366C>A	c.-555-1507G>T	5'flanking	-1507	A=0.0005/1	1000 Genomes
16	rs191222964	g.178131211G>A	c.-555-1352C>T	5'flanking	-1352	A=0.0009/2	1000 Genomes
17	rs187137522	g.178131165T>G	c.-555-1306A>C	5'flanking	-1306	G=0.0009/2	1000 Genomes
18	rs182620359	g.178131158A>G	c.-555-1299T>C	5'flanking	-1299	G=0.0005/1	1000 Genomes
19	rs4893819 (rs61433302)	g.178131134C>T	c.-555-1275G>A	5'flanking	-1275	C=0.4263/931	1000 Genomes
20	rs191547130	g.178131017C>T	c.-555-1158G>A	5'flanking	-1158	T=0.0005/1	1000 Genomes
21	rs188422217	g.178131003A>G	c.-555-1144T>C	5'flanking	-1144	G=0.0014/3	1000 Genomes
22	rs143047764	g.178130865A>G	c.-555-1006T>C	5'flanking	-1006	G=0.0069/15	1000 Genomes
23	rs74432849	g.178130766C>A	c.-555-907G>T	5'flanking	-907	na	
24	rs11679252	g.178130691C>G	c.-555-832G>C	5'flanking	-832	na	
25	rs12993217	g.178130516A>G	c.-555-657T>C	5'flanking	-657	na	
26	rs115644826	g.178130442T>A	c.-555-583A>T	5'flanking	-583	A=0.0151/33	1000 Genomes
27	rs140803524	g.178130431G>A	c.-555-572C>T	5'flanking	-572	A=0.0046/10	1000 Genomes
28	rs77684420	g.178130427T>C	c.-555-568A>G	5'flanking	-568	C=0.0339/74	1000 Genomes PubMed
29	rs183651094	g.178130336A>T	c.-555-477T>A	5'flanking	-477	T=0.0018/4	1000 Genomes
30	rs35652124§ (rs57695243)	g.178130073T>C	c.-555-214A>G	5'flanking	-214	C=0.3512/767 various	1000 Genomes PubMed
31	rs6706649§	g.178130071C>T	c.-555-212G>A	5'flanking	-212	T=0.078/170 various	1000 Genomes PubMed
32	rs150648896	g.178130047C>G	c.-555-188G>C	5'flanking	-188	G=0.0023/5	1000 Genomes PubMed
33	rs6721961§ (rs117801448)	g.178130037T>C, T>G	c.-555-178A>C, A>G	5'flanking	-178	T=0.150/328 various	1000 Genomes PubMed
34	rs201345604	g.178129924_178129925insG	c.-555-66_-555-65insC	5'flanking	-66_-65	G=0.0179/39	1000 Genomes
35	rs200432479	g.178129741_178129742delAA	c.-438_-437delTT	exon 1 (UTR-5')	mRNA 118_119	-=0.0037/8 -=0.53	1000 Genomes PubMed
36	rs75485459	g.178129608C>A	c.-304G>T	exon 1 (UTR-5')	mRNA 252	na	
37	rs192086766	g.178129466C>T	c.-162G>A	exon 1 (UTR-5')	mRNA 394	T=0.022/48 C=0.145/318	1000 Genomes PubMed
38	-	g.178129442G>A	c.-138C>T	exon 1 (UTR-5')	mRNA 418	A=0.012	PubMed
39	rs71668246	g.178129400delG	c.-96delC	exon 1 (UTR-5')	mRNA 460	na	
40	rs187291840	g.178129399C>T	c.-95G>A	exon 1 (UTR-5')	mRNA 461	T=0.0549/120	1000 Genomes
41	rs143406266	g.178129391_178129393delGGC	c.-89_-87delGCC	exon 1 (UTR-5')	mRNA 467_469	-=0.644/282 - =0.589	PubMed
42	rs112882959	g.178129147T>C	c.45+113A>G	intron 1		na	
43	rs4319966	g.178129002C>A	c.45+258G>T	intron 1		na	
44	rs112246802	g.178128828A>G	c.45+432T>C	intron 1		na	
45	rs17524059	g.178128693A>C	c.45+567T>G	intron 1		C=0.2079/454	1000 Genomes
46	rs111925788	g.178128676G>A	c.45+584C>T	intron 1		na	
47	rs112135794	g.178128624G>C	c.45+636C>G	intron 1		na	
48	rs113034282	g.178128540G>C	c.45+720C>G	intron 1		na	
49	rs17551500	g.178128448C>A	c.45+812G>T	intron 1		A=0.0114/25	1000 Genomes
50	rs17522805	g.178128362 C>G	c.45+898G>C	intron 1		na	
51	rs182395677	g.178128277G>T	c.45+983C>A	intron 1		T=0.0023/5	1000 Genomes
52	rs201289976	g.178128177T>C	c.45+1083A>G	intron 1		na	
53	rs189933272	g.178128120G>C	c.45+1140C>G	intron 1		C=0.0018/4	1000 Genomes
54	rs185170228	g.178128028G>A	c.45+1232C>T	intron 1		A=0.0014/3	1000 Genomes
55	rs181303187	g.178128004T>G	c.45+1256A>C	intron 1		G=0.0009/2	1000 Genomes
56	rs7564947 (rs58679638)	g.178128003T>C	c.45+1257A>G	intron 1		C=0.2312/505	1000 Genomes
57	rs2886161§	g.178127839T>C	c.45+1421A>G	intron 1		C=0.350/765 T=0.878/309	1000 Genomes PubMed
58	rs73979903	g.178127826C>T	c.45+1434G>A	intron 1		T=0.0256/56	1000 Genomes
59	rs141904435	g.178127777C>T	c.45+1483G>A	intron 1		T=0.0005/1	1000 Genomes
60	rs76611439	g.178127494G>A	c.45+1766C>T	intron 1		na	
61	rs201655804	g.178127472_178127473delAT	c.45+1787_45+1788delAT	intron 1		-=0.0293/64	1000 Genomes
62	rs72042416	g.178127468delA	c.45+1792delT	intron 1		na	
63	rs138113967	g.178127468-178127470delAAT	c.45+1790_45+1792delATT	intron 1		na	
64	rs189669687	g.178127289A>C	c.45+1971T>G	intron 1		C=0.0005/1	1000 Genomes
65	rs186972464	g.178127263T>C	c.45+1997A>G	intron 1		C=0.0009/2	1000 Genomes
66	rs116657784	g.178127172A>G	c.45+2088T>C	intron 1		G=0.0023/5	1000 Genomes
67	rs34009885	g.178127089A>G	c.45+2171T>C	intron 1		G=0.3516/768	1000 Genomes

	A	B	C	D	E	F	G
2	**ID**	**Map on chromosome 2*/HGVS Name**	**Position at mRNA¶/HGVS Name**	**Regions**	**Summary**	**Minor Allele Frequency (MAF)/Count**	**MAF sources**
68	rs148128831	g.178127066G>C	c.45+2194C>G	intron 1		C=0.0005/1	1000 Genomes
69	rs34551701 (rs58170107)	g.178127040A>C	c.45+2220T>G	intron 1		C=0.2047/447	1000 Genomes
70	rs143764709	g.178127034T>A	c.45+2226A>T	intron 1		A=0.0014/3	1000 Genomes
71	rs6709422	g.178126817T>A	c.45+2443A>T	intron 1		na	
72	rs181805076	g.178126687T>C	c.45+2573A>G	intron 1		C=0.0005/1	1000 Genomes
73	rs80141132	g.178126676A>G	c.45+2584T>C	intron 1		G=0/0	1000 Genomes
74	rs199784732	g.178126608_178126609insA	c.45+2651_45+2652insT	intron 1		A=0.0092/20	1000 Genomes
75	rs2364723§ (rs56505635,rs5808331)	g.178126546G>C	c.45+2714C>G	intron 1		C=0.352/768<br>C=0.525	1000 Genomes<br>PubMed
76	rs80018561	g.178126316C>A	c.45+2944G>T	intron 1		A=0.0192/42	1000 Genomes
77	rs142240496	g.178126277T>C	c.45+2983A>G	intron 1		C=0.0005/1	1000 Genomes
78	rs190525518	g.178126248C>A	c.45+3012G>T	intron 1		A=0.0018/4	1000 Genomes
79	rs56718550	g.178126127C>T	c.45+3133G>A	intron 1		T=0.1548/336	1000 Genomes
80	rs145225605	g.178126116G>A	c.45+3144C>T	intron 1		A=0.0005/1	1000 Genomes
81	rs77992884	g.178125993G>A	c.45+3267C>T	intron 1		A=0.0252/55	1000 Genomes
82	rs140249633	g.178125952C>A	c.45+3308G>T	intron 1		A=0.0032/7	1000 Genomes
83	rs7596275	g.178125914C>A	c.45+3346G>T	intron 1		na	
84	rs59040355	g.178125913_178125914insAA, insAAA	c.45+3346_45+3347insTT, insTTT	intron 1		na	
85	rs71956253<br>(rs71992218,rs72324788,rs138134697)	g.178125899_178125900insAA, insAAA	45+3360_45+3361insTT, insTTT	intron 1		na	
86	rs71410739	g.178125898_178125899insAA	c.45+3361_45+3362insTT	intron 1		na	
87	rs58493972	g.178125722G>A	c.45+3538C>T	intron 1		A=0.1923/420	1000 Genomes
88	rs184632003	g.178125656T>G	c.45+3604A>C	intron 1		G=0.006/13	1000 Genomes
89	rs7593592	g.178125623G>A	c.45+3639C>T	intron 1		A=0.0169/37	1000 Genomes
90	rs67060362 (rs67060363)	g.178125538_178125539insAACA	c.45+3721_45+3722insTGTT	intron 1		na	
91	rs56339224 (rs58213900)	g.178125537_178125538unsAACA	c.45+3722_45+3723insTGTT	intron 1		na	
92	rs66614960 (rs66614961)	g.178125536_178125537insCAAA	c.45+3723_45+3724insTTTG	intron 1		na	
93	rs147426191	g.178125535_178125536insCAAA	c.45+3724_45+3725insTTTG	intron 1		na	
94	rs76201577	g.178125532_178125533insGGC	c.45+3727_45+3728insGCC	intron 1		na	
95	rs55940464 (suspected)	g.178125528A>G	c.45+3732T>C	intron 1		na	
96	rs192396116	g.178125302G>A	c.45+3958C>T	intron 1		A=0.0005/1<br>A=0.452/988	1000 Genomes<br>PubMed
97	rs188222526	g.178125281T>C	c.45+3979A>G	intron 1		C=0.0009/2	1000 Genomes
98	rs116709891	g.178125225T>C	c.45+4035A>G	intron 1		C=0.0018/4	1000 Genomes
99	rs34468415	g.178125142A>G	c.45+4118T>C	intron 1		G=0.2166/473	1000 Genomes
100	rs140782632	g.178125103A>G	c.45+4157T>C	intron 1		G=0.0073/16	1000 Genomes
101	rs183492729	g.178124868T>C	c.45+4392A>G	intron 1		C=0.0037/8	1000 Genomes
102	rs192793174	g.178124819C>T	c.45+4441G>A	intron 1		T=0.0005/1	1000 Genomes
103	rs2364722 (rs59229886)	g.178124787 A>G	c.45+4473T>C	intron 1		G=0.3516/768	1000 Genomes<br>PubMed
104	rs79632183	g.178124660A>C	c.46+4600T>G	intron 1		na	
105	rs78764917	g.178124659C>A	c.45+4601G>T	intron 1		na	
106	rs188213163	g.178124639G>A	c.45+4621C>T	intron 1		A=0.0069/15	1000 Genomes
107	rs150874658	g.178124638C>T	c.45+4622G>A	intron 1		T=0.0078/17	1000 Genomes
108	rs183571722	g.178124515C>A	c.45+4745G>T	intron 1		A=0.0009/2	1000 Genomes
109	rs112899296	g.178124508A>G	c.45+4752T>C	intron 1		G=0.0073/16	1000 Genomes
110	rs12185576	g.178124386C>T	c.45+4874G>A	intron 1		T=0.3526/770	1000 Genomes
111	sr200287267	g.178124075delG	c.45+5185delC	intron 1		-=0.0069/15	1000 Genomes
112	rs200672646	g.17812403delA	c.45+5226delT	intron 1		na	
113	rs112167582	g.178124029C>A	c.45+5237G>T	intron 1		A=0.0275/60	1000 Genomes
114	rs57136150	g.178123987A>G	c.45+5273T>C	intron 1		na	
115	rs193061352	g.178123955G>A	c.45+5305C>T	intron 1		A=0.0005/1<br>T=0.077/168	1000 Genomes<br>PubMed
116	rs141429955	g.178123896G>A	c.45+5364C>T	intron 1		A=0.0082/18	1000 Genomes
117	rs147860035	g.178123867T>C	c.45+5393A>G	intron 1		C=0.0087/19	1000 Genomes
118	rs111507120	g.178123805A>C	c.45+5455T>G	intron 1		na	
119	rs2364721	g.178123692T>C	c.45+5568A>G	intron 1		C=0.0252/55	1000 Genomes
120	rs112641523	g.178123579C>T	c.45+5681G>A	intron 1		na	
121	rs144599315	g.178123454T>A	c.45+5806A>T	intron 1		A=0.0018/4	1000 Genomes
122	rs115730108	g.178123384T>C	c.45+5876A>G	intron 1		C=0.0069/15	1000 Genomes
123	rs115138774	g.178123361A>G	c.45+5899T>C	intron 1		G=0.0009/2	1000 Genomes
124	rs187416832	g.178123177G>A	c.45+6083C>T	intron 1		A=0.0014/3	1000 Genomes
125	rs72946143	g.178123129T>C	c.45+6131A>G	intron 1		C=0.027/59	1000 Genomes
126	rs183440350	g.178123090T>G	c.45+6170A>C	intron 1		G=0.0069/15	1000 Genomes
127	rs190871210	g.178123071T>A	c.45+6189A>T	intron 1		A=0.0005/1	1000 Genomes
128	rs140687001	g.178123011T>C	c.45+6249A>G	intron 1		C=0.0005/1	1000 Genomes
129	rs144243231	g.178123010A>G	c.45+6250T>C	intron 1		G=0.0005/1	1000 Genomes
130	rs112008835	g.178123002G>A	c.45+6258C>T	intron 1		A=0.0023/5	1000 Genomes
131	rs139330296	g.178122955A>G	c.45+6305T>C	intron 1		G=0.0009/2	1000 Genomes
132	rs35959128	g.178122742G>T	c.45+6518C>A	intron 1		na	
133	rs62173695	g.178122727G>A	c.45+6533C>T	intron 1		A=0.217/474	1000 Genomes
134	rs142177417	g.178122717C>A	c.45+6543G>T	intron 1		A=0.0014/3	1000 Genomes

	A	B	C	D	E	F	G
2	**ID**	**Map on chromosome 2*/HGVS Name**	**Position at mRNA¶/HGVS Name**	**Regions**	**Summary**	**Minor Allele Frequency (MAF)/Count**	**MAF sources**
135	rs116498373	g.178122716C>T	c.45+6544G>A	intron 1		T=0.0009/2	1000 Genomes
136	rs59896080	g.178122667T>C	c.45+6593A>G	intron 1		C=0.0266/58	1000 Genomes
137	rs116388253	g.178122635A>G	c.45+6625T>C	intron 1		G=0.0009/2	1000 Genomes
138	rs185321657	g.178122515G>A	c.45+6745C>T	intron 1		A=0.0009/2	1000 Genomes
139	rs182287178	g.178122467C>A	c.45+6793G>T	intron 1		A=0.0009/2	1000 Genomes
140	rs139003635	g.178122449C>T	c.45+6811G>A	intron 1		T=0.0009/2	1000 Genomes
141	rs191346179	g.178122404T>C	c.45+6856A>G	intron 1		C=0.0005/<br>T=0.257/562	1000 Genomes<br>PubMed
142	rs12471433	g.178122365A>C	c.45+6895T>G	intron 1		C=0.348/760	1000 Genomes
143	rs116627712	g.178122330C>T	c.45+6930G>A	intron 1		T=0.0023/5	1000 Genomes
144	rs146144436	g.178122328T>C	c.45+6932A>G	intron 1		C=0.0018/4	1000 Genomes
145	rs12463389	g.178122301C>G	c.45+6965G>C	intron 1		G=0.0124/27	1000 Genomes
146	rs186455300	g.178122237G>C	c.45+7023C>G	intron 1		C=0.0005/1	1000 Genomes
147	rs148182648	g.178122204T>G	c.45+7056A>C	intron 1		G=0.0005/1	1000 Genomes
148	rs181844919	g.178122193C>T	c.45+7067G>A	intron 1		T=0.0009/2	1000 Genomes
149	rs143029845	g.178122141T>C	c.45+7119A>G	intron 1		C=0.0023/5	1000 Genomes
150	rs190332705	g.178122082A>T	c.45+7178T>A	intron 1		T=0.0005/1	1000 Genomes
151	rs75725292	g.178121960C>G	c.45+7300G>C	intron 1		G=0.0005/1	1000 Genomes
152	rs185736510	g.178121903G>A	c.45+7357>T	intron 1		A=0.0009/2	1000 Genomes
153	rs149058464	g.178121881T>G	c.45+7379A>C	intron 1		G=0.0023/5	1000 Genomes
154	rs146980194	g.178121707G>A	c.45+7553C>T	intron 1		A=0.0082/18	1000 Genomes
155	rs73976300	g.178121653C>T	c.45+7607G>A	intron 1		T=0.2015/440	1000 Genomes
156	rs35284526 (rs62173694)	g.178121524C>A	c.45+7736G>T	intron 1		A=0.228/498	1000 Genomes
157	rs181909458	g.178121493C>T	c.45+7767G>A	intron 1		T=0.0009/2	1000 Genomes
158	rs202099449	g.178121470-17812147insC	c.-45+7789_45+7790insG	intron 1		C=0.0069/15	1000 Genomes
159	rs189154074	g.178121430A>C	c.45+7830T>G	intron 1		C=0.0009/2	1000 Genomes
160	rs141366133	g.178121377A>T	c.45+7883T>A	intron 1		T=0.0023/5	1000 Genomes
161	rs201153247	g.178121249G>A	c.45+8017C>T	intron 1		na	
162	rs60758950	g.178121228delA	c.45+8018delT	intron 1		na	
163	rs35073132 (rs71007983,rs150436082)	g.178121228_178121229insA, insAA	c.45+8031_45+8032insT, insTT	intron 1		na	
164	rs13005431§ (rs17647486,rs59481023)	g.178121112T>C	c.45+8148A>G	intron 1		C=0.2679/585	1000 Genomes
165	rs77123162	g.178121031A>G	c45+8229T>C	intron 1		na	
166	rs13028464 (rs59757069)	g.178121005C>T	c.45+8255G>A	intron 1		T=0.2344/512	1000 Genomes
167	rs183754294	g.178120975A>G	c.45+8285T>C	intron 1		G=0.0005/1	1000 Genomes
168	rs77164235	g.178120972A>G	c.45+8288T>C	intron 1		G=0.0018/4	1000 Genomes
169	rs61130794	g.178120681G>T	c.45+8579C>A	intron 1		T=0.0073/16	1000 Genomes
170	rs79705589	g.178120666G>T	c.45+8594C>A	intron 1		T=0.0147/32	1000 Genomes
171	rs75868895	g.178120587C>T	c.45+8673G>A	intron 1		T=0.0082/18	1000 Genomes
172	rs147830306	g.178120425T>C	c.45+8835A>G	intron 1		C=0.0069/15	1000 Genomes
173	rs10188193 (rs58739067)	g.178120391T>C	c.45+8869A>G	intron 1		T=0.1474/322	1000 Genomes
174	rs10188107 (rs56820148)	g.178120312T>G	c.45+8948A>C	intron 1		T=0.1474/322	1000 Genomes
175	rs180825596	g.178120188T>C	c.45+9072A>G	intron 1		C=0.0005/1	1000 Genomes
176	rs1444483619	g.178120104T>C	c.45+9156A>G	intron 1		C=0.0005/1	1000 Genomes
177	rs73031369	g.178120037C>A	c.45+9223G>T	intron 1		A=0.0073/16	1000 Genomes
178	rs15035228	g.178119953G>A	c.45+9307C>T	intron 1		A=0.0023/5	1000 Genomes
179	rs142832274	g.178119904G>C	c.45+9356C>G	intron 1		C=0.0005/1	1000 Genomes
180	rs79225171	g.178119883T>C	c.45+9377A>G	intron 1		C=0.0073/16	1000 Genomes
181	rs80240774	g.178119836G>A	c.45+9424C>T	intron 1		A=0.0005/1	1000 Genomes
182	rs148529343	g.178119818C>T	c.45+9442G>A	intron 1		T=0.0069/15	1000 Genomes
183	rs58549185	g.178119733A>G	c.45+9527T>C	intron 1		G=0.0073/16	1000 Genomes
184	rs144747513	g.178119713T>C	c.45+9547A>G	intron 1		C=0.0023/5	1000 Genomes
185	rs189688195	g.178119676T>C	c.45+9584A>G	intron 1		C=0.0014/3	1000 Genomes
186	rs143718832	g.178119567T>C	c.45+9693A>G	intron 1		C=0.0009/2	1000 Genomes
187	rs11892777 (rs61284966)	g.178119515T>C	c.45+9753A>G	intron 1		C=0.0183/40	1000 Genomes
188	rs140110914	g.178119366C>T	c.45+9894G>A	intron 1		T=0.0069/15	1000 Genomes
189	rs111914065	g.178119340C>T	c.45+9920G>A	intron 1		T=0.0078/17	1000 Genomes
190	rs146125836	g.178119332T>A	c.45+9928A>T	intron 1		A=0.0037/8	1000 Genomes
191	rs115740359	g.178119313A>T	c.45+9947T>A	intron 1		T=0.0005/1	1000 Genomes
192	rs10497511 (rs59034007)	g.178119296G>A	c.45+9964C>T	intron 1		G=0.1479/323	1000 Genomes
193	rs36030784 (rs62173693)	g.178119204A>C	c.45+10056T>G	intron 1		C=0.2285/499	1000 Genomes
194	rs184967958	g.178119169G>C	c.45+10091C>G	intron 1		C=0.0005/1	1000 Genomes
195	rs74762867	g.178119125A>C	c.45+10135T>G	intron 1		na	
196	rs11886935	g.178119040G>C	c.45+10228C>G	intron 1		A=0.0014/3	1000 Genomes
197	rs143013625	g.178119021T>G	c.45+10239A>C	intron 1		G=0.0055/12	1000 Genomes
198	rs13001694§	g.178118990A>G	c.45+10270T>C	intron 1		G=0.271/591<br>G=0.401/1578	1000 Genomes<br>PubMed
199	rs192577308	g.178118878G>A	c.45+10382C>T	intron 1		A=0.0014/3<br>T=0.2569/562	1000 Genomes<br>PubMed
200	rs187543639	g.178118702G>A	c.45+10558C>T	intron 1		A=0.0027/6	1000 Genomes
201	rs2001297	g.178118550C>A	c.45+10710G>T	intron 1		C=0.1937/423	1000 Genomes
202	rs6708603	g.178118544A>T	c.45+10716T>A	intron 1		na	

	A	B	C	D	E	F	G
2	**ID**	**Map on chromosome 2*/HGVS Name**	**Position at mRNA¶/HGVS Name**	**Regions**	**Summary**	**Minor Allele Frequency (MAF)/Count**	**MAF sources**
203	rs34278329	g.178118438_178118439insT	c.45+10821_45+10822insA	intron 1		na	
204	rs116539895	g.178118399A>G	c.45+10861T>C	intron 1		G=0.0197/43	1000 Genomes
205	rs34820876§	g.178118341A>G	c.45+10919T>C	intron 1		G=0.011/25	1000 Genomes
206	rs73031364	g.178118298C>A	c.45+10962G>T	intron 1		A=0.0078/17	1000 Genomes
207	rs150507179	g.178118287C>T	c.45+10973G>A	intron 1		T=0.0005/1	1000 Genomes
208	rs9282797	g.178118218C>T	c.45+11050G>A	intron 1		na	
209	rs1806649§ (rs58745895)	g.178118152C>T	c.45+11108G>A	intron 1		T=0.139/303 various	1000 Genomes PubMed
210	rs141266257	g.178118128_178118129insCTTTA	c.45+11131_45+11132insTAAAG	intron 1		CTTTA=0.0073/ 16	1000 Genomes
211	rs36031254	g.178118020_178118021insC	c.45+11239_45+11240insG	intron 1		na	
212	rs9282798	g.178118005A>G	c.45+11255T>C	intron 1		G=0.006/13	1000 Genomes
213	rs116768505	g.178117926C>A	c.45+11334G>T	intron 1		A=0.0005/1	1000 Genomes
214	rs148002504	g.178117902A>T	c.45+11358T>A	intron 1		T=0.0027/6	1000 Genomes
215	rs4243387§ (rs60038464)	g.178117765C>T	c.45+11495G>A	intron 1		C=0.272/594 T=0.091/425	1000 Genomes PubMed
216	rs146950673	g.178117727T>C	c.45+11533A>G	intron 1		C=0.0005/1	1000 Genomes
217	rs142761256	g.178117669T>G	c.45+11591A>C	intron 1		G=0.0037/8	1000 Genomes
218	rs113166073	g.178117645G>A	c.45+11615C>T	intron 1		A=0.0147/32	1000 Genomes
219	rs184355665	g.178117519C>T	c.45+11741G>A	intron 1		T=0.0005/1	1000 Genomes
220	rs192689231	g.178117410G>A	c.45+11850C>T	intron 1		A=0.0009/2 C=0.194/424	1000 Genomes PubMed
221	rs117045674	g.178117408C>T	c.45+11852G>A	intron 1		T=0.006/13	1000 Genomes
222	rs200763783	g.178117391T>G	c.45+11869A>C	intron 1		na	
223	rs35252336	g.178117391_178117392insG	c.45+11868_45+11869insC	intron 1		na	
224	rs2886160 (rs60051182,rs67592643,rs79053437)	g.178117390T>G, delT	c.45+11870A>C, delA	intron 1		na	
225	rs201359924	g.178117390_178117391insG	c.45+11869_45+11870insC	intron 1		na	
226	rs61040831	g.178117389T>C	c.45+11871A>G	intron 1		na	
227	rs66526442	g.178117389_178117390insC	c.45+11870_45+11871insG	intron 1		na	
228	rs56307341 (rs58616691,rs147659763,rs14772300 7)	g.178117388_178117389insC	c.45+11871_45+11872insG	intron 1		na	
229	rs187858565	g.178117355C>T	c.45+11905G>A	intron 1		T=0.0005/1	1000 Genomes
230	rs138787742	g.178117314T>C	c.45+11946A>G	intron 1		C=0.0009/2	1000 Genomes
231	rs182714514	g.178117139T>C	c.45+12121A>G	intron 1		C=0.0009/2	1000 Genomes
232	rs146047420	g.178117117T>C	c.45+12143A>G	intron 1		C=0.0014/3	1000 Genomes
233	rs190952229	g.178117075G>A	c.45+12185C>T	intron 1		A=0.0009/2	1000 Genomes
234	rs149108953	g.178116992G>A	c.45+12268C>T	intron 1		A=0.0014/3	1000 Genomes
235	rs145124788	g.178116977C>T	c.45+12283G>A	intron 1		T=0.0005/1	1000 Genomes
236	rs115767871	g.178116972T>C	c.45+12288A>G	intron 1		C=0.0005/1	1000 Genomes
237	rs72361960	g.178116750_178116751insT	c.45+12509_45+12510insA	intron 1		na	
238	rs33982505	g.178116746_178116747insA, insAA	c.45+12513_45+12514insT, insTT	intron 1		na	
239	rs3082509 (rs60006121)	g.178116744_178116745insA, insAA	c.45+12514_45+12515insT, insTT	intron 1		na	
240	rs200171393	g.178116736_178116737insCAC	c.45+12523_45_12524insGTG	intron 1		na	
241	rs72157284 (rs72204928,rs140495787,rs15099404 7)	g.178116729_178116730insAA	c.45+12530_45+12531insTT	intron 1		na	
242	rs150396319	g.178116628G>A	c.45+12632C>T	intron 1		A=0.0284/62	1000 Genomes
243	rs140431489	g.178116591A>G	c.45+12669T>C	intron 1		G=0,011/24	1000 Genomes
244	rs187515930	g.178116531A>C	c.45+12729T>G	intron 1		C=0.0027/6	1000 Genomes
245	rs183339181	g.178116529C>T	c.45+12731G>A	intron 1		T=0.0005/1	1000 Genomes
246	rs117263449	g.178116459C>T	c.45+12801G>A	intron 1		T=0.0215/47	1000 Genomes
247	rs189958480	g.178116328G>A	c.45+12932C>T	intron 1		A=0.0005/1	1000 Genomes
248	rs185889362	g.178116284T>C	c.45+12976A>G	intron 1		C=0.0064/14	1000 Genomes
249	rs72944931	g.178116262A>G	c.45+12998T>C	intron 1		G=0.0114/25	1000 Genomes
250	rs113118970	g.178116158A>G	c.45+13102T>C	intron 1		G=0.0105/23	1000 Genomes
251	rs35673620	g.178115961_178115962insA	c.45+13298_45+13299insT	intron 1		na	
252	rs181502240	g.178115801T>C	c.45+13459A>G	intron 1		C=0.0005/1	1000 Genomes
253	rs143650089	g.178115769A>G	c.45+13491T>C	intron 1		G=0.0037/8	1000 Genomes
254	rs191012195	g.178115764C>G	c.45+13496G>C	intron 1		G=0.014/3	1000 Genomes
255	rs34658646	g.178115618_178115619insG	c.45+13651_45+13652insC	intron 1		na	
256	rs186163066	g.178115610G>A	c.45+13650C>T	intron 1		A=0.0005/1	1000 Genomes
257	rs116239726	g.178115553G>T	c.45+13707C>A	intron 1		T=0.0133/29	1000 Genomes
258	rs181980037	g.178115440A>G	c.45+13820T>C	intron 1		G=0.0009/2	1000 Genomes
259	rs189513762	g.178115356G>A	c.45+13904C>T	intron 1		A=0.0005/1	1000 Genomes
260	rs79766920	g.178115351G>A	c.45+13909C>T	intron 1		A=0.0201/44	1000 Genomes
261	rs140078582	g.178115016A>C	c.45+14244T>G	intron 1		C=0.0027/6	1000 Genomes
262	rs146606218	g.178114972G>A	c.45+14288C>T	intron 1		A=0.0027/6	1000 Genomes
263	rs184136380	g.178114810T>C	c.45+14450A>G	intron 1		C=0.0005/1	1000 Genomes
264	rs142852961	g.178114784_178114785insT	c.45+14475_45+14476insA	intron 1		na	
265	rs181379009	g.178114761A>T	c.45+14499T>A	intron 1		T=0.0009/2	1000 Genomes
266	rs188661334	g.178114750T>G	c.45+14510A>C	intron 1		G=0.0005/1	1000 Genomes
267	rs78372947	g.178114740C>G	c.45+14520G>C	intron 1		G=0.0183/40	1000 Genomes

	A	B	C	D	E	F	G
2	**ID**	**Map on chromosome 2*/HGVS Name**	**Position at mRNA¶/HGVS Name**	**Regions**	**Summary**	**Minor Allele Frequency (MAF)/Count**	**MAF sources**
268	rs111421909	g.178114726C>T	c.45+14534G>A	intron 1		na	
269	rs184693304	g.178114711G>A	c.45+14549C>T	intron 1		A=0.0005/1	1000 Genomes
270	rs10930781 (rs58817956,rs59650324)	g.178114632A>G	c.45+14628T>C	intron 1		A=0.1456/318	1000 Genomes
271	rs142945272	g.178114528A>G	c.45+14732T>C	intron 1		G=0.0096/21	1000 Genomes
272	rs201025738	g.178114350delT	c.45+14910delA	intron 1		-=0.0032/7	1000 Genomes
273	rs192095254	g.178114281T>C	c.45+14979A>G	intron 1		C=0.0005/1	1000 Genomes
274	rs139805097	g.178114165T>A	c.45+15095A>T	intron 1		na	
275	rs75255145	g.178113977delA,delAinsATAAATAAATAA	c.46-14978delT, delTinsTTATTTATTTAT	intron 1		na	
276	rs71007982	g.178113977_178113978insATAA	c.46-14979_46-14978insTTAT	intron 1		na	
277	rs140315872	g.178113949_178113950insAATA	c.46-14951_46-14950insTATT	intron 1		na	
278	rs143555304	g.178113947_178113948insTAAA	c.46-14949_46-14948insTTTA	intron 1		na	
279	rs143999145	g.178113946_178113957delATAAATAAATAA	c.46-14920_46-14905delTTATTTATTTAT	intron 1		na	
280	rs66799448 (rs66799449,rs72271571)	g.178113946_178113947insATAA	c.46-14948_46-14947insTTAT	intron 1		na	
281	rs76027118	g.178113945delC, delCinsCATAAATAAATAA	c.46-14946delG, delGinsTTATTTATTTATG	intron 1		na	
282	rs145023006	g.178113945_178113946insATAA	c.46-14947_46-14946insTTAT	intron 1		na	
283	rs188009045	g.178113894C>T	c.46-14895G>A	intron 1		T=0.0005/1	1000 Genomes
284	rs185778554	g.178113823C>T	c.46-14824G>A	intron 1		T=0.0018/4	1000 Genomes
285	rs13422804	g.178113818C>T	c.46-14777G>A	intron 1		T=0.0041/9	1000 Genomes
286	rs78907891	g.178113798A>T	c.46-14757T>A	intron 1		na	
287	rs73031362	g.178113650C>T	c.46-14609G>A	intron 1		T=0.0082/18	1000 Genomes
288	rs111914348	g.178113625C>A	c.46-14584G>T	intron 1		na	
289	rs149437504	g.178113544A>G	c.46-14545T>C	intron 1		G=0.0078/17	1000 Genomes
290	rs1962142§ (rs58448508)	g.178113484A>G	c.46-14485T>C	intron 1		A=0.127/278<br>A=0.082	1000 Genomes<br>PubMed
291	rs11690278	g.178113415G>A	c.46-14374C>T	intron 1		A=0.0169/37	1000 Genomes
292	rs187910577	g.178113379C>T	c.46-14380G>A	intron 1		T=0.0023/5	1000 Genomes
293	rs6732277	g.178113284T>C	c.46-14243A>G	intron 1		C=0.0357/78	1000 Genomes
294	rs147355361	g.178113247C>G	c.46-14248G>C	intron 1		G=0.0032/7	1000 Genomes
295	rs182370982	g.178113204C>G	c.46-14205G>C	intron 1		G=0.0027/6	1000 Genomes
296	rs112353264	g.178113199G>A	c.46-14158C>T	intron 1		A=0.005/11	1000 Genomes
297	rs190302019	g.178113048A>G	c.46-14049T>C	intron 1		G=0.0023/5	1000 Genomes
298	rs186938958	g.178113043C>G	c.46-14044G>C	intron 1		G=0.0005/1	1000 Genomes
299	rs138763384	g.178113018T>C	c.46-14019A>G	intron 1		C=0.0005/1	1000 Genomes
300	rs182971839	g.178112950C>T	c.46-13951G>A	intron 1		T=0.0009/2	1000 Genomes
301	rs190576796	g.178112937G>A	c.46-13938C>T	intron 1		A=0.0014/3	1000 Genomes
302	Rs186597219	g.178112841C>G	c.-3-13842G>C	intron 1		G=0.0005/1	1000 Genomes
303	rs75202993	g.178112725G>A	c.46-13684C>T	intron 1		A=0.0169/37	1000 Genomes
304	rs114146136	g.178112722A>G	c.46-13681T>C	intron 1		G=0.0133/29	1000 Genomes
305	rs2272529	g.178112710G>A	c.46-13711C>T	intron 1		A=0.0105/23	1000 Genomes
306	rs115116838	g.178112611C>T	c.46-13612G>A	intron 1		T=0.0133/29	1000 Genomes
307	rs111510170	g.178112592C>T	c.46-13593G>A	intron 1		na	
308	rs77635085	g.178112455A>G	c.46-13456T>C	intron 1		G=0.0156/34	1000 Genomes
309	rs113754574	g.178112431G>A	c.46-13432C>T	intron 1		na	
310	rs57701650	g.178112413_178112414insA	c.46-13415_46-13414insT	intron 1		na	
311	rs182424203	g.178112395A>G	c.46-13396T>C	intron 1		G=0.0032/7	1000 Genomes
312	rs79008407	g.178112370A>C	c.46-13371T>G	intron 1		na	
313	rs112007423	g.178112361T>C	c.46-13362A>G	intron 1		C=0.0073/16	1000 Genomes
314	rs191798810	g.178112344G>A	c.46-13345C>T	intron 1		A=0.0027/16<br>G=0.352/771	1000 Genomes<br>PubMed
315	rs111436754	g.178112304G>A	c.46-13305C>T	intron 1		A=0.0183/40	1000 Genomes
316	rs10195986	g.178112271C>T	c.46-13272G>A	intron 1		na	
317	rs186271356	g.178112242A>T	c.46-13243T>A	intron 1		T=0.0005/1	1000 Genomes
318	rs143584629	g.178112237A>C	c.46-13238T>G	intron 1		C=0.0005/1	1000 Genomes
319	rs180826227	g.178112169C>T	c.46-13170G>A	intron 1		T=0.005/11	1000 Genomes
320	rs112025320	g.178112111C>T	c.46-13112G>A	intron 1		T=0.0087/19	1000 Genomes
321	rs117830843	g.178112093A>G	c.46-13094T>C	intron 1		G=0.0018/4	1000 Genomes
322	rs188812542	g.178111750A>T	c.46-12751T>A	intron 1		T=0.0005/1	1000 Genomes
323	rs185623828	g.178111698C>A	c.46-12699G>T	intron 1		A=0/0	1000 Genomes
324	rs72355286	g.178111503delT, delTinsTTTTTT	c.46-12504delA, delAinsAAAAAA	intron 1		na	
325	rs56311819 (rs57305123,rs146762456)	g.178111503_178111504insT	c.46-12505_46-12504insA	intron 1		na	
326	rs76882261	g.178111336delC, delCinsCT	c.46-12337delG, delGinsAG	intron 1		na	
327	rs11686945 (suspected)	g.178111336C>T	c.46-12337G>A	intron 1		na	
328	rs181165445	g.178111250A>G	c.46-12251T>C	intron 1		G=0.0005/1	1000 Genomes
329	rs189142028	g.178111214T>C	c.46-12215A>G	intron 1		C=0.0005/1	1000 Genomes
330	rs115141505	g.178111212C>A	c.46-12213G>T	intron 1		A=0.0133/29	1000 Genomes
331	rs148905429	g.178111131A>G	c.46-12132T>C	intron 1		G=0.0018/4	1000 Genomes
332	rs185016960	g.178111020G>A	c.46-12021C>T	intron 1		A=0.0009/2	1000 Genomes
333	rs115761043	g.178110967A>T	c.46-11968T>A	intron 1		T=0.0169/37	1000 Genomes
334	rs6708442	g.178110902G>A	c.46-11903C>T	intron 1		A=0.0018/4	1000 Genomes

	A	B	C	D	E	F	G
2	**ID**	**Map on chromosome 2*/HGVS Name**	**Position at mRNA¶/HGVS Name**	**Regions**	**Summary**	**Minor Allele Frequency (MAF)/Count**	**MAF sources**
335	rs145026902	g.178110882C>A	c.46-11883G>T	intron 1		A=0.0005/1	1000 Genomes
336	rs14293347	g.178110789A>T	c.46-11790T>A	intron 1		T=0.0014/3	1000 Genomes
337	rs111748124	g.178110781T>A	c.46-11782A>T	intron 1		na	
338	rs146588063	g.178110773C>G	c.46-11774G>C	intron 1		G=0.0014/3	1000 Genomes
339	rs150030372	g.178110675G>C	c.46-11676C>G	intron 1		C=0.0014/3	1000 Genomes
340	rs139344881	g.178110664G>A	c.46-11665C>T	intron 1		A=0.0005/1	1000 Genomes
341	rs193259326	g.178110651T>G	c.46-11652A>C	intron 1		G=0.0018/4<br>T=0.1495/327	1000 Genomes<br>PubMed
342	rs151304786	g.178110643T>G	c.46-11644A>C	intron 1		G=0.0014/3	1000 Genomes
343	rs190382043	g.178110527G>A	c.46-11528C>T	intron 1		A=0.0009/2	1000 Genomes
344	rs75707887	g.178110355T>G	c.46-11356A>C	intron 1		na	
345	rs80349357	g.178110344T>C	c.46-11345A>G	intron 1		na	
346	rs76185529	g.178110343C>T	c.46-11344G>A	intron 1		na	
347	rs112067562	g.178110342_178110343insT	c.46-11344_46-11343insA	intron 1		na	
348	rs117962208	g.178110283G>T	c.46-11284C>A	intron 1		T=0.0133/29	1000 Genomes
349	rs14026669	g.178110124C>T	c.46-11125G>A	intron 1		T=0.0032/7	1000 Genomes
350	rs184607299	g.178110003C>T	c.46-11004G>A	intron 1		T=0.0005/1	1000 Genomes
351	rs13402068 (rs58997687)	g.178109961A>G	c.46-10962T>C	intron 1		G=0.0421/92	1000 Genomes
352	rs147349290	g.178109836G>C	c.46-10837C>G	intron 1		C=0.0101/22	1000 Genomes
353	rs56326022<br>(rs60030129,rs72065105,rs13980925)	g.178109731delA	c.46-10732delT	intron 1		na	
354	rs191181286	g.178109601G>A	c.46-10602C>T	intron 1		A=0.0009/2	1000 Genomes
355	rs142715562	g.178109509C>T	c.46-10510G>A	intron 1		T=0.022/48	1000 Genomes
356	rs116769762	g.178109434A>C	c.46-10435T>G	intron 1		C=0.0082/18	1000 Genomes
357	rs140376711	g.178109418G>A	c.46-10419C>T	intron 1		A=0.0005/1	1000 Genomes
358	rs186556119	g.178109372C>T	c.46-10373G>A	intron 1		T=0.0018/4	1000 Genomes
359	rs147158417	g.178109353C>T	c.46-10354G>A	intron 1		T=0.0014/3	1000 Genomes
360	rs149487711	g.178109095C>T	c.46-10096G>A	intron 1		T=0.0105/23	1000 Genomes
361	rs200836049	g.178109031A>C	c.46-10032T>G	intron 1		na	
362	rs77969647	g.178109024C>A	c.46-10025G>T	intron 1		na	
363	rs182574883	g.178108987G>A	c.46-9988C>T	intron 1		A=0.0027/6	1000 Genomes
364	rs76718685	g.178108879A>T	c.46-9880T>A	intron 1		na	
365	rs75671898	g.178108841A>T	c.46-9842T>A	intron 1		na	
366	rs191513071	g.178108766C>T	c.46-9767G>A	intron 1		T=0.0032/7<br>A=0.452/988	1000 Genomes<br>PubMed
367	rs188666710	g.178108748G>T	c.46-9749C>A	intron 1		T=0.0005/1	1000 Genomes
368	rs183421956	g.178108646G>A	c.46-9647C>T	intron 1		A=0.0009/2	1000 Genomes
369	rs114513152	g.178108565A>G	c.46-9566T>C	intron 1		G=0.0064/14	1000 Genomes
370	rs77941510	g.178108552C>T	c.46-9553G>A	intron 1		T=0.0316/69	1000 Genomes
371	rs138012339	g.178108493A>C	c.46-9494T>G	intron 1		C=0.0105/23	1000 Genomes
372	rs35069293	g.178108371_178108372insC	c.46-9373_46-9372insG	intron 1		na	
373	rs200105240	g.178108260_178108261insT	c.46-9262_46-9261insA	intron 1		na	
374	rs192468336	g.178108202G>A	c.46-9203C>T	intron 1		A=0.0009/2<br>C=0.1453/318	1000 Genomes<br>PubMed
375	rs116275305	g.178108149C>T	c.46-9150G>A	intron 1		T=0.0009/2	1000 Genomes
376	rs150772414	g.178108061T>A	c.46-9062A>T	intron 1		A=0.0073/16	1000 Genomes
377	rs141344720	g.178107955C>T	c.46-8956G>A	intron 1		T=0.0014/3	1000 Genomes
378	rs138781057	g.178107942T>G	c.46-8943A>C	intron 1		G=0.0082/18	1000 Genomes
379	rs181023734	g.178107878T>C	c.46-8879A>G	intron 1		na	
380	rs144971306	g.178107794C>T	c.46-8795G>A	intron 1		T=0.0005/1	1000 Genomes
381	rs141247651	g.178107718G>A	c.46-8719C>T	intron 1		A=0.0032/7	1000 Genomes
382	rs200970759	g.178107648_178107649insT	c.46-8650_46-8649insA	intron 1		na	
383	rs187134041	g.178107553C>T	c.46-8554G>A	intron 1		T=0.0005/1	1000 Genomes
384	rs138239588	g.178107503C>T	c.46-8504G>A	intron 1		T=0.0009/2	1000 Genomes
385	rs116817143	g.178107277A>G	c.46-8278T>C	intron 1		G=0.0009/2	1000 Genomes
386	rs182398641	g.178107256G>T	c,46-8257C>A	intron 1		T=0.0005/1	1000 Genomes
387	rs189940034	g.178107203T>A	c.46-8204A>T	intron 1		A=0.0005/1	1000 Genomes
388	rs185180616	g.178107167G>T	c.46-8168C>A	intron 1		T=0.0005/1	1000 Genomes
389	rs143692702	g.178107161A>G	c.46-8162T>C	intron 1		G=0.0046/10	1000 Genomes
390	rs181313752	g.178107122T>C	c.46-8123A>G	intron 1		C=0.0005/1	1000 Genomes
391	rs148958299	g.178107102T>A	c.46-8103A>T	intron 1		A=0.0032/7	1000 Genomes
392	rs145790450	g.178107039T>C	c.46-8040A>G	intron 1		C=0.0005/1	1000 Genomes
393	rs189392144	g.178107038A>G	c.46-8039T>C	intron 1		G=0.0005/1	1000 Genomes
394	rs149835220	g.178106962G>A	c.46-7963C>T	intron 1		A=0.0027/6	1000 Genomes
395	rs142620253	g.178106904G>A	c.46-7905C>T	intron 1		A=0.0009/2	1000 Genomes
396	rs147752201	g.178106891T>A	c.46-7892A>T	intron 1		A=0.0018/4	1000 Genomes
397	rs75661630	g.178106758A>C	c.46-7759T>G	intron 1		C=0.0069/15	1000 Genomes
398	rs187169453	g.178106736A>G	c.46-7737T>C	intron 1		G=0.0005/1	1000 Genomes
399	rs145683450	g.178106726G>A	c.46-7727C>T	intron 1		A=0.0027/6	1000 Genomes
400	rs181703340	g.178106335C>T	c.46-7336G>A	intron 1		T=0.0014/3	1000 Genomes
401	rs140260993	g.178106313C>A	c.46-7314G>T	intron 1		A=0.0009/2	1000 Genomes
402	rs35849219	g.178105996delC	c.46-6997delG	intron 1		na	

	A	B	C	D	E	F	G
2	**ID**	**Map on chromosome 2*/HGVS Name**	**Position at mRNA¶/HGVS Name**	**Regions**	**Summary**	**Minor Allele Frequency (MAF)/Count**	**MAF sources**
403	rs10803905 (rs60549745,rs12997535)	g.178105996C>G	c.46-6997G>C	intron 1		C=0.4643/1014	1000 Genomes
404	rs190738455	g.178105970A>T	c.46-6971T>A	intron 1		T=0.0009/2	1000 Genomes
405	rs150511755	g.178105846T>C	c.46-6847A>G	intron 1		C=0.0046/10	1000 Genomes
406	rs142538623	g.178105771G>C	c.46-6772C>G	intron 1		C=0.0105/23	1000 Genomes
407	rs117091737	g.178105676A>G	c.46-6677T>C	intron 1		G=0.0119/26	1000 Genomes
408	rs185629218	g.178105597T>C	c.-3-6598A>G	intron 1		C=0.0005/1	1000 Genomes
409	rs148441415	g.178105372G>C	c.46-6373C>G	intron 1		C=0.0023/5	1000 Genomes
410	rs144543556	g.178105289A>G	c.46-6290T>C	intron 1		G=0.0009/2	1000 Genomes
411	rs192354298	g.178105169C>T	c.46-6170G>A	intron 1		T=0.0009/2<br>A=0.128/280	1000 Genomes<br>PubMed
412	rs12990527 (rs2364720,rs58288492)	g.178105144A>G	c.46-6145T>C	intron 1		A=0.1772/387	1000 Genomes
413	rs188188565	g.178105039G>T	c.46-6040C>A	intron 1		T=0.0023/5	1000 Genomes
414	rs183460473	g.1781049996C>A	c.46-5997G>T	intron 1		A=0.0009/2	1000 Genomes
415	rs141783702	g.178104971C>A	c.46-5972G>T	intron 1		A=0.0005/1	1000 Genomes
416	rs191869222	g.178104833G>C	c.46-5834C>G	intron 1		C=0.0005/1<br>C=0.352/771	1000 Genomes<br>PubMed
417	rs3731799	g.178104760A>G	c.46-5761T>C	intron 1		G=0.0023/5	1000 Genomes
418	rs57941264 (rs66484779,rs66709457,rs66709458,rs67641099,rs67641100,rs67641101,rs72411387)	g.178104678delA	c.46-5679delT	intron 1		na	
419	rs74901734	g.178104476delT	c.46-5477delA	intron 1		na	
420	rs112653114	g.178104463C>A	c.46-5464G>T	intron 1		na	
421	rs138747587	g.178104460A>G	c.46-5461T>C	intron 1		G=0.0005/1	1000 Genomes
422	rs113539054	g.178104458C>A	c.46-5459G>T	intron 1		na	
423	rs183803382	g.178104445T>G	c.46-5446A>C	intron 1		G=0.0014/3	1000 Genomes
424	rs192860219	g.178104106T>G	c.46-5107A>C	intron 1		G=0.0018/4	1000 Genomes
425	rs142680183	g.178104094C>G	c.46-5095G>C	intron 1		G=0.0037/8	1000 Genomes
426	rs188555073	g.178103728A>G	c.46-4729T>C	intron 1		G=0.0005/1	1000 Genomes
427	rs200246174	g.178103722_178103723delAC	c.46-4724_46-4723delGT	intron 1		-=0.0119/26	1000 Genomes
428	rs138179678	g.178103617T>C	c.46-4618A>G	intron 1		C=0.0037/8	1000 Genomes
429	rs150404294	g.178103514C>T	c.46-4515G>A	intron 1		T=0.0037/8	1000 Genomes
430	rs183448760	g.178103495G>T	c.46-4496C>A	intron 1		T=0.0005/1	1000 Genomes
431	rs35064812	g.178103392_178103393insG	c.46-4394_46-4393insC	intron 1		na	
432	rs140498002	g.178103361A>G	c.46-4362T>C	intron 1		G=0.0005/1	1000 Genomes
433	rs148333212	g.178103328C>T	c.46-4329G>A	intron 1		T=0.0101/22	1000 Genomes
434	rs6726395§(rs57309289)	g.178103229A>G	c.46-4230T>C	intron 1		A=0.453/989<br>various	1000 Genomes<br>PubMed
435	rs34605869	g.178103185T>C	c.46-4186G>A	intron 1		C=0.0014/3	1000 Genomes
436	rs185331205	g.178103144C>A	c.-3-4145G>T	intron 1		A=0.0055/12	1000 Genomes
437	rs182296139	g.178103138C>T	c.46-4139G>A	intron 1		T=0.0005/1	1000 Genomes
438	rs191597251	g.178103095T>C	c.46-4096A>G	intron 1		C=0.0005/1<br>C=0.272/595	1000 Genomes<br>PubMed
439	rs186253488	g.178103087A>G	c.46-4088T>C	intron 1		G=0.0018/4	1000 Genomes
440	rs181166091	g.178103051T>G	c.46-4052A>C	intron 1		G=0.0005/1	1000 Genomes
441	rs202181500	g.178103008_178103010delCTG	c.46-4011_46-4009delCAG	intron 1		-=0.0114/25	1000 Genomes
442	rs199878061	g.178103004_178103006delTCT	c.46-4007_46-4005delAGA	intron 1		-=0.0114/25	1000 Genomes
443	rs143328561	g.178102970T>C	c.46-3971A>G	intron 1		C=0.0005/1	1000 Genomes
444	rs142506772	g.178102908G>A	c.46-3909C>T	intron 1		A=0.0023/5	1000 Genomes
445	rs75603023	g.178102697G>T	c.46-3698C>A	intron 1		T=0.0169/37	1000 Genomes
446	rs147723007	g.178102678A>G	c.46-3679T>C	intron 1		G=0.0009/2	1000 Genomes
447	rs191636022	g.178102626T>C	c.46-3627A>G	intron 1		C=0.0009/2<br>C=0.351/769	1000 Genomes<br>PubMed
448	rs2364719 (rs13025227)	g.178102607C>T	c.46-3608G>A	intron 1		c=0/0	1000 Genomes
449	rs145616927	g.178102545C>A	c.46-3546G>T	intron 1		A=0.0009/2	1000 Genomes
450	rs141859565	g.178102285C>T	c.46-3286G>A	intron 1		T=0.0174/38	1000 Genomes
451	rs185698792	g.178102276T>A	c.46-3277A>T	intron 1		A=0.0009/2	1000 Genomes
452	rs10706328 (rs35093114,rs200576805)	g.178102256delT	c.46-3257delA	intron 1		na	
453	rs7599604	g.178102203G>A	c.-46-3204C>T	intron 1		na	
454	rs111241652	g.178102171G>C	c.46-3172C>G	intron 1		na	
455	rs71421594	g.178102107A>G	c.46-3108C>T	intron 1		na	
456	rs112551006	g.178102005C>A	c.46-3006G>T	intron 1		na	
457	rs79628357	g.178101950G>A	c.46-2951C>T	intron 1		A=0.0362/79	1000 Genomes
458	rs181884871	g.178101886C>T	c.46-2887G>A	intron 1		T=0.0009/2	1000 Genomes
459	rs189307803	g.178101851G>C	c.46-2852C>G	intron 1		C=0.0005/1	1000 Genomes
460	rs34165498	g.178101845_178101846insC	c.46-2847_46-2846insG	intron 1		na	
461	rs2364718	g.178101776T>C	c.46-2777A>G	intron 1		C=0.0147/32	1000 Genomes
462	rs149889003	g.178101763C>A	c.46-2764G>T	intron 1		A=0.0101/22	1000 Genomes
463	rs78377016	g.178101561G>T	c.46-2562C>A	intron 1		T=0.0284/62	1000 Genomes
464	rs183761430	g.178101327C>A	c.46-2328G>T	intron 1		A=0.0014/3	1000 Genomes
465	rs78272396	g.178101326A>G	c.46-2327T>C	intron 1		G=0.0169/37	1000 Genomes
466	rs2364717 (rs13012776,rs59430539)	g.178101235C>T	c.46-2236G>A	intron 1		C=0.4597/1004	1000 Genomes
467	rs78613948	g.178101214T>C	c.46-2215A>G	intron 1		C=0.027/59	1000 Genomes

	A	B	C	D	E	F	G
2	**ID**	**Map on chromosome 2*/HGVS Name**	**Position at mRNA¶/HGVS Name**	**Regions**	**Summary**	**Minor Allele Frequency (MAF)/Count**	**MAF sources**
468	rs139085223	g.178101192A>G	c.46-2193T>C	intron 1		G=0.0005/1	1000 Genomes
469	rs180783046	g.178101027T>C	c.46-2028A>G	intron 1		C=0.0005/1	1000 Genomes
470	rs61232320	g.178100974G>T	c.46-1975C>A	intron 1		T=0.0014/3	1000 Genomes
471	rs189957813	g.178100916T>G	c.46-1917A>C	intron 1		G=0.0005/1	1000 Genomes
472	rs57557108	g.178100808A>G	c.46-1809T>C	intron 1		G=0.0165/36	1000 Genomes
473	rs34661184	g.178100783_178100784insA	c.46-1785_46-1784insT	intron 1		na	
474	rs184867003	g.178100735G>A	c.46-1736C>T	intron 1		A=0.0009/2	1000 Genomes
475	rs6707261	g.178100543C>G	c.46-1544G>C	intron 1		G+=0.0023/5	1000 Genomes
476	rs2001350§ (rs17515179,rs60883775)	g.178100425C>T	c.46-1426G>A	intron 1		C=0.146/318<br>T (various)	1000 Genomes<br>PubMed
477	rs139900855	g.178100223G>A	c.46-1224C>T	intron 1		A=0.005/1	1000 Genomes
478	rs192846733	g.178100119C>T	c.46-1120G>A	intron 1		T=0.0018/4	1000 Genomes
479	rs80192071	g.178100090A>G	c.46-1091T>C	intron 1		G=0.0069/15	1000 Genomes
480	rs188418193	g.178099986T>A	c.46-987A>T	intron 1		A=0.0009/2	1000 Genomes
481	rs146003121	g.178099915A>G	c.46-916T>C	intron 1		G=0.0101/22	1000 Genomes
482	rs142621111	g.178099897C>T	c.46-898G>A	intron 1		T=0.0165/36	1000 Genomes
483	rs148494821	g.178099775G>A	c.46-776C>T	intron 1		A=0.0069/15	1000 Genomes
484	rs184364316	g.178099759G>A	c.46-760C>T	intron 1		A=0.0005/1	1000 Genomes
485	rs192698275	g.178099700C>T	c.46-701G>A	intron 1		T=0.0005/1<br>C=0.424/927	1000 Genomes<br>PubMed
486	rs144668476	g.178099653G>C	c.46-654C>G	intron 1		C=0.0037/8	1000 Genomes
487	rs34179691	g.178099615_178099616insG	c.46-617_46-616insC	intron 1		na	
488	rs6433654 (rs61582684)	g.178099612G>C, delG	c.46-613C>G, delC	intron 1		na	
489	rs75402256	g.178099612G>C	c.46-613C>G	intron 1		G=0.4469/976	1000 Genomes
490	rs138732412	g.178099552G>C	c.46-553C>G	intron 1		C=0.0014/3	1000 Genomes
491	rs146476022	g.178099530T>A	c.46-531A>T	intron 1		A=0.0027/6	1000 Genomes
492	rs117916319	g.178099463T>C	c.46-464A>G	intron 1		C=0.0101/22	1000 Genomes
493	rs143918323	g.178099438T>C	c.46-439A>G	intron 1		C=0.0005/1	1000 Genomes
494	rs138440396	g.178099333T>C	c.46-334A>G	intron 1		C=0.0037/8	1000 Genomes
495	rs187866790	g.178099260G>A	c.46-261C>T	intron 1		A=0.0005/1	1000 Genomes
496	rs147100319	g.178099185C>A	c.46-186G>T	intron 1		A=0.0005/1	1000 Genomes
497	rs201921480	g.178099003T>C	c.46-4A>G	intron 1		C=0/0	1000 Genomes
498	rs182428269	g.178098918G>A	c.127C>T	exon 2/mRNA 682	Cns (p.Arg43Trp)	A=0.0005/1	1000 Genomes
499	rs35248500	g.178098917C>T	c.128G>A	exon 2/mRNA 683	Cns (p.Arg43Gln)	T=0.006/13	1000 Genomes
500	rs1135118	g.178098831C>T	c.214G>A	exon 2/mRNA 769	Cns (p.Ala72Thr)	na	PubMed
501	rs199691660	g.178098829A>T	c.216T>A	exon 2/mRNA 771	Cs (p.Ala72=)	na	
502	-	g.178098757C>	c.288G>	exon 2/mRNA 831	Cns (p.Thr92Ile)	na	PubMed
503	rs5031039	g.178098750A>G	c.295T>C	exon 2/mRNA 850	Cns (p.Ser99Pro)	G=0	PubMed
504	rs114377436	g.178098722G>A	c.312+11C>T	intron 2		A=0.0069/15	1000 Genomes
505	rs78088214	g.178098679C>T	c.312+54G>A	intron 2		T=0.0238/52	1000 Genomes
506	rs151335115	g.178098660C>T	c.312+73G>A	intron 2		T=0.0101/22	1000 Genomes
507	rs77817284	g.178098565A>C	c.312+168T>G	intron 2		C=0.0009/2	1000 Genomes
508	rs143434001	g.178098562C>G	c.312+171G>C	intron 2		G=0.0005/1	1000 Genomes
509	rs148816783	g.178098502G>T	c.312+231C>A	intron 2		T=0.0101/22	1000 Genomes
510	rs145469719	g.178098463G>A	c.312+270C>T	intron 2		A=0.0005/1	1000 Genomes
511	rs7604429	g.178098454C>G	c.312+279G>C	intron 2		G=0.0014/3	1000 Genomes
512	rs190854564	g.178098430G>T	c.312+303C>A	intron 2		T=0.0018/4	1000 Genomes
513	rs7604306	g.178098310C>T	c.313-243G>A	intron 2		T=0.0018/4	1000 Genomes
514	rs187729729	g.178098092G>A	c.313-25C>T	intron 2		A=0.0027/6	1000 Genomes
515	rs139080704 (rs148365874)	g.178098088delA	c.313-21delT	intron 2		-=0.0765/167	1000 Genomes
516	rs200239262	g.178098017C>G	c.363G>C	exon 3/mRNA 918	Cns (p.Gln121His)	na	
517	rs183034165	g.178098008T>C	c.372G>A	exon 3/mRNA 927	Cs (p.Ala124=)	T=0.0009/2<br>T=0.006	1000 Genomes<br>PubMed
518	rs199970826	g.178097996C>T	c.384G>A	exon 3/mRNA 939	Cs (p.Pro128=)	T=0.0005/1	1000 Genomes
519	rs191231706	g.178097965T>C	c.402+13A>G	intron 3		C=0.0005/1<br>G=0.271/592	1000 Genomes<br>PubMed
520	rs57572550	g.178097792T>C	c.402+186A>G	intron 3		C=0.0092/20	1000 Genomes
521	rs10183914§ (rs58731187,rs61374844)	g.178097666C>T	c.402+312G>A	intron 3		T=0.2564/560<br>T=0.536/188	1000 Genomes<br>PubMed
522	rs146485862	g.178097587C>G	c.403-276G>C	intron 3		G=0.0069/15	1000 Genomes
523	rs115297462	g.178097545G>A	c.403-234C>T	intron 3		A=0.0018/4	1000 Genomes
524	rs200643669	g.178097346C>T	c.403-35G>A	intron 3		T=0.0005/1	1000 Genomes
525	rs200686306	g.178097321A>G	c.403-10T>C	intron 3		G=0.0005/1	1000 Genomes
526	rs201992337	g.178097260C>T	c.454G>A	exon 4/mRNA 1009	Cns (p.Glu152Lys)	na	
527	rs201589693	g.178097251C>T	c.463G>A	exon 4/mRNA 1018	Cns (p.Val155Ile)	T=0.0005/1	1000 Genomes
528	rs35577826	g.178097185A>C	c.529T>G	exon 4/mRNA 1084	Cns (p.Leu177Val)	C=0.0014/3<br>A<0.005	1000 Genomes<br>PubMed
529	rs80155840	g.178097091T>A	c.594+29A>T	intron 4		na	
530	rs75782703	g.178097090A>T	c.594+30T>A	intron 4		na	
531	rs115871724	g.178096839T>G	c.595-103A>C	intron 4		G=0.0064/14	1000 Genomes
532	rs144119724	g.178096837T>A	c.595-101A>T	intron 4		A=0.0018/4	1000 Genomes
533	rs181513314	g.178096710C>T	c.621G>A	exon 5/mRNA1176	Cn (p.Leu207=)	T=0.0005/1	1000 Genomes
534	rs60132461	g.178096675T>C	c.656A>G	exon 5/mRNA 1211	Cns (p.Lys219Arg)	C=0.0018/4	1000 Genomes

	A	B	C	D	E	F	G
2	**ID**	**Map on chromosome 2*/HGVS Name**	**Position at mRNA¶/HGVS Name**	**Regions**	**Summary**	**Minor Allele Frequency (MAF)/Count**	**MAF sources**
535	rs139187151	g.178096634G>A	c.697C>T	exon 5/mRNA 1252	Cns (p.Pro233Ser)	A=0.0005/1<br>A=0.0012	1000 Genomes<br>PubMed
536	rs35557421	g.178096620delT	c.711delA	exon 5/mRNA 1266	Cs (p.Glu237=fs)	na	
537	rs34154613	g.178096529C>T	c.802G>A	exon 5/mRNA 1357	Cns(p.Val268Met)	T=0.0018/4	1000 Genomes
538	rs141363120	g.178096406G>A	c.925C>T	exon 5/mRNA 1480	Cns (p.Leu309Phe)	A=0.0037/8	1000 Genomes
539	rs201661476	g.178096380A>C	c.951T>G	exon 5/mRNA 1506	Cns (p.Ile2317Met)	na	
540	rs199673454	g.178096309T>A	c.1022A>T	exon 5/mRNA 1577	Cns (p.Asp341Val)	A=0.0005/1	1000 Genomes
541	rs35007548	g.178096299G>A	c.1032C>T	exon 5/mRNA 1587	Cs (p.Ser344=)	A=0.0009/2	1000 Genomes
542	rs200209692	g.178096287T>C	c.1044A>G	exon 5/mRNA 1599	Cs (p.Leu348=)	C=0.0005/1	1000 Genomes
543	-	g.178096237C>A	c.1094G>T	exon 5/mRNA 1649	Cns (p.Ser365Ile)	A=0.125/273<br>A=0.006	PubMed
544	rs201214197	g.178096171C>T	c.1160G>A	exon 5/mRNA 1715	Cns (p.Ser387Asn)	T=0.0005/1	1000 Genomes
545	rs200494292	g.178096165T>C	c.1166A>G	exon 5/mRNA 1721	Cns (p.Lys389Arg)	na	
546	rs186171287	g.178096115G>A	c.1216C>T	exon 5/mRNA 1771	Cns (p.Pro406Ser)	A=0.0005/1	1000 Genomes
547	rs182276775	g.178096062C>A	c.1269G>T	exon 5/mRNA 1824	Cns (p.Glu423Asp)	A=0.0005/1	1000 Genomes
548	rs201560221	g.178096048T>C	c.1283A>G	exon 5/mRNA 1838	Cns (p.Lys428Arg)	na	
549	rs189238236	g.178096043A>G	c.1288T>C	exon 5/mRNA 1843	Cs (p.Leu430=)	G=0.0005/1	1000 Genomes
550	rs184287392	g.178096022G>A	c.1309C>T	exon 5/mRNA 1864	Cns (p.Arg437Trp)	A=0.0005/1	1000 Genomes
551	rs201871588	g.178095986G>A	c.1345C>T	exon 5/mRNA 1900	Cns (p.Arg449Cys)	na	
552	rs181294188	g.178095985T>C	c.1346G>A	exon 5/mRNA 1901	Cns (p.Arg449His)	T=0.0009/2	1000 Genomes
553	rs201690466	g.178095973T>A	c.1358A>T	exon 5/mRNA 1913	Cns (p.His453Leu)	A=0.0005/1	1000 Genomes
554	rs1057044 (rs52789869)	g.178095781C>T	c.1550G>A	exon 5/mRNA 2105	Cns (p.Arg517Lys)	na	PubMed
555	rs200750800	g.178095603A>G	c.1728T>C	exon 5/mRNA 2283	Cs (p.Tyr576=)	na	
556	rs200175942	g.178095567A>G	c.1764T>C	exon 5/mRNA 2319	Cs (p.Asp588=)	G=0.0005/1	1000 Genomes
557	rs77547666	g.178095495G>C	c.*18C>G	exon 5 (UTR-3')	mRNA 2391	C=0.0069/15	1000 Genomes
558	rs73031353	g.178095425T>C	c.*88A>G	exon 5 (UTR-3')	mRNA 2461	C=0.0018/4	1000 Genomes
559	rs6759443	g.178095345T>C	c.*168A>G	exon 5 (UTR-3')	mRNA 2541	C=0.0041/9	1000 Genomes
560	rs188674558	g.178095279C>A	c.*234G>T	exon 5 (UTR-3')	mRNA 2607	A=0.0005/1	1000 Genomes
561	rs77685897	g.178095247A>G	c.*266T>C	exon 5 (UTR-3')	mRNA 2639	G=0.0009/2	1000 Genomes
562	rs1057092	g.178095162T>G	c.*351A>C	exon 5 (UTR-3')	mRNA 2724	na	
563	rs3197704	g.178095162T>G	c*351A>C	exon 5 (UTR-3')	mRNA 2724	na	
564	rs184701151	g.178095159T>C	c.*354A>G	exon 5 (UTR-3')	mRNA 2727	C=0.0027/6	1000 Genomes
565	rs11543307	g.178095153A>G	c.*360T>C	exon 5 (UTR-3')	mRNA 2733	na	
566	rs111874043	g.178095146A>G	c.*367T>C	exon 5 (UTR-3')	mRNA 2740	na	
567	rs34012004	g.178095102A>C	c.*411T>G	exon 5 (UTR-3')	mRNA 2784	C=0.071	PubMed
568	rs201481890	g.178095090_178095091insT	c.*422_*423insA	exon 5 (UTR-3')	mRNA 2795-2796	na	
569	rs3082500	g.178095089_178095090delTT, delTTinsT	c.*423_*424delAAinsA	exon 5 (UTR-3')	mRNA 2796-2797	na	
570	rs71792546 (rs71796710)	g.178095079delT	c.*425delA	exon 5 (UTR-3')	mRNA 2798	na	
571	rs1057106	g.178095078A>C, A>T	c.*435T>A, T>G	exon 5 (UTR-3')	mRNA 2808	na	
572	rs34176791	g.178095076A>C	c.*437T>G	exon 5 (UTR-3')	mRNA 2810	C=0.0023/5	1000 Genomes
573	rs35911553	g.178095045C>T	c.*468G>A	exon 5 (UTR-3')	mRNA 2841	T=0.0069/15	1000 Genomes
574	rs144190519	g.178095001delTG	c.*482+30_*482+31delCA	3'flanking		-=0.0238/52	1000 Genomes
575	rs34261474	g.178094967_178094968insC	c.*482+63_*482+64insG	3'flanking		na	
576	rs11370732 (rs144540636)	g.178094964_178094965insC	c.*480+68_*480+69insG	3'flanking		C=0.0114/25	1000 Genomes
577	rs199663976	g.178094874A>T	c.*482+157T>A	3'flanking		na	
578	rs147246052	g.178094764G>A	c.*482+267C>T	3'flanking		A=0.0014/3	1000 Genomes
579	rs80324085	g.178094714G>A	c.*482+317C>T	3'flanking		A=0.0018/4	1000 Genomes
580	rs7603691	g.178094622T>C	c.*482+409A>G	3'flanking		C=0.0018/4	1000 Genomes
581	rs138431904	g.178094611A>G	c.*482+420T>C	3'flanking		G=0.0018/4	1000 Genomes
582	rs114146942	g.178094572G>A	c.*482+459C>T	3'flanking		A=0.0238/52	1000 Genomes
583	rs78677742	g.178094564C>T	c.*482+467G>A	3'flanking		T=0.005/11	1000 Genomes
584	rs2706110	g.178092162T>C		3'flanking		na	PubMed
585	rs13035806	g.178091822G>A		3'flanking		A=0.124/272	1000 Genomes
586	rs2588882	g.178087165G>T		3'flanking		na	PubMed

Supplementary Table S2. Mouse *Nrf2* genetic sequence variation.

ID	Ch2 bp Location	Gene	Distance (gene)	Distance (mRNA)	Region	Alleles (genome)	Consequence	C57BL/6J	129S1/SvImJ	A/J	AKR/J	BALB/cJ	C3H/HeJ	C57BL/6NJ	CAST/EiJ	CBA/J	DBA/2J	FVB/NJ	LP/J	NOD/ShiLtJ	NZO/HlLtJ	PWK/PhJ	SPRET/EiJ	WSB/EiJ
**rs230809422**	75709621	E030042O20Rik	4980	-4980	5'flanking	C/T	NC	T	T	T	T	T	T	T	C	T	T	T	T	T	T	T	T	T
**rs255224698**	75709614	E030042O20Rik	4973	-4973	5'flanking	A/G	NC	A	A	A	A	A	A	A	A	A	A	A	A	A	A	A	G	A
**rs240249171**	75709613	E030042O20Rik	4972	-4972	5'flanking	C/T	NC	C	C	C	C	C	C	C	C	C	C	C	C	C	C	C	T	C
**rs226991224**	75709598	E030042O20Rik	4957	-4957	5'flanking	A/G	NC	G	G	G	G	G	G	G	G	G	G	G	G	G	G	G	A	G
**rs33255540**	75709516	E030042O20Rik	4875	-4875	5'flanking	A/G	NC	G	A	A	G	A	A	G	G	A	A	G	A	G	A	G	G	G
**rs247536675**	75709480	E030042O20Rik	4839	-4839	5'flanking	C/T	NC	C	C	C	C	C	C	C	C	C	C	C	C	C	C	C	T	C
**rs221147315**	75709474	E030042O20Rik	4833	-4833	5'flanking	G/T	NC	G	G	G	G	G	G	G	G	G	G	G	G	G	G	T	G	G
**rs255873241**	75709455	E030042O20Rik	4814	-4814	5'flanking	A/G	NC	G	G	G	G	G	G	G	A	G	G	G	G	G	G	G	G	G
**rs247277782**	75709424	E030042O20Rik	4783	-4783	5'flanking	C/G	NC	G	G	G	G	G	G	G	C	G	G	G	G	G	G	G	G	G
**rs219830336**	75709421	E030042O20Rik	4780	-4780	5'flanking	A/G	NC	G	G	G	G	G	G	G	G	G	G	G	G	G	G	G	A	G
**rs257294435**	75709420	E030042O20Rik	4779	-4779	5'flanking	C/T	NC	T	T	T	T	T	T	T	C	T	T	T	T	C	T	C	C	C
**rs234599241**	75709176	E030042O20Rik	4535	-4535	5'flanking	A/G	I	G	G	G	G	G	G	G	G	G	G	G	G	G	G	G	A	G
**rs237178090**	75709152	E030042O20Rik	4511	-4511	5'flanking	C/T	I	C	C	C	C	C	C	C	C	C	C	C	C	T	C	C	C	T
**rs264066598**	75709124	E030042O20Rik	4483	-4483	5'flanking	C/T	I	T	T	T	T	T	T	T	T	T	T	T	T	T	T	T	C	T
**rs239177899**	75709120	E030042O20Rik	4479	-4479	5'flanking	A/G	I	A	A	A	A	A	A	A	A	A	A	A	A	A	A	A	G	A
**rs29868165**	75709012	E030042O20Rik	4371	-4371	5'flanking	A/G	I	A	G	G	A	G	G	A	G	G	G	A	G	G	G	G	G	G
**rs249142851**	75709011	E030042O20Rik	4370	-4370	5'flanking	C/T	I	C	C	C	C	C	C	C	C	C	C	C	C	C	C	T	C	C
**rs243227340**	75709003	E030042O20Rik	4362	-4362	5'flanking	A/G	I	G	G	G	G	G	G	G	G	G	G	G	G	A	G	G	A	A
**rs213568400**	75708971	E030042O20Rik	4330	-4330	5'flanking	C/T	I	T	T	T	T	T	T	T	T	T	T	T	T	T	T	T	C	T
**rs245869742**	75708881	E030042O20Rik	4240	-4240	5'flanking	C/T	I	T	T	T	T	T	T	T	T	T	T	T	T	T	T	C	T	T
**rs226690726**	75708854	E030042O20Rik	4213	-4213	5'flanking	C/T	I	T	T	T	T	T	T	T	T	T	T	T	T	T	T	T	C	T
**rs260067802**	75708817	E030042O20Rik	4176	-4176	5'flanking	C/T	I	C	C	C	C	C	C	C	C	C	C	C	C	C	C	C	T	C
**rs250341742**	75708706	E030042O20Rik	4065	-4065	5'flanking	A/T	I	A	A	A	A	A	A	A	A	A	A	A	A	A	A	A	T	A
**rs228899359**	75708700	E030042O20Rik	4059	-4059	5'flanking	A/G	I	A	A	A	A	A	A	A	A	A	A	A	A	A	A	G	A	A
**rs264666949**	75708655	E030042O20Rik	4014	-4014	5'flanking	A/G	I	G	G	G	G	G	G	G	G	G	G	G	G	G	G	G	A	G
**rs244676293**	75708654	E030042O20Rik	4013	-4013	5'flanking	C/T	I	T	T	T	T	T	T	T	T	T	T	T	T	T	T	T	C	T
**rs50763314**	75708597	E030042O20Rik	3956	-3956	5'flanking	C/G	I	C	C	C	C	C	C	C	C	C	C	C	C	C	C	C	G	C
**rs219885776**	75708592	E030042O20Rik	3951	-3951	5'flanking	C/G/T	I	T	T	T	T	T	T	T	C	T	T	T	T	T	T	T	T	T
**rs33051362**	75708450	E030042O20Rik	3809	-3809	5'flanking	G/T	I	T	G	G	T	G	G	T	G	G	G	T	G	G	G	G	G	G
**rs240796647**	75708422	E030042O20Rik	3781	-3781	5'flanking	C/T	I	C	C	C	C	C	C	C	T	C	C	C	C	C	C	C	C	C
**rs223681637**	75708244	E030042O20Rik	3603	-3603	5'flanking	C/T	I	T	T	T	T	T	T	T	T	T	T	T	T	T	T	T	C	T
**rs260734210**	75708243	E030042O20Rik	3602	-3602	5'flanking	G/T	I	T	T	T	T	T	T	T	T	T	T	T	T	T	T	G	T	T
**rs241799886**	75708217	E030042O20Rik	3576	-3576	5'flanking	C/T	I	C	C	C	C	C	C	C	C	C	C	C	C	C	C	C	T	C
**rs212540446**	75708209	E030042O20Rik	3568	-3568	5'flanking	C/T	I	T	T	T	T	T	T	T	T	T	T	T	T	T	T	T	C	T
**rs260621619**	75708149	E030042O20Rik	3508	-3508	5'flanking	A/T	I	A	A	A	A	A	A	A	A	A	A	A	A	A	A	A	T	A
**rs231801047**	75708145	E030042O20Rik	3504	-3504	5'flanking	C/T	I	C	C	C	C	C	C	C	C	C	C	C	C	C	C	C	T	C
**rs213630694**	75708138	E030042O20Rik	3497	-3497	5'flanking	A/G	I	G	G	G	G	G	G	G	G	G	G	G	G	G	G	G	A	G
**rs245929997**	75708137	E030042O20Rik	3496	-3496	5'flanking	A/G	I	G	G	G	G	G	G	G	G	G	G	G	G	G	G	G	A	G
**rs226749174**	75708115	E030042O20Rik	3474	-3474	5'flanking	G/T	I	T	T	T	T	T	T	T	T	T	T	T	T	T	T	T	G	T
**rs218909371**	75708045	E030042O20Rik	3404	-3404	5'flanking	C/T	I	T	T	T	T	T	T	T	T	T	T	T	T	T	T	T	C	T
**rs254123930**	75707907	E030042O20Rik	3266	-3266	5'flanking	C/T	I	C	C	C	C	C	C	C	C	C	C	C	C	C	C	C	T	C
**rs229438622**	75707710	E030042O20Rik	3069	-3069	5'flanking	C/T	I	C	C	C	C	C	C	C	C	C	C	C	C	C	C	T	C	C
**rs261595945**	75707695	E030042O20Rik	3054	-3054	5'flanking	A/G	I	G	G	G	G	G	G	G	G	G	G	G	G	G	G	G	A	G
**rs256370335**	75707685	E030042O20Rik	3044	-3044	5'flanking	A/T	I	T	T	T	T	T	T	T	T	T	T	T	T	T	T	T	A	T
**rs225800735**	75707627	E030042O20Rik	2986	-2986	5'flanking	C/T	I	T	T	T	T	T	T	T	T	T	T	T	T	T	T	T	C	T
**rs260304414**	75707610	E030042O20Rik	2969	-2969	5'flanking	C/G	I	G	G	G	G	G	G	G	G	G	G	G	G	G	G	G	C	G
**rs240859579**	75707591	E030042O20Rik	2950	-2950	5'flanking	A/G	I	G	G	G	G	G	G	G	G	G	G	G	G	G	G	G	A	G
**rs247435176**	75707572	E030042O20Rik	2931	-2931	5'flanking	C/T	I	T	T	T	T	T	T	T	C	T	T	T	T	C	T	C	C	C
**rs265995066**	75707537	E030042O20Rik	2896	-2896	5'flanking	C/T	I	T	T	T	T	T	T	T	T	T	T	T	T	T	T	T	C	T
**rs247215518**	75707514	E030042O20Rik	2873	-2873	5'flanking	G/T	I	G	G	G	G	G	G	G	G	G	G	G	G	G	G	G	T	G
**rs220803614**	75707513	E030042O20Rik	2872	-2872	5'flanking	C/T	I	T	T	T	T	T	T	T	T	T	T	T	T	T	T	T	C	T
**rs33595806**	75707504	E030042O20Rik	2863	-2863	5'flanking	C/T	I	C	T	T	C	T	T	C	T	T	T	C	T	T	T	T	T	T
**rs231859891**	75707491	E030042O20Rik	2850	-2850	5'flanking	A/C	I	A	A	A	A	A	A	A	A	A	A	A	A	A	A	C	A	A
**rs263410910**	75707478	E030042O20Rik	2837	-2837	5'flanking	A/C	I	A	A	A	A	A	A	A	C	A	A	A	A	C	A	C	C	C
**rs32828299**	75707404	E030042O20Rik	2763	-2763	5'flanking	A/G	I	A	G	G	A	G	G	A	G	G	G	A	G	G	G	G	A	G
**rs232815688**	75707385	E030042O20Rik	2744	-2744	5'flanking	G/T	I	T	T	T	T	T	T	T	T	T	T	T	T	T	T	T	G	T
**rs219058923**	75707337	E030042O20Rik	2696	-2696	5'flanking	A/T	I	T	T	T	T	T	T	T	T	T	T	T	T	T	T	T	A	T
**rs27978297**	75707281	E030042O20Rik	2640	-2640	5'flanking	C/T	I	T	T	T	T	T	T	T	T	T	T	T	T	T	T	C	T	T
**rs27978298**	75707263	E030042O20Rik	2622	-2622	5'flanking	C/T	I	C	C	C	C	C	C	C	T	C	C	C	C	C	C	C	C	C
**rs257363084**	75707231	E030042O20Rik	2590	-2590	5'flanking	A/T	I	A	A	A	A	A	A	A	T	A	A	A	A	T	A	A	A	T
**rs245853837**	75707183	E030042O20Rik	2542	-2542	5'flanking	G/T	I	T	T	T	T	T	T	T	T	T	T	T	T	T	T	T	G	T
**rs225868145**	75707172	E030042O20Rik	2531	-2531	5'flanking	A/T	I	A	A	A	A	A	A	A	A	A	A	A	A	A	A	A	T	A
**rs259982121**	75707150	E030042O20Rik	2509	-2509	5'flanking	C/G	I	G	G	G	G	G	G	G	G	G	G	G	G	G	G	G	C	G
**rs246896451**	75707139	E030042O20Rik	2498	-2498	5'flanking	A/G	I	G	G	G	G	G	G	G	G	G	G	G	G	G	G	A	G	G
**rs235728646**	75707132	E030042O20Rik	2491	-2491	5'flanking	C/T	I	C	C	C	C	C	C	C	C	C	C	C	C	C	C	T	C	C
**rs264187040**	75706924	E030042O20Rik	2283	-2283	5'flanking	A/G	I	A	A	A	A	A	A	A	A	A	A	A	A	A	A	A	A	G
**rs27978299**	75706892	E030042O20Rik	2251	-2251	5'flanking	G/T	I	G	G	G	G	G	G	G	T	G	G	G	G	G	G	G	G	G
**rs27978300**	75706861	E030042O20Rik	2220	-2220	5'flanking	A/G	I	G	G	G	G	G	G	G	G	G	G	G	G	G	G	A	G	G

ID	Ch2 bp Location	Gene	Distance (gene)	Distance (mRNA)	Region	Alleles (genome)	Consequence	C57BL/6J	129S1/SvImJ	A/J	AKR/J	BALB/cJ	C3H/HeJ	C57BL/6NJ	CAST/EiJ	CBA/J	DBA/2J	FVB/NJ	LP/J	NOD/ShiLtJ	NZO/HILtJ	PWK/PhJ	SPRET/EiJ	WSB/EiJ
**rs27978301**	75706760	E030042O20Rik	2119	-2119	5'flanking	A/T	I	T	T	T	T	T	T	T	T	T	T	T	T	A	T	T	T	A
**rs238139731**	75706739	E030042O20Rik	2098	-2098	5'flanking	A/G	I	G	G	G	G	G	G	G	A	G	G	G	G	G	G	G	G	A
**rs219122423**	75706732	E030042O20Rik	2091	-2091	5'flanking	C/G	I	G	G	G	G	G	G	G	G	G	G	G	G	G	G	G	C	G
**rs27978302**	75706721	E030042O20Rik	2080	-2080	5'flanking	A/G	I	A	A	A	A	A	A	A	G	A	A	A	A	G	A	A	G	G
**rs27978303**	75706591	E030042O20Rik	1950	-1950	5'flanking	C/T	I	C	C	C	C	C	C	C	T	C	C	C	C	C	C	C	C	C
**rs227131200**	75706486	E030042O20Rik	1845	-1845	5'flanking	A/T	I	T	T	T	T	T	T	T	T	T	T	T	T	T	T	T	A	T
**rs33316784**	75706455	E030042O20Rik	1814	-1814	5'flanking	A/T	I	T	A	A	T	A	A	T	A	A	A	T	A	A	A	A	A	A
**rs27978304**	75706387	E030042O20Rik	1746	-1746	5'flanking	C/T	I	C	C	C	C	C	C	C	T	C	C	C	C	T	C	T	C	T
**rs27978305**	75706326	E030042O20Rik	1685	-1685	5'flanking	C/G	I	G																
**rs266000646**	75706320 - 75706321	E030042O20Rik	1680-1679	-1680:-1679	5'flanking	AAAC add	I	AA																
**rs218612804**	75706311	E030042O20Rik	1670	-1670	5'flanking	T del	I	T																
**rs236201595**	75706286-75706296	E030042O20Rik	1655-1645	-1655:-1645	5'flanking	CAAACAGAATG del	I	CAAACAG AATG																
**rs211905706**	75706239	E030042O20Rik	1598	-1598	5'flanking	G/T	I	T	T	T	T	T	T	T	T	T	T	T	T	T	T	T	G	T
**rs245907677**	75706152	E030042O20Rik	1511	-1511	5'flanking	C/T	I	C	C	C	C	C	C	C	C	C	C	C	C	C	C	C	T	C
**rs231965425**	75706141	E030042O20Rik	1500	-1500	5'flanking	A/T	I	A	A	A	A	A	A	A	A	A	A	A	A	A	A	A	T	A
**rs216029209**	75706088	E030042O20Rik	1447	-1447	5'flanking	A/G	I	A	A	A	A	A	A	A	A	A	A	A	A	A	A	A	G	A
**rs246569186**	75706056	E030042O20Rik	1415	-1415	5'flanking	A/C	I	A	A	A	A	A	A	A	A	A	A	A	A	A	A	A	C	A
**rs225603345**	75706018	E030042O20Rik	1377	-1377	5'flanking	G/T	I	G																
**rs219655931**	75705967-75705973	E030042O20Rik	1332-1326	-1332:-1326	5'flanking	TTTTTTT del	I	TTTTTTT																
**rd233902846**	75705966	E030042O20Rik	1325	-1325	5'flanking	A/T	I	A																
**rs214254775**	75705965-75708966	E030042O20Rik	1324	-1324	5'flanking	TTTTTTTTTTTTTTTTT add	I	AA																
**rs235569319**	75705858	E030042O20Rik	1217	-1217	5'flanking	A del	I	A																
**rs250669934**	75705818-75705819	E030042O20Rik	1177	-1177	5'flanking	A add	I	GA																
**rs215402334**	75705761-75705766	E030042O20Rik	1120	-1120	5'flanking	GTGGTA del	I	GTGGTA																
**rs218589635**	75705741	E030042O20Rik	1100	-1100	5'flanking	A/T	I	T	T	T	T	T	T	T	T	T	T	T	T	T	T	T	A	T
**rs250243737**	75705738	E030042O20Rik	1097	-1097	5'flanking	A/G	I	A	A	A	A	A	A	A	A	A	A	A	A	A	A	A	G	A
**rs228660677**	75705668	E030042O20Rik	1027	-1027	5'flanking	A/G	I	G	G	G	G	G	G	G	G	G	G	G	G	G	G	G	A	G
**rs256608517**	75705565	E030042O20Rik	924	-924	5'flanking	A/G	I	G	G	G	G	G	G	G	G	G	G	G	G	G	G	A	A	G
**rs47274959**	75705562	E030042O20Rik	921	-921	5'flanking	A/T	I	A	T	T	A	T	T	A	A	T	T	A	T	A	T	T	T	A
**rs216940398**	75705554-75705555	E030042O20Rik	913	-913	5'flanking	T add	I	CG																
**rs239114134**	75705540	E030042O20Rik	899	-899	5'flanking	C/T	I	C	T	T	C	T	T	C	C	T	T	C	T	C	T	C	T	C
**rs46461765**	75705528	E030042O20Rik	887	-887	5'flanking	G/T	I	T	G	G	T	G	G	T	T	G	G	T	G	T	G	G	G	T
**rs51449853**	75705525	E030042O20Rik	884	-884	5'flanking	A/C	I	C	A	A	C	A	A	C	C	A	A	C	A	C	A	C	C	C
**rs249093111**	75705512	E030042O20Rik	871	-871	5'flanking	C/T	I	C	T	T	C	T	T	C	C	T	T	C	T	C	T	T	T	C
**rs263745200**	75705510	E030042O20Rik	869	-869	5'flanking	C/G	I	G	G	G	G	G	G	G	G	G	G	G	G	G	G	C	G	G
**rs45651867**	75705498	E030042O20Rik	857	-857	5'flanking	G/T	I	T	G	G	T	G	G	T	T	G	G	T	G	T	G	G	G	T
**rs219997531**	75705495	E030042O20Rik	854	-854	5'flanking	A/G	SSD	G	G	G	G	G	G	G	G	G	G	G	G	G	G	G	A	G
**rs251918379**	75705451	E030042O20Rik	810	-810	5'flanking	A/T	NC	T	T	T	T	T	T	T	T	T	T	T	T	T	T	T	A	T
**rs27978306**	75705449	E030042O20Rik	808	-808	5'flanking	A/T	NC	T	A	A	T	A	A	T	T	A	A	T	A	T	A	T	T	T
**rs216087412**	75705429	E030042O20Rik	788	-788	5'flanking	A/C	NC	A	A	A	A	A	A	A	A	A	A	A	A	A	A	C	A	A
**rs261229914**	75705423-75705424	E030042O20Rik	783	-783	5'flanking	AG del	NC	AG																
**rs27978307**	75705400	E030042O20Rik	759	-759	5'flanking	C/T	NC	C	C	C	C	C	C	C	C	C	C	C	C	C	C	T	C	C
**rs243167395**	75705359	E030042O20Rik	718	-718	5'flanking	A/G	NC	G	G	G	G	G	G	G	G	G	G	G	G	G	G	G	A	G
**rs27978308**	75705307	E030042O20Rik	666	-666	5'flanking	A/G	NC	G	G	G	G	G	G	G	G	G	G	G	G	G	G	A	G	G
**rs254744098**	75705294	E030042O20Rik	653	-653	5'flanking	A/T	NC	A	A	A	A	A	A	A	A	A	A	A	A	A	A	A	T	A
**rs228133419**	75705179	E030042O20Rik	538	-538	5'flanking	G/T	NC	G	G	G	G	G	G	G	G	G	G	G	G	G	G	G	T	G
**rs214719520**	75705111	E030042O20Rik	470	-470	5'flanking	A/G	NC	G	G	G	G	G	G	G	G	G	G	G	G	G	G	G	A	G
**rs244087730**	75705101	E030042O20Rik	460	-460	5'flanking	A/G	NC	G	G	G	G	G	G	G	G	G	G	G	G	G	G	G	A	G
**rs227781699**	75705081	E030042O20Rik	440	-440	5'flanking	C/G	NC	C	C	C	C	C	C	C	C	C	C	C	C	C	C	G	C	C
**rs257870353**	75705067	E030042O20Rik	426	-426	5'flanking	C/T	NC	C	C	C	C	C	C	C	C	C	C	C	C	C	C	T	C	C
**rs244877440**	75705022	E030042O20Rik	381	-381	5'flanking	A/G	NC	A	A	A	A	A	A	A	A	A	A	A	A	A	A	A	G	A
**rs234628138**	75704973	E030042O20Rik	332	-332	5'flanking	A/C	NC	A	A	A	A	A	A	A	A	A	A	A	A	A	A	A	C	A
**rs247275247**	75704961	E030042O20Rik	320	-320	5'flanking	A/G	NC	G	G	G	G	G	G	G	G	G	G	G	G	A	G	G	G	G
**rs247519047**	75704922	E030042O20Rik	280	-281	5'flanking	G del	NC	G																
**rs27978309 (rs51915758)**	75704887	E030042O20Rik	246	-246	5'flanking	A/G	NC	G	A	A	G	A	A	G	G	A	A	G	A	G	A	C	C	G
**rs218139102**	75704879	E030042O20Rik	238	-238	5'flanking	A/C	NC	C	C	C	C	C	C	C	C	C	C	C	C	C	C	A	C	C
**rs251990355**	75704870	E030042O20Rik	229	-229	5'flanking	C/T	NC	T	T	T	T	T	T	T	T	T	T	T	T	T	T	T	C	T
**rs238746955**	75704794	E030042O20Rik	153	-153	5'flanking	A/G	NC	A	A	A	A	A	A	A	A	A	A	A	A	A	A	G	A	A
**rs221664405**	75704786	E030042O20Rik	145	-145	5'flanking	A/G	NC	A	A	A	A	A	A	A	A	A	A	A	A	A	A	A	G	A

ID	Ch2 bp Location	Gene	Distance (gene)	Distance (mRNA)	Region	Alleles (genome)	Consequence	C57BL/6J	129S1/SvImJ	A/J	AKR/J	BALB/cJ	C3H/HeJ	C57BL/6NJ	CAST/EiJ	CBA/J	DBA/2J	FVB/NJ	LP/J	NOD/ShiLtJ	NZO/HlLtJ	PWK/PhJ	SPRET/EiJ	WSB/EiJ
**rs217054035**	75704766-75704767	E030042O20Rik	125	-125	5'flanking	GAA add	NC	GA																
**rs248182931**	75704759-75704760	E030042O20Rik	118	-118	5'flanking	G add	NC	AG																
**rs217197904**	75704744	E030042O20Rik	103	-103	5'flanking	A/G	NC	G	A	A	G	A	A	G	G	A	A	G	A	A	A	G	G	A
**rs264493649**	75704704	Nfe2l2	63	-63	5'flanking	G del	NC	G																
**rs240481112**	75704682	Nfe2l2	41	-41	5'flanking	C/T	NC	C	C	C	C	C	C	C	C	C	C	C	C	C	C	C	T	C
**rs27978310**	75704617	Nfe2l2	25	25	UTR-5	A/G		A	A	A	A	A	A	A	A	A	A	A	A	A	A	G	G	A
**rs27978311**	75704610	Nfe2l2	32	32	UTR-5	C/G		G	G	G	G	G	G	G	G	G	G	G	G	G	G	G	G	C
**rs27978312**	75704605	Nfe2l2	37	37	UTR-5	A/G		G	A	A	G	A	A	G	G	A	A	G	A	G	A	G	G	G
**rs214784220**	75704499	Nfe2l2	143	143	UTR-5	A/G		A	A	A	A	A	A	A	A	A	A	A	A	A	A	G	G	A
**rs244146318**	75704498	Nfe2l2	144	144	UTR-5	G/T		G	G	G	G	G	G	G	G	G	G	G	G	G	G	G	T	G
**rs27978313**	75704497	Nfe2l2	145	145	UTR-5	C/G		G	C	C	G	C	C	G	C	C	C	G	C	C	C	C	C	C
**rs215431944**	75704493	Nfe2l2	149	149	UTR-5	C/T		C	C	C	C	C	C	C	C	C	C	C	C	C	C	T	C	C
**rs257015697**	75704449	Nfe2l2	193	193	UTR-5	A/G		G	G	G	G	G	G	G	G	G	G	G	G	G	G	G	A	G
**rs252234782**	75704419	Nfe2l2	223	223	UTR-5	G/T		G																
**rs27978314**	75704319	Nfe2l2	322		intron1	A/C	I	C	C	C	C	C	C	C	C	C	C	C	C	A	C	C	C	C
**rs265852411**	75704201	Nfe2l2	440		intron1	C/T	I	C	C	C	C	C	C	C	C	C	C	C	C	C	C	C	T	C
**rs249576378**	75704200	Nfe2l2	441		intron1	A/G	I	G	G	G	G	G	G	G	G	G	G	G	G	G	G	G	A	G
**rs223884135**	75704162	Nfe2l2	479		intron1	C/T	I	C	C	C	C	C	C	C	C	C	C	C	C	C	C	T	C	C
**rs225675309**	75704123-75704123	Nfe2l2	518-519		intron1	GC del	I	GC																
**rs27978315**	75704018	Nfe2l2	623		intron1	C/T	I	C	C	C	C	C	C	C	T	C	C	C	C	C	C	C	C	C
**rs27978316**	75704005	Nfe2l2	636		intron1	A/G	I	A	A	A	A	A	A	A	A	A	A	A	A	A	A	G	A	A
**rs221730892**	75703948	Nfe2l2	693		intron1	A/T	I	A	A	A	A	A	A	A	T	A	A	A	A	A	A	A	A	A
**rs230812739**	75703907	Nfe2l2	734		intron1	A/G	I	A	A	A	A	A	A	A	A	A	A	A	A	A	A	G	A	A
**rs251675588**	75703902	Nfe2l2	739		intron1	A/G	I	G	G	G	G	G	G	G	G	G	G	G	G	G	G	G	A	G
**rs257204870**	75703882	Nfe2l2	759		intron1	C/T	I	T	C	C	T	C	C	T	C	C	C	T	C	C	C	C	C	C
**rs29516127**	75703881	Nfe2l2	760		intron1	A/G	I	G	A	A	G	A	A	G	A	A	A	G	A	A	A	A	A	A
**rs230074987**	75703862	Nfe2l2	779		intron1	C/T	I	C	C	C	C	C	C	C	T	C	C	C	C	C	C	C	C	C
**rs220065806**	75703840	Nfe2l2	801		intron1	C/G	I	G	G	G	G	G	G	G	C	G	G	G	G	G	G	G	G	G
**rs250109356**	75703741	Nfe2l2	900		intron1	G/T	I	G	G	G	G	G	G	G	G	G	G	G	G	G	G	G	T	G
**rs233891721**	75703735	Nfe2l2	906		intron1	C/T	I	T	T	T	T	T	T	T	T	T	T	T	T	T	T	C	C	T
**rs215493467**	75703725	Nfe2l2	916		intron1	C/T	I	T	T	T	T	T	T	T	T	T	T	T	T	T	T	T	C	T
**rs27978317**	75703616	Nfe2l2	1025		intron1	C/G	I	C	C	C	C	C	C	C	C	C	C	C	C	C	C	G	G	C
**rs242363061**	75703586	Nfe2l2	1055		intron1	A/T	I	T	T	T	T	T	T	T	T	T	T	T	T	T	T	T	A	T
**rs217708190**	75703584	Nfe2l2	1057		intron1	C/T	I	C	C	C	C	C	C	C	C	C	C	C	C	C	C	C	T	C
**rs27978318**	75703390	Nfe2l2	1251		intron1	C/T	I	T	T	T	T	T	T	T	T	T	T	T	T	T	T	C	T	T
**rs32866119**	75703337	Nfe2l2	1304		intron1	C/T	I	T	C	C	T	C	C	T	T	C	C	T	C	C	C	T	T	C
**rs258012361**	75703270	Nfe2l2	1371		intron1	A/G	I	G	G	G	G	G	G	G	G	G	G	G	G	G	G	A	G	G
**rs245014956**	75703207	Nfe2l2	1434		intron1	C/T	I	T	T	T	T	T	T	T	T	T	T	T	T	T	T	T	C	T
**rs214557169**	75703194-75703193	Nfe2l2	1447-1448		intron1	ATA add	I	CA																
**rs256917024**	75703191-75703189	Nfe2l2	1450-1452		intron1	TCT del	I	TCT																
**rs228081029**	75703098	Nfe2l2	1543		intron1	C/T	I	T	T	T	T	T	T	T	T	T	T	T	T	T	T	C	T	T
**rs265483792**	75703093	Nfe2l2	1548		intron1	C/G	I	C	C	C	C	C	C	C	C	C	C	C	C	C	C	G	G	C
**rs248870906**	75703058-75703053	Nfe2l2	1583-1588		intron1	GCGCGC del	I	GCGCGC																
**rs222730436**	75703049-75703054	Nfe2l2	1587-1592		intron1	GTGTGC del	I	GTGTGC																
**rs248213530**	75703015	Nfe2l2	1626		intron1	C/T	I	C	C	C	C	C	C	C	C	C	C	C	C	C	C	T	C	C
**rs261488118**	75702906	Nfe2l2	1735		intron1	C/T		T																
**rs27978319**	75702905	Nfe2l2	1736		intron1	A/G	I	G	A	A	G	A	A	G	G	A	A	G	A	A	A	G	G	A
**rs262043662**	75702870	Nfe2l2	1771		intron1	A/T	I	A	A	A	A	A	A	A	A	A	A	A	A	A	A	T	A	A
**rs236022496**	75702862	Nfe2l2	1779		intron1	C/T	I	T	T	T	T	T	T	T	T	T	T	T	T	T	T	C	T	T
**rs220126184**	75702858	Nfe2l2	1783		intron1	C/T	I	C	C	C	C	C	C	C	C	C	C	C	C	C	C	C	T	C
**rs27978320**	75702780	Nfe2l2	1861		intron1	C/T	I	T	T	T	T	T	T	T	C	T	T	T	T	T	T	T	T	T
**rs240869005**	75702714-75702713		1927-1928		intron1	AA del	I	AA																
**rs233599535**	75702679	Nfe2l2	1962		intron1	C/T	I	T	T	T	T	T	T	T	T	T	T	T	T	T	T	C	C	T
**rs224574410**	75702653	Nfe2l2	1988		intron1	G/T	I	G	G	G	G	G	G	G	G	G	G	G	G	G	G	G	T	G
**rs27978321**	75702589	Nfe2l2	2052		intron1	A/G	I	A	A	A	A	A	A	A	A	A	A	A	A	A	A	G	A	A
**rs239796144**	75702550	Nfe2l2	2091		intron1	C/G	I	C	C	C	C	C	C	C	C	C	C	C	C	C	C	C	G	C
**rs213569731**	75702507	Nfe2l2	2134		intron1	A/G	I	A	A	A	A	A	A	A	A	A	A	A	A	A	A	A	G	A
**rs27978322**	75702503	Nfe2l2	2138		intron1	C/T	I	T	T	T	T	T	T	T	C	T	T	T	T	T	T	T	C	T
**rs229742175**	75702472	Nfe2l2	2169		intron1	A/G	I	A	A	A	A	A	A	A	A	A	A	A	A	A	A	G	A	A
**rs214136071**	75702467	Nfe2l2	2174		intron1	A/G	I	G	G	G	G	G	G	G	G	G	G	G	G	G	G	G	A	G
**rs244703714**	75702466	Nfe2l2	2175		intron1	C/T	I	T	T	T	T	T	T	T	T	T	T	T	T	T	T	C	C	T

ID	Ch2 bp Location	Gene	Distance (gene)	Distance (mRNA)	Region	Alleles (genome)	Consequence	C57BL/6J	129S1/SvImJ	A/J	AKR/J	BALB/cJ	C3H/HeJ	C57BL/6NJ	CAST/EiJ	CBA/J	DBA/2J	FVB/NJ	LP/J	NOD/ShiLtJ	NZO/HlLtJ	PWK/PhJ	SPRET/EiJ	WSB/EiJ
**rs260962476**	75702453-75702449	Nfe2l2	2188-2192		intron1	AAAAC del	I	AAAAC																
**rs227780357**	75702429	Nfe2l2	2236		intron1	G/T	I	T	T	T	T	T	T	T	T	T	T	T	T	T	T	T	G	T
**rs215718442**	75702418	Nfe2l2	2223		intron1	A/G	I	A	A	A	A	A	A	A	A	A	A	A	A	A	A	A	G	A
**rs27978323**	75702365	Nfe2l2	2276		intron1	A/G	I	A	A	A	A	A	A	A	A	A	A	A	A	A	A	G	G	A
**rs233027754**	75702347	Nfe2l2	2294		intron1	A/G	I	A	A	A	A	A	A	A	A	A	A	A	A	A	A	A	G	A
**rs264824686**	75702311	Nfe2l2	2330		intron1	C/T	I	C	C	C	C	C	C	C	C	C	C	C	C	C	C	T	C	C
**rs236087283**	75702243	Nfe2l2	2398		intron1	G/T	I	G	G	G	G	G	G	G	G	G	G	G	G	G	G	G	T	G
**rs226255008**	75702206	Nfe2l2	2435		intron1	A/G	I	G	G	G	G	G	G	G	G	G	G	G	G	G	G	A	G	G
**rs255552658**	75702188	Nfe2l2	2453		intron1	A/G	I	G	G	G	G	G	G	G	G	G	G	G	G	G	G	A	A	G
**rs240122691**	75702088	Nfe2l2	2553		intron1	C/T	I	C	C	C	C	C	C	C	C	C	C	C	C	C	C	C	T	C
**rs224257344**	75702048	Nfe2l2	2593		intron1	A/G	I	A	A	A	A	A	A	A	A	A	A	A	A	A	A	A	G	A
**rs261199728**	75702037	Nfe2l2	2604		intron1	C/T	I	C	C	C	C	C	C	C	C	C	C	C	C	C	C	T	C	C
**rs250628545**	75702004	Nfe2l2	2637		intron1	C/T	I	C	C	C	C	C	C	C	T	C	C	C	C	C	C	C	C	C
**rs222043619**	75701973	Nfe2l2	2668		intron1	C/T	I	T	T	T	T	T	T	T	T	T	T	T	T	T	T	T	C	T
**rs27978324**	75701971	Nfe2l2	2670		intron1	A/C	I	C	C	C	C	C	C	C	C	C	C	C	C	C	C	A	C	C
**rs233141858**	75701918	Nfe2l2	2723		intron1	G/T	I	T	T	T	T	T	T	T	T	T	T	T	T	T	T	T	G	T
**rs214186430**	75701868	Nfe2l2	2773		intron1	C/T	I	T	T	T	T	T	T	T	C	T	T	T	T	T	T	T	C	T
**rs250976411**	75701863	Nfe2l2	2778		intron1	C/T	I	C	C	C	C	C	C	C	C	C	C	C	C	C	C	C	T	C
**rs27978325**	75701848	Nfe2l2	2793		intron1	C/T	I	C	C	C	C	C	C	C	C	C	C	C	C	C	C	T	C	C
**rs27978326**	75701762	Nfe2l2	2879		intron1	A/G	I	G	G	G	G	G	G	G	A	G	G	G	G	G	G	G	G	G
**rs254720551**	75701732	Nfe2l2	2909		intron1	C/G	I	G	G	G	G	G	G	G	G	G	G	G	G	G	G	G	C	G
**rs234035577**	75701686	Nfe2l2	2955		intron1	C/T	I	T	T	T	T	T	T	T	T	T	T	T	T	T	T	T	C	T
**rs212749974**	75701672	Nfe2l2	2969		intron1	C/T	I	C	C	C	C	C	C	C	T	C	C	C	C	C	C	T	C	C
**rs242203906**	75701670	Nfe2l2	2971		intron1	C/T	I	T	T	T	T	T	T	T	T	T	T	T	T	T	T	T	C	T
**rs225922718**	75701662	Nfe2l2	2979		intron1	C/T	I	T	T	T	T	T	T	T	T	T	T	T	T	T	T	T	C	T
**rs255621662**	75701649	Nfe2l2	2992		intron1	C/T	I	C	C	C	C	C	C	C	C	C	C	C	C	C	C	C	T	C
**rs240187610**	75701642	Nfe2l2	2999		intron1	C/T	I	T	T	T	T	T	T	T	T	T	T	T	T	T	T	T	C	T
**rs231756366**	75701617	Nfe2l2	3024		intron1	A/G	I	G	G	G	G	G	G	G	G	G	G	G	G	G	G	A	G	G
**rs266157706**	75701615	Nfe2l2	3026		intron1	G/T	I	T	T	T	T	T	T	T	T	T	T	T	T	T	T	T	G	T
**rs248000702**	75701597	Nfe2l2	3044		intron1	A/C	I	A	A	A	A	A	A	A	A	A	A	A	A	A	A	A	C	A
**rs221566222**	75701586	Nfe2l2	3055		intron1	C/T	I	T	T	T	T	T	T	T	T	T	T	T	T	T	T	C	T	T
**rs256344369**	75701569	Nfe2l2	3072		intron1	A/T	I	T	T	T	T	T	T	T	T	T	T	T	T	T	T	T	A	T
**rs240013834**	75701489	Nfe2l2	3152		intron1	G/T	I	G	G	G	G	G	G	G	G	G	G	G	G	G	G	G	T	G
**rs219777858**	75701390	Nfe2l2	3251		intron1	C/T	I	C	C	C	C	C	C	C	C	C	C	C	C	C	C	C	T	C
**rs251041126**	75701351	Nfe2l2	3290		intron1	A/C	I	A	A	A	A	A	A	A	A	A	A	A	A	A	A	A	C	A
**rs240689536**	75701307	Nfe2l2	3334		intron1	C/T	I	T	T	T	T	T	T	T	T	T	T	T	T	T	T	T	C	T
**rs223937661**	75701219	Nfe2l2	3422		intron1	A/C	I	C	C	C	C	C	C	C	C	C	C	C	C	C	C	C	C	A
**rs264032099**	75701139	Nfe2l2	3502		intron1	C/T	I	C	C	C	C	C	C	C	C	C	C	C	C	C	C	C	T	C
**rs239115477**	75701066	Nfe2l2	3575		intron1	A/G	I	G	G	G	G	G	G	G	G	G	G	G	G	G	G	G	A	G
**rs27978327**	75701047	Nfe2l2	3594		intron1	A/C	I	A																
**rs212880751**	75701044	Nfe2l2	3597		intron1	C/T	I	T	T	T	T	T	T	T	C	T	T	T	T	T	T	C	C	T
**rs242263241**	75701043	Nfe2l2	3598		intron1	A/T	I	A	A	A	A	A	A	A	A	A	A	A	A	A	A	T	A	A
**rs232043389**	75701040	Nfe2l2	3601		intron1	G/T	I	T	T	T	T	T	T	T	G	T	T	T	T	T	T	T	T	T
**rs213520543**	75701028	Nfe2l2	3613		intron1	G/T	I	T	T	T	T	T	T	T	T	T	T	T	T	T	T	G	T	T
**rs253369439**	75701024	Nfe2l2	3617		intron1	A/G	I	G	G	G	G	G	G	G	G	G	G	G	G	G	G	A	G	G
**rs232343345**	75700958	Nfe2l2	3683		intron1	A/G	I	G	G	G	G	G	G	G	A	G	G	G	G	G	G	G	G	G
**rs264485839**	75700870	Nfe2l2	3771		intron1	A/T	I	T	T	T	T	T	T	T	A	T	T	T	T	T	T	A	T	T
**rs259262655**	75700850	Nfe2l2	3791		intron1	C/T	I	T	T	T	T	T	T	T	T	T	T	T	T	T	T	T	C	T
**rs228811958**	75700833	Nfe2l2	3808		intron1	A/T	I	T	T	T	T	T	T	T	T	T	T	T	T	T	T	A	T	T
**rs27978328**	75700826	Nfe2l2	3815		intron1	A/G	I	A	A	A	A	A	A	A	G	A	A	A	A	A	A	A	A	A
**rs240076902**	75700803	Nfe2l2	3838		intron1	A/G	I	G	G	G	G	G	G	G	G	G	G	G	G	G	G	G	A	G
**rs238377034**	75700784	Nfe2l2	3857		intron1	T del	I	T																
**rs219833542**	75700782	Nfe2l2	3859		intron1	C/T	I	T	T	T	T	T	T	T	C	T	T	T	T	T	T	T	C	T
**rs230390114**	75700752-75700751	Nfe2l2	3889-3890		intron1	AC del	I	AC																
**rs260185989**	75700733	Nfe2l2	3908		intron1	C/T	I	T	T	T	T	T	T	T	T	T	T	T	T	T	T	T	C	T
**rs225226082**	75700715	Nfe2l2	3926		intron1	C/T	I	T	T	T	T	T	T	T	C	T	T	T	T	T	T	T	T	T
**rs27978329**	75700710	Nfe2l2	3931		intron1	C/T	I	T	C	C	T	C	C	T	C	C	C	T	C	C	C	C	T	C
**rs260685884**	75700687	Nfe2l2	3954		intron1	C/T	I	T	T	T	T	T	T	T	C	T	T	T	T	T	T	T	T	T
**rs241722789**	75700666	Nfe2l2	3975		intron1	A/G	I	A	A	A	A	A	A	A	A	A	A	A	A	A	A	A	G	A
**rs27978330**	75700665	Nfe2l2	3976		intron1	A/G	I	G	G	G	G	G	G	G	A	G	G	G	G	G	G	G	A	G
**rs265063374**	75700628-75700627	Nfe2l2	4013-4014		intron1	C add	I	GC																
**rs27978331**	75700602	Nfe2l2	4039		intron1	C/T	I	T	C	C	T	C	C	T	C	C	C	T	C	C	C	C	C	C
**rs232099121**	75700593	Nfe2l2	4048		intron1	A/G	I	G	G	G	G	G	G	G	G	G	G	G	G	G	G	A	G	G
**rs266085131**	75700568	Nfe2l2	4073		intron1	T del		T																
**rs213566127**	75700504	Nfe2l2	4137		intron1	C/T	I	C	C	C	C	C	C	C	C	C	C	C	C	C	C	C	T	C
**rs258092388**	75700503	Nfe2l2	4138		intron1	C/T	I	T	T	T	T	T	T	T	C	T	T	T	T	T	T	C	T	T

ID	Ch2 bp Location	Gene	Distance (gene)	Distance (mRNA)	Region	Alleles (genome)	Consequence	C57BL/6J	129S1/SvImJ	A/J	AKR/J	BALB/cJ	C3H/HeJ	C57BL/6NJ	CAST/EiJ	CBA/J	DBA/2J	FVB/NJ	LP/J	NOD/ShiLtJ	NZO/HlLtJ	PWK/PhJ	SPRET/EiJ	WSB/EiJ
**rs250248795**	75700490-75700489	Nfe2l2	4151-4152		intron1	TT del	I	TT																
**rs250289826**	75700489-75700488	Nfe2l2	4152-4153		intron1	TTTTT add	I	GT																
**rs237709104**	75700488	Nfe2l2	4153		intron1	G/T	I	G	G	G	G	G	G	G	G	G	G	G	G	G	G	G	T	G
**rs27978332**	75700418	Nfe2l2	4223		intron1	A/G	I	A	A	A	A	A	A	A	G	A	A	A	A	A	A	G	G	G
**rs235731320**	75700378-75700377	Nfe2l2	4264-4265		intron1	AA del	I	AA																
**rs253630496**	75700377-75700376	Nfe2l2	4265-4267		intron1	AAAAAAAA add	I	TA																
**rs243192392**	75700376-75700375	Nfe2l2	4267-4268		intron1	A add	I	TT																
**rs220104858**	75700374	Nfe2l2	4267		intron1	T del	I	T																
**rs254061717**	75700325	Nfe2l2	4316		intron1	A/G	I	G	G	G	G	G	G	G	A	G	G	G	G	G	G	A	A	A
**rs247244706**	75700323-75700322	Nfe2l2	4319-4320		intron1	A add	I	GA																
**rs225075019**	75700299	Nfe2l2	4342		intron1	C/T	I	C	C	C	C	C	C	C	C	C	C	C	C	C	C	C	C	T
**rs216137472**	75700258-75700257	Nfe2l2	4383-4384		intron1	G add		TG																
**rs256087954**	75700240	Nfe2l2	4401		intron1	C/T	I	C	C	C	C	C	C	C	C	C	C	C	C	C	C	T	C	C
**rs246109890**	75700169	Nfe2l2	4472		intron1	A/G	I	G	G	G	G	G	G	G	A	G	G	G	G	G	G	G	G	G
**rs226147099**	75700149	Nfe2l2	4492		intron1	C/T	I	C	C	C	C	C	C	C	C	C	C	C	C	C	C	T	C	C
**rs260243630**	75700135	Nfe2l2	4506		intron1	A/C/G	I	A	A	A	A	A	A	A	C	A	A	A	A	A	A	G	A	A
**rs225266461**	75700089-75700088	Nfe2l2	4552-4553		intron1	CT del		CT																
**rs3655647**	75700064	Nfe2l2	4577		intron1	C/T	I	T	C	C	T	C	C	T	C	C	C	T	C	C	C	C	C	C
**rs234105226**	75700058	Nfe2l2	4583		intron1	A/C	I	C	C	C	C	C	C	C	C	C	C	C	C	C	C	C	A	C
**rs266085234**	75700048	Nfe2l2	4593		intron1	C/T	I	C	C	C	C	C	C	C	C	C	C	C	C	C	C	C	T	C
**rs3655622**	75700046	Nfe2l2	4595		intron1	C/T	I	C	T	T	C	T	T	C	C	T	T	C	T	T	T	C	C	C
**rs218415197**	75700039	Nfe2l2	4602		intron1	A/G	I	G	G	G	G	G	G	G	G	G	G	G	G	G	G	G	G	A
**rs247871958**	75700035	Nfe2l2	4606		intron1	A/G	I	G	G	G	G	G	G	G	G	G	G	G	G	G	G	G	A	A
**rs238010604**	75699964	Nfe2l2	4677		intron1	A/C	I	C	C	C	C	C	C	C	A	C	C	C	C	C	C	C	A	A
**rs219358518**	75699919	Nfe2l2	4722		intron1	A/C	I	A	A	A	A	A	A	A	A	A	A	A	A	A	A	A	C	A
**rs230156875**	75699896	Nfe2l2	4745		intron1	A/C	I	C	C	C	C	C	C	C	C	C	C	C	C	A	C	C	C	C
**rs239846346**	75699872	Nfe2l2	4769		intron1	A/G	I	A	A	A	A	A	A	A	G	A	A	A	A	A	A	A	G	A
**rs217927809**	75699779-75699776	Nfe2l2	4862-4865		intron1	TAAT del		TAAT																
**rs218977770**	75699770	Nfe2l2	4871		intron1	A/G	I	G	G	G	G	G	G	G	G	G	G	G	G	G	G	A	G	G
**rs263875019**	75699764	Nfe2l2	4877		intron1	C/T	I	T	T	T	T	T	T	T	T	T	T	T	T	T	T	C	T	T
**rs253399153**	75699737		4904		intron1	G del		G																
**rs230848523**	75699736	Nfe2l2	4905		intron1	C/T	I	T	T	T	T	T	T	T	C	T	T	T	T	T	T	T	T	C
**rs212173929**	75699721-75699720	Nfe2l2	4921-4922		intron1	GT del		GT																
**rs212191263**	75699708	Nfe2l2	4933		intron1	C/G	I	G	G	G	G	G	G	G	C	G	G	G	G	G	G	G	G	C
**rs213252200**	75699642-75699641	Nfe2l2	4999-5000		intron1	AT add		AA																
**rs245800454**	75699610	Nfe2l2	5031		intron1	C/T	I	C	C	C	C	C	C	C	T	C	C	C	C	C	C	T	T	T
**rs225803230**	75699581	Nfe2l2	5060		intron1	C/T	I	T	T	T	T	T	T	T	C	T	T	T	T	T	T	C	C	C
**rs27978333**	75699564	Nfe2l2	5077		intron1	C/T	I	T	T	T	T	T	T	T	C	T	T	T	T	T	T	C	C	C
**rs27978334**	75699534	Nfe2l2	5107		intron1	C/T	I	T	T	T	T	T	T	T	T	T	T	T	T	T	T	T	C	C
**rs235651349**	75699498	Nfe2l2	5143		intron1	C/T	I	T	T	T	T	T	T	T	T	T	T	T	T	T	T	T	C	T
**rs218247010**	75699408	Nfe2l2	5237		intron1	A del		A																
**rs264155648**	75699314	Nfe2l2	5327		intron1	G/T	I	G	G	G	G	G	G	G	T	G	G	G	G	G	G	T	T	T
**rs240662004**	75699303	Nfe2l2	5338		intron1	A/G	I	A	A	A	A	A	A	A	G	A	A	A	A	A	A	G	G	G
**rs224093309**	75699256	Nfe2l2	5385		intron1	A/T	I	T	T	T	T	T	T	T	A	T	T	T	T	T	T	T	T	A
**rs253664669**	75699213	Nfe2l2	5428		intron1	C/T	I	T	T	T	T	T	T	T	T	T	T	T	T	T	T	T	C	T
**rs238072552**	75699164	Nfe2l2	5477		intron1	C/T	I	T	T	T	T	T	T	T	C	T	T	T	T	T	T	T	T	T
**rs219076099**	75699130	Nfe2l2	5511		intron1	A/G	I	G	G	G	G	G	G	G	G	G	G	G	G	G	G	G	A	G
**rs263352895**	75699126	Nfe2l2	5515		intron1	A/G	I	A	A	A	A	A	A	A	A	A	A	A	A	A	A	A	G	A
**rs245184098**	75699125	Nfe2l2	5516		intron1	C/T	I	C	C	C	C	C	C	C	C	C	C	C	C	C	C	C	T	C
**rs227048157**	75699060	Nfe2l2	5581		intron1	A/G	I	G	G	G	G	G	G	G	A	G	G	G	G	G	G	A	G	G
**rs260138157**	75699051	Nfe2l2	5590		intron1	C/T	I	C	C	C	C	C	C	C	T	C	C	C	C	C	C	T	T	T
**rs230441864**	75699044	Nfe2l2	5597		intron1	G/T	I	G	G	G	G	G	G	G	T	G	G	G	G	G	G	T	T	T
**rs212252696**	75699001	Nfe2l2	5640		intron1	C/T	I	T	T	T	T	T	T	T	C	T	T	T	T	T	T	C	C	C
**rs252186577**	75698938	Nfe2l2	5703		intron1	A/G	I	A	A	A	A	A	A	A	G	A	A	A	A	A	A	A	A	G
**rs27978335**	75698906	Nfe2l2	5735		intron1	G/T	I	G	G	G	G	G	G	G	G	G	G	G	G	G	G	T	G	G
**rs216759747**	75698904	Nfe2l2	5737		intron1	A/C	I	A	A	A	A	A	A	A	A	A	A	A	A	A	A	A	C	A
**rs27978336**	75698898	Nfe2l2	5743		intron1	C/T	I	C	C	C	C	C	C	C	C	C	C	C	C	C	C	C	C	T
**rs234223309**	75698886	Nfe2l2	5755		intron1	A/G	I	A	A	A	A	A	A	A	A	A	A	A	A	A	A	A	G	A

ID	Ch2 bp Location	Gene	Distance (gene)	Distance (mRNA)	Region	Alleles (genome)	Consequence	C57BL/6J	129S1/SvImJ	A/J	AKR/J	BALB/cJ	C3H/HeJ	C57BL/6NJ	CAST/EiJ	CBA/J	DBA/2J	FVB/NJ	LP/J	NOD/ShiLtJ	NZO/HlLtJ	PWK/PhJ	SPRET/EiJ	WSB/EiJ
**rs27978337**	75698829	Nfe2l2	5812		intron1	A/T	I	A	A	A	A	A	A	A	A	A	A	A	A	A	A	T	A	A
**rs243339667**	75698823	Nfe2l2	5818		intron1	A/G	I	G	G	G	G	G	G	G	G	G	G	G	G	G	G	G	A	G
**rs223688217**	75698767	Nfe2l2	5874		intron1	G/T	I	T	T	T	T	T	T	T	T	T	T	T	T	T	T	G	G	T
**rs217407978**	75698713	Nfe2l2	5928		intron1	A/G	I	G	G	G	G	G	G	G	G	G	G	G	G	A	G	G	G	G
**rs244349460**	75698709	Nfe2l2	5932		intron1	A/G	I	G	G	G	G	G	G	G	G	G	G	G	G	G	G	A	G	G
**rs229858119**	75698699	Nfe2l2	5942		intron1	A/T	I	T	T	T	T	T	T	T	A	T	T	T	T	T	T	A	A	A
**rs263403778**	75698690	Nfe2l2	5951		intron1	A/G	I	A	A	A	A	A	A	A	A	A	A	A	A	A	A	G	G	A
**rs247837008**	75698657	Nfe2l2	5984		intron1	C/T	I	C	C	C	C	C	C	C	C	C	C	C	C	C	C	T	C	C
**rs226681843**	75698621	Nfe2l2	6020		intron1	A/G	I	G	G	G	G	G	G	G	G	G	G	G	G	G	G	A	A	G
**rs265907281**	75698613	Nfe2l2	6028		intron1	A/G	I	A	A	A	A	A	A	A	A	A	A	A	A	A	A	G	G	A
**rs237956040**	75698515	Nfe2l2	6126		intron1	C/T	I	T	T	T	T	T	T	T	C	T	T	T	T	T	T	T	C	C
**rs218020200**	75698509	Nfe2l2	6132		intron1	C/T	I	T	T	T	T	T	T	T	C	T	T	T	T	T	T	T	C	C
**rs252256218**	75698438	Nfe2l2	6203		intron1	A/G	I	G	G	G	G	G	G	G	A	G	G	G	G	G	G	G	A	A
**rs216531668**	75698418-75698417	Nfe2l2	6223-6224		intron1	TT del	I	TT																
**rs231963433**	75698416	Nfe2l2	6225		intron1	G/T	I	G																
**rs258160886**	75698347-75698346	Nfe2l2	6294-6295		intron1	A add	I	CA																
**rs29541228**	75698333	Nfe2l2	6308		intron1	C/G	I	G	C	C	G	C	C	G	C	C	C	G	C	C	C	C	C	C
**rs241106629**	75698328-75698327	Nfe2l2	6313-6314		intron1	CA del	I	CA																
**rs258719975**	75698284	Nfe2l2	6357		intron1	A/G	I	A	A	A	A	A	A	A	A	A	A	A	A	A	A	A	G	A
**rs243093852**	75698258	Nfe2l2	6383		intron1	A/C	I	C	C	C	C	C	C	C	C	C	C	C	C	C	C	C	C	A
**rs248986100**	75698230	Nfe2l2	6411		intron1	A del	I	A																
**rs218604721**	75698202	Nfe2l2	6439		intron1	A/G	I	A	A	A	A	A	A	A	A	A	A	A	A	A	A	A	G	A
**rs261249066**	75698183-75698182	Nfe2l2	6458-6459		intron1	AA del	I	AA																
**rs243395291**	75698143	Nfe2l2	6498		intron1	A/G	I	A	A	A	A	A	A	A	A	A	A	A	A	A	A	A	G	A
**rs27978338**	75698114	Nfe2l2	6527		intron1	A/G	I	A	A	A	A	A	A	A	G	A	A	A	A	A	A	G	G	G
**rs3688913**	75698104	Nfe2l2	6537		intron1	C/T	I	C	T	T	C	T	T	C	C	T	T	C	T	C	T	C	C	C
**rs27978339**	75698102	Nfe2l2	6539		intron1	A/C	I	C	C	C	C	C	C	C	C	C	C	C	C	C	C	A	C	C
**rs27978340**	75698098	Nfe2l2	6543		intron1	A/G	I	G	G	G	G	G	G	G	A	G	G	G	G	G	G	G	G	A
**rs252454188**	75698095-75698094	Nfe2l2	6546-6547		intron1	CA add	I	TT																
**rs249947002**	75698047	Nfe2l2	6594		intron1	A del	I	A																
**rs265643467**	75698032	Nfe2l2	6609		intron1	A/T	I	A	A	A	A	A	A	A	T	A	A	A	A	A	A	A	A	T
**rs251968117**	75698027	Nfe2l2	6614		intron1	A/C	I	C	C	C	C	C	C	C	A	C	C	C	C	C	C	C	C	A
**rs260277664**	75698013-75698011	Nfe2l2	6628-6629		intron1	AAC del	I	AAC																
**rs234553734**	75697966	Nfe2l2	6675		intron1	A/G	I	A	A	A	A	A	A	A	G	A	A	A	A	A	A	A	A	A
**rs246174753**	75697962	Nfe2l2	6679		intron1	T del	I	T																
**rs260634152**	75697897	Nfe2l2	6744		intron1	C/T	I	C	C	C	C	C	C	C	T	C	C	C	C	C	C	C	C	T
**rs25124205**	75697862	Nfe2l2	6779		intron1	G del	I	G																
**rs27978341**	75697860	Nfe2l2	6781		intron1	A/C	I	A																
**rs238021329**	75697829	Nfe2l2	6812		intron1	A/G	I	G	G	G	G	G	G	G	G	G	G	G	G	G	G	G	A	G
**rs27978342**	75697669	Nfe2l2	6972		intron1	A/G	I	A																
**rs233458866**	75697665-75697664	Nfe2l2	6976-6977		intron1	AG del	I	AG																
**rs218084461**	75697642	Nfe2l2	6999		intron1	A/T	I	T	T	T	T	T	T	T	T	T	T	T	T	T	T	T	A	T
**rs258213605**	75697583	Nfe2l2	7058		intron1	C/T	I	T	T	T	T	T	T	T	T	T	T	T	T	T	T	T	C	T
**rs215071073**	75697457-75697456	Nfe2l2	7184-7185		intron1	GAG add		TG																
**rs27978343**	75697438	Nfe2l2	7203		intron1	A/G	I	A	A	A	A	A	A	A	G	A	A	A	A	A	A	G	G	G
**rs225151570**	75697385	Nfe2l2	7256		intron1	C/T	I	T	T	T	T	T	T	T	T	T	T	T	T	T	T	T	C	T
**rs262978641**	75697369	Nfe2l2	7272		intron1	C/T	I	C	C	C	C	C	C	C	C	C	C	C	C	C	C	T	C	C
**rs240411269**	75697362	Nfe2l2	7279		intron1	A/T	I	A	A	A	A	A	A	A	A	A	A	A	A	A	A	A	T	A
**rs259918473**	75697257-75697256	Nfe2l2	7384-7385		intron1	A add	I	TA																
**rs226248995**	75697228	Nfe2l2	7413		intron1	A/G	I	G	G	G	G	G	G	G	G	G	G	G	G	G	G	G	A	G
**rs249316494**	75697174	Nfe2l2	7467		intron1	C/T	I	C	C	C	C	C	C	C	C	C	C	C	C	C	C	C	T	C
**rs230331568**	75697172	Nfe2l2	7469		intron1	C/T	I	C	C	C	C	C	C	C	C	C	C	C	C	C	C	C	C	T
**rs214723803**	75697160	Nfe2l2	7481		intron1	C/T	I	C	C	C	C	C	C	C	C	C	C	C	C	C	C	C	T	C
**rs244090892**	75697117	Nfe2l2	7524		intron1	C/T	I	T	T	T	T	T	T	T	C	T	T	T	T	T	T	T	T	T
**rs241381264**	75697051	Nfe2l2	7590		intron1	A/T	I	T	T	T	T	T	T	T	T	T	T	T	T	T	T	T	A	T
**rs216431792**	75697026	Nfe2l2	7615		intron1	A/G	I	G	G	G	G	G	G	G	G	G	G	G	G	G	G	A	A	G
**rs248783695**	75697010-75697009	Nfe2l2	7631-7632		intron1	T add	I	CT																
**rs241975610**	75696989	Nfe2l2	7652		intron1	A/G	I	G	G	G	G	G	G	G	A	G	G	G	G	A	G	A	A	A
**rs27978344**	75696875	Nfe2l2	7766		intron1	C/T	I	T	T	T	T	T	T	T	T	T	T	T	T	T	T	T	T	C
**rs216376541**	75696847	Nfe2l2	7794		intron1	A/G	I	G	G	G	G	G	G	G	G	G	G	G	G	G	G	G	A	G

ID	Ch2 bp Location	Gene	Distance (gene)	Distance (mRNA)	Region	Alleles (genome)	Consequence	C57BL/6J	129S1/SvImJ	A/J	AKR/J	BALB/cJ	C3H/HeJ	C57BL/6NJ	CAST/EiJ	CBA/J	DBA/2J	FVB/NJ	LP/J	NOD/ShiLtJ	NZO/HlLtJ	PWK/PhJ	SPRET/EiJ	WSB/EiJ
**rs244298852**	75696838	Nfe2l2	7803		intron1	C/T	I	C	C	C	C	C	C	C	C	C	C	C	C	C	C	T	C	C
**rs224190629**	75696765	Nfe2l2	7876		intron1	C/G	I	C	C	C	C	C	C	C	C	C	C	C	C	C	C	C	G	C
**rs258269796**	75696748	Nfe2l2	7893		intron1	C/G	I	C	C	C	C	C	C	C	C	C	C	C	C	C	C	C	G	C
**rs243006444**	75696657	Nfe2l2	7984		intron1	C/T	I	C	C	C	C	C	C	C	C	C	C	C	C	C	C	C	H	C
**rs264811980**	75696631-75696630	Nfe2l2	8010-8011		intron1	TA add	I	CT																
**rs232899007**	75696612	Nfe2l2	8029		intron1	C/T	I	C	C	C	C	C	C	C	C	C	C	C	C	C	C	C	C	T
**rs257816432**	75696576	Nfe2l2	8065		intron1	C del	I	C																
**rs261927881**	75696572	Nfe2l2	8069		intron1	G del	I	G																
**rs263280110**	75696572	Nfe2l2	8069		intron1	C/G/T	I	G	G	G	G	G	G	G	G	G	G	G	G	G	G	G	C	C
**rs251596967**	75696532	Nfe2l2	8109		intron1	A/C	I	A	A	A	A	A	A	A	A	A	A	A	A	A	A	A	C	A
**rs222045578**	75696530	Nfe2l2	8111		intron1	C/G	I	G	G	G	G	G	G	G	G	G	G	G	G	G	G	G	C	G
**rs248985673**	75696528	Nfe2l2	8113		intron1	C/T	I	T	T	T	T	T	T	T	C	T	T	T	T	T	T	T	T	T
**rs235896108**	75696526	Nfe2l2	8115		intron1	A/T	I	T	T	T	T	T	T	T	T	T	T	T	T	T	T	T	A	T
**rs220375206**	75696506	Nfe2l2	8135		intron1	C/T	I	C	C	C	C	C	C	C	T	C	C	C	C	C	C	C	T	C
**rs262193337**	75696386	Nfe2l2	8255		intron1	C/G	I	C	C	C	C	C	C	C	G	C	C	C	C	C	C	C	C	C
**rs238983207**	75696373	Nfe2l2	8268		intron1	C/T	I	C	C	C	C	C	C	C	C	C	C	C	C	C	C	T	C	C
**rs216486956**	75696310	Nfe2l2	8331		intron1	A/G	I	G	G	G	G	G	G	G	G	G	G	G	G	G	G	G	A	G
**rs27978345**	75696273	Nfe2l2	8368		intron1	A/G	I	A	A	A	A	A	A	A	A	A	A	A	A	A	A	G	A	A
**rs27978346**	75696143	Nfe2l2	8498		intron1	A/G	I	A	G	G	A	G	G	A	G	G	G	A	G	G	G	G	G	G
**rs216430126**	75696080	Nfe2l2	8561		intron1	C/T	I	C	C	C	C	C	C	C	C	C	C	C	C	C	C	C	T	C
**rs27978347**	75695985	Nfe2l2	8656		intron1	A/G	I	G	G	G	G	G	G	G	A	G	G	G	G	G	G	A	A	A
**rs249907617**	75695975-75695966	Nfe2l2	8666-8675		intron1	AGTAAAAGCT del	I	AGTAAAAGCT																
**rs223881123**	75695942	Nfe2l2	8699		intron1	C/T	I	T	T	T	T	T	T	T	T	T	T	T	T	T	T	T	T	C
**rs214394221**	75695929	Nfe2l2	8712		intron1	C/T	I	T	T	T	T	T	T	T	T	T	T	T	T	T	T	C	T	C
**rs252488612**	75695906-75695905	Nfe2l2	8735-8736		intron1	C add	I	AA																
**rs258528878**	75695859-75695858	Nfe2l2	8782-8783		intron1	CT add	I	AC																
**rs238660437**	75695839-75695838	Nfe2l2	8802-8803		intron1	AGG add	I	CA																
**rs255206844**	75695830	Nfe2l2	8811		intron1	C/G	I	C	C	C	C	C	C	C	C	C	C	C	C	C	C	C	G	C
**rs236252421**	75695792-75695791	Nfe2l2	8849-8850		intron1	TG add	I	CT																
**rs232287271**	75695700	Nfe2l2	8941		intron1	A/G	I	G	G	G	G	G	G	G	G	G	G	G	G	G	G	G	A	G
**rs265468575**	75695690	Nfe2l2	8951		intron1	C/T	I	C	C	C	C	C	C	C	C	C	C	C	C	C	C	T	C	C
**rs248579211**	75695665	Nfe2l2	8976		intron1	A/G	I	G	G	G	G	G	G	G	G	G	G	G	G	G	G	G	A	G
**rs27978348**	75695545	Nfe2l2	9096		intron1	C/T	I	C	C	C	C	C	C	C	T	C	C	C	C	C	C	T	T	T
**rs27978349**	75695521	Nfe2l2	9120		intron1	C/G	I	G	G	G	G	G	G	G	G	G	G	G	G	G	G	C	C	G
**rs235964997**	75695519	Nfe2l2	9122		intron1	A/G	I	G	G	G	G	G	G	G	G	G	G	G	G	G	G	A	G	G
**rs27978350**	75695449	Nfe2l2	9192		intron1	C/T	I	C	C	C	C	C	C	C	C	C	C	C	C	C	C	C	C	T
**rs257472311**	75695423	Nfe2l2	9218		intron1	A/G	I	A	A	A	A	A	A	A	A	A	A	A	A	A	A	A	G	A
**rs249601985**	75695417	Nfe2l2	9224		intron1	C/T	I	T	T	T	T	T	T	T	C	T	T	T	T	T	T	C	C	C
**rs224509750**	75695406	Nfe2l2	9235		intron1	A/G	I	**G**	G	G	G	G	G	G	G	G	G	G	G	G	G	G	A	A
**rs257252695**	75695382	Nfe2l2	9259		intron1	A/C/T	I	C	C	C	C	C	C	C	C	C	C	C	C	T	C	C	A	C
**rs235767822**	75695366	Nfe2l2	9275		intron1	A/G	I	A	A	A	A	A	A	A	G	A	A	A	A	A	A	A	G	G
**rs216485918**	75695365	Nfe2l2	9276		intron1	C/T	I	T	T	T	T	T	T	T	T	T	T	T	T	T	T	C	T	T
**rs250170344**	75695340	Nfe2l2	9301		intron1	C/T	I	C	C	C	C	C	C	C	T	C	C	C	C	C	C	C	C	C
**rs230843559**	75695335	Nfe2l2	9306		intron1	A/T	I	T	T	T	T	T	T	T	T	T	T	T	T	A	T	T	T	T
**rs27978351**	75695269	Nfe2l2	9372		intron1	C/T	I	T	T	T	T	T	T	T	C	T	T	T	T	T	T	T	C	T
**rs250724500**	75695228	Nfe2l2	9413		intron1	A/G	I	G	G	G	G	G	G	G	A	G	G	G	G	G	G	G	G	G
**rs241008850**	75695213	Nfe2l2	9428		intron1	A/G	I	G	G	G	G	G	G	G	A	G	G	G	G	G	G	G	G	G
**rs215652101**	75695212	Nfe2l2	9429		intron1	A/C	I	A	A	A	A	A	A	A	C	A	A	A	A	A	A	A	A	A
**rs241222487**	75695209-75695207	Nfe2l2	9432		intron1	CAA del	I	CAA																
**rs260039136**	75695047	Nfe2l2	9594		intron1	A/T	I	A	A	A	A	A	A	A	A	A	A	A	A	A	A	A	T	A
**rs27978352**	75695003	Nfe2l2	9638		intron1	A/T	I	T	T	T	T	T	T	T	A	T	T	T	T	T	T	A	A	T
**rs254796318**	75695000	Nfe2l2	9641		intron1	G/T	I	T	T	T	T	T	T	T	G	T	T	T	T	T	T	T	G	T
**rs242459063**	75694991	Nfe2l2	9650		intron1	A/G	I	A	A	A	A	A	A	A	G	A	A	A	A	A	A	G	G	G
**rs212978761**	75694939	Nfe2l2	9702		intron1	A del	I	A																
**rs27978354**	75694890	Nfe2l2	9751		intron1	C/T	I	T	T	T	T	T	T	T	C	T	T	T	T	T	T	T	C	T
**rs265090850**	75694844	Nfe2l2	9797		intron1	G/T	I	T	T	T	T	T	T	T	G	T	T	T	T	T	T	T	G	T
**rs246453127**	75694827	Nfe2l2	9814		intron1	C/T	I	T	T	T	T	T	T	T	C	T	T	T	T	T	T	T	C	T
**rs229452668**	75694808-15694807	Nfe2l2	9833-9834		intron1	T add	I	AA																
**rs240226829**	75694801	Nfe2l2	9840		intron1	A del	I	A																
**rs27978355**	75694792	Nfe2l2	9849		intron1	A/C	I	A	A	A	A	A	A	A	C	A	A	A	A	A	A	A	C	A
**rs224669038**	75694768	Nfe2l2	9873		intron1	C/T	I	C	C	C	C	C	C	C	C	C	C	C	C	C	C	C	T	C

ID	Ch2 bp Location	Gene	Distance (gene)	Distance (mRNA)	Region	Alleles (genome)	Consequence	C57BL/6J	129S1/SvImJ	A/J	AKR/J	BALB/cJ	C3H/HeJ	C57BL/6NJ	CAST/EiJ	CBA/J	DBA/2J	FVB/NJ	LP/J	NOD/ShiLtJ	NZO/HlLtJ	PWK/PhJ	SPRET/EiJ	WSB/EiJ
**rs264587050**	75694743	Nfe2l2	9898		intron1	G/T	I	G	G	G	G	G	G	G	G	G	G	G	G	G	G	G	T	G
**rs27978356**	75694729	Nfe2l2	9912		intron1	A/C/T	I	T	T	T	T	T	T	T	C	T	T	T	T	T	T	T	C	C
**rs221555322**	75694729-75694728	Nfe2l2	9912-9913		intron1	CGAAAAA add	I	GT																
**rs27978357**	75694694	Nfe2l2	9947		intron1	C/T	I	C	C	C	C	C	C	C	T	C	C	C	C	T	C	C	C	C
**rs27978358**	75694667	Nfe2l2	9974		intron1	C/T	I	T																
**rs27978359**	75694638	Nfe2l2	10003		intron1	C/T	I	C	C	C	C	C	C	C	C	C	C	C	C	T	C	C	C	C
**rs27978360**	75694600	Nfe2l2	10041		intron1	C/T	I	T	T	T	T	T	T	T	C	T	T	T	T	C	T	C	C	C
**rs223077967**	75694595	Nfe2l2	10046		intron1	A/G	I	A	A	A	A	A	A	A	G	A	A	A	A	A	A	A	A	A
**rs261833408**	75694584	Nfe2l2	10057		intron1	C/T	I	C	C	C	C	C	C	C	C	C	C	C	C	C	C	C	T	C
**rs27978361**	75694566	Nfe2l2	10075		intron1	C/T	I	T	T	T	T	T	T	T	C	T	T	T	T	C	T	C	C	C
**rs216165217**	75694473	Nfe2l2	10168		intron1	G/T	I	T	T	T	T	T	T	T	T	T	T	T	T	T	T	T	G	T
**rs247386358**	75694472	Nfe2l2	10169		intron1	G/T	I	T	T	T	T	T	T	T	T	T	T	T	T	T	T	T	G	T
**rs27978362**	75694251	Nfe2l2	10390		intron1	C/T	I	T	T	T	T	T	T	T	C	T	T	T	T	T	T	C	C	C
**rs212752209**	75694223	Nfe2l2	10418		intron1	A/C	I	A	A	A	A	A	A	A	A	A	A	A	A	A	A	A	C	A
**rs27978363**	75694211	Nfe2l2	10430		intron1	C/T	I	C	C	C	C	C	C	C	T	C	C	C	C	C	C	C	T	C
**rs226261680**	75694207	Nfe2l2	10434		intron1	C/T	I	T	T	T	T	T	T	T	T	T	T	T	T	T	T	T	C	T
**rs262437880**	75694183	Nfe2l2	10458		intron1	A/T	I	T	T	T	T	T	T	T	T	T	T	T	T	T	T	T	A	T
**rs258366972**	75694172	Nfe2l2	10469		intron1	A/G	I	A	A	A	A	A	A	A	A	A	A	A	A	A	A	A	G	A
**rs231692176**	75694122	Nfe2l2	10519		intron1	G/T	I	G	G	G	G	G	G	G	G	G	G	G	G	G	G	G	T	G
**rs216266719**	75694081-75694080	Nfe2l2	10560-10561		intron1	C add	I	GT																
**rs266250451**	75694011	Nfe2l2	10630		intron1	A/T	I	T	T	T	T	T	T	T	T	T	T	T	T	T	T	T	A	T
**rs234443827**	75693958-75693957	Nfe2l2	10683-10684		intron1	A add	I	CA																
**rs242345277**	75693946	Nfe2l2	10695		intron1	C/T	I	C	C	C	C	C	C	C	C	C	C	C	C	C	C	C	T	C
**rs219140592**	75693944	Nfe2l2	10697		intron1	A/C	I	A	A	A	A	A	A	A	A	A	A	A	A	A	A	A	C	A
**rs256280843**	75693890	Nfe2l2	10751		intron1	C/T	I	C	C	C	C	C	C	C	C	C	C	C	C	C	C	C	T	C
**rs239947873**	75693789	Nfe2l2	10852		intron1	C/T	I	C	C	C	C	C	C	C	C	C	C	C	C	C	C	C	T	C
**rs216899243**	75693738-75693737	Nfe2l2	10903-10904		intron1	ACAT add	I	CA																
**rs253908284**	75693582-75693581	Nfe2l2	11059-11060		intron1	TACTGA add	I	TT																
**rs222892149**	75693556	Nfe2l2	11085		intron1	A/G	I	A	A	A	A	A	A	A	A	A	A	A	A	A	A	A	G	A
**rs27978364**	75693380	Nfe2l2	11261		intron1	A/G	I	G	G	G	G	G	G	G	G	G	G	G	G	G	G	A	G	G
**rs27978365**	75693372	Nfe2l2	11269		intron1	A/T	I	T	T	T	T	T	T	T	T	T	T	T	T	T	T	T	T	A
**rs27978366**	75693312	Nfe2l2	11329		intron1	A/G	I	G	G	G	G	G	G	G	A	G	G	G	G	G	G	G	G	G
**rs253194149**	75693186	Nfe2l2	11455		intron1	C/T	I	C	C	C	C	C	C	C	C	C	C	C	C	C	C	C	T	C
**rs248381588**	75693050-75693049	Nfe2l2	11591-11592		intron1	A del	I	CA																
**rs234274465**	75693027	Nfe2l2	11614		intron1	A/G	I	G	G	G	G	G	G	G	G	G	G	G	G	G	G	G	A	G
**rs27978367**	75692900	Nfe2l2	11741		intron1	C/T	I	C	C	C	C	C	C	C	C	C	C	C	C	C	C	T	C	C
**rs27978368**	75692657	Nfe2l2	11984		intron1	A/G	I	G	G	G	G	G	G	G	A	G	G	G	G	G	G	G	G	G
**rs236837207**	75692603	Nfe2l2	12038		intron1	A/G	I	G	G	G	G	G	G	G	G	G	G	G	G	G	G	G	A	G
**rs253472269**	75692533-75692530	Nfe2l2	12108-12111		intron1	GACA del	I	GACA																
**rs214432552**	75692515	Nfe2l2	12126		intron1	A/G	I	G	G	G	G	G	G	G	A	G	G	G	G	G	G	G	G	G
**rs253307639**	75692484	Nfe2l2	12157		intron1	C/T	I	C	C	C	C	C	C	C	C	C	C	C	C	C	C	C	T	C
**rs27978369**	75692472	Nfe2l2	12169		intron1	G/T	I	G	G	G	G	G	G	G	G	G	G	G	G	G	G	T	G	G
**rs219269367**	75692415	Nfe2l2	12226		intron1	C/G	I	G	G	G	G	G	G	G	G	G	G	G	G	G	G	G	C	G
**rs248415591**	75692365	Nfe2l2	12276		intron1	C/T	I	C	C	C	C	C	C	C	C	C	C	C	C	C	C	C	T	C
**rs27978370**	75692325	Nfe2l2	12316		intron1	C/T	I	C	C	C	C	C	C	C	T	C	C	C	C	C	C	T	C	C
**rs256342429**	75692310	Nfe2l2	12331		intron1	C/G	I	C	C	C	C	C	C	C	C	C	C	C	C	C	C	C	G	C
**rs240013898**	75692302	Nfe2l2	12339		intron1	A/G	I	G	G	G	G	G	G	G	G	G	G	G	G	G	G	G	A	G
**rs230302480**	75692293	Nfe2l2	12348		intron1	A/G	I	G	G	G	G	G	G	G	G	G	G	G	G	G	G	G	A	G
**rs265019701**	75692285	Nfe2l2	12356		intron1	C/T	I	C	C	C	C	C	C	C	C	C	C	C	C	C	C	C	T	C
**rs246068622**	75692275	Nfe2l2	12366		intron1	A/T	I	T	T	T	T	T	T	T	T	T	T	T	T	T	T	T	A	T
**rs223836194**	75692266	Nfe2l2	12375		intron1	A/G	I	G	G	G	G	G	G	G	G	G	G	G	G	G	G	G	A	G
**rs252947323**	75692250	Nfe2l2	12391		intron1	C/T	I	C	C	C	C	C	C	C	C	C	C	C	C	C	C	C	T	C
**rs240469624**	75692225	Nfe2l2	12416		intron1	A/G	I	G	G	G	G	G	G	G	G	G	G	G	G	G	G	G	A	G
**rs218652579**	75692224	Nfe2l2	12417		intron1	C/T	I	T	T	T	T	T	T	T	T	T	T	T	T	T	T	T	C	T
**rs248125449**	75692216	Nfe2l2	12425		intron1	C/T	I	C	C	C	C	C	C	C	C	C	C	C	C	C	C	C	T	C
**rs238932953**	75692178	Nfe2l2	12463		intron1	C/T	I	C	C	C	C	C	C	C	C	C	C	C	C	C	C	C	T	C
**rs213599444**	75692103	Nfe2l2	12538		intron1	C/T	I	C	C	C	C	C	C	C	T	C	C	C	C	C	C	T	T	T
**rs263482071**	75692045	Nfe2l2	12596		intron1	C/G	I	C	C	C	C	C	C	C	C	C	C	C	C	C	C	C	G	C
**rs238050414**	75691947	Nfe2l2	12694		intron1	C/T	I	C	C	C	C	C	C	C	C	C	C	C	C	C	C	C	T	C
**rs217542350**	75691905	Nfe2l2	12736		intron1	G/T	I	T	T	T	T	T	T	T	T	T	T	T	T	T	T	T	G	T
**rs248100570**	75691839	Nfe2l2	12802		intron1	A/G	I	A	A	A	A	A	A	A	A	A	A	A	A	A	A	A	G	A
**rs225018335**	75691795	Nfe2l2	12846		intron1	A/G	I	A																
**rs212470148**	75691785	Nfe2l2	12856		intron1	A/G	I	G	G	G	G	G	G	G	G	G	G	G	G	G	G	G	A	G

ID	Ch2 bp Location	Gene	Distance (gene)	Distance (mRNA)	Region	Alleles (genome)	Consequence	C57BL/6J	129S1/SvImJ	A/J	AKR/J	BALB/cJ	C3H/HeJ	C57BL/6NJ	CAST/EiJ	CBA/J	DBA/2J	FVB/NJ	LP/J	NOD/ShiLtJ	NZO/HlLtJ	PWK/PhJ	SPRET/EiJ	WSB/EiJ
**rs251851993**	75691731	Nfe2l2	12910		intron1	G/T	I	T	T	T	T	T	T	T	T	T	T	T	T	T	T	T	G	T
**rs225391967**	75691546	Nfe2l2	13095		intron1	A/T	I	A																
**rs247407311**	75691539	Nfe2l2	13102		intron1	C del	I	C																
**rs27978371**	75691522	Nfe2l2	13119		intron1	C/G/T	I	C	C	C	C	C	C	C	T	C	C	C	C	C	C	T	G	T
**rs27978372**	75691510	Nfe2l2	13131		intron1	A/G	I	G	G	G	G	G	G	G	G	G	G	G	G	G	G	G	G	A
**rs248729212**	75691509	Nfe2l2	13132		intron1	C/T	I	C	C	C	C	C	C	C	C	C	C	C	C	C	C	C	T	C
**rs231456599**	75691475	Nfe2l2	13166		intron1	C/T	I	T	T	T	T	T	T	T	T	T	T	T	T	T	T	T	C	T
**rs259599891**	75691430	Nfe2l2	13211		intron1	C/T	I	T	T	T	T	T	T	T	T	T	T	T	T	T	T	T	C	T
**rs262868461**	75691406-75691402	Nfe2l2	13225-13229		intron1	AAGTC del	I	AAGTC																
**rs240535141**	75691380	Nfe2l2	13261		intron1	A/G	I	A	A	A	A	A	A	A	A	A	A	A	A	A	A	A	G	A
**rs218361012**	75691316	Nfe2l2	13325		intron1	A/C	I	C	C	C	C	C	C	C	C	C	C	C	C	C	C	C	A	C
**rs248173890**	75691263	Nfe2l2	13378		intron1	G/T	I	T	T	T	T	T	T	T	T	T	T	T	T	T	T	T	G	T
**rs244082281**	75691116	Nfe2l2	13525		intron1	A/G	I	G	G	G	G	G	G	G	G	G	G	G	G	G	G	G	A	G
**rs223115925**	75691014-75691013	Nfe2l2	13627-13628		intron1	T add	I	CT																
**rs222008407**	75690978	Nfe2l2	13663		intron1	C/T	I	C	C	C	C	C	C	C	C	C	C	C	C	C	C	C	T	C
**rs259325225**	75690963	Nfe2l2	13678		intron1	C/T	I	C	C	C	C	C	C	C	C	C	C	C	C	C	C	C	T	C
**rs239769129**	75690923	Nfe2l2	13718		intron1	C/T	I	C	C	C	C	C	C	C	C	C	C	C	C	C	C	C	T	C
**rs223640822**	75690918	Nfe2l2	13723		intron1	C/T	I	T	T	T	T	T	T	T	T	T	T	T	T	T	T	T	C	T
**rs27978373**	75690883	Nfe2l2	13758		intron1	A/T	I	A	A	A	A	A	A	A	T	A	A	A	A	A	A	T	T	T
**rs27978374**	75690785	Nfe2l2	13856		intron1	C/G	I	C	C	C	C	C	C	C	G	C	C	C	C	C	C	G	C	C
**rs258639503**	75690618-75690611	Nfe2l2	14023-14030		intron1	AAAAAAAA del	I	AAAAAAAA																
**rs218737303**	75690579-75690578	Nfe2l2	14062-14063		intron1	AAT add	I	AA																
**rs231565560**	75690578	Nfe2l2	14063		intron1	A del	I	A																
**rs212131694**	75690355	Nfe2l2	14286		intron1	G/T	I	T	T	T	T	T	T	T	T	T	T	T	T	T	T	T	G	T
**rs107692572**	75690309	Nfe2l2	14332		intron1	A/G	I	G	A	A	G	A	A	G	G	A	A	G	A	A	A	G	G	G
**rs236558948**	75690262	Nfe2l2	14379		intron1	G/T	I	G																
**rs217547750**	75690261	Nfe2l2	14380		intron1	A/T	I	T																
**rs240808293**	75690151-75690144	Nfe2l2	14490-14497		intron1	TGTTTGTT del	I	TGTTTGTT																
**5s211754330**	75690086	Nfe2l2	14555		intron1	T del	I	T																
**rs27978375**	75690058	Nfe2l2	14583		intron1	C/T	I	C	C	C	C	C	C	C	C	C	C	C	C	C	C	C	C	T
**rs229449638**	75690015	Nfe2l2	14626		intron1	C/T	I	C	C	C	C	C	C	C	C	C	C	C	C	C	C	C	T	C
**rs27978376**	75689979	Nfe2l2	14662		intron1	C/G	I	G	G	G	G	G	G	G	C	G	G	G	G	G	G	G	G	G
**rs246483703**	75689978	Nfe2l2	14663		intron1	C/G	I	G	G	G	G	G	G	G	G	G	G	G	G	G	G	G	C	G
**rs224018168**	75689970	Nfe2l2	14671		intron1	A/G	I	G	G	G	G	G	G	G	G	G	G	G	G	G	G	G	A	G
**rs261246510**	75689964	Nfe2l2	14677		intron1	C/T	I	C	C	C	C	C	C	C	C	C	C	C	C	C	C	C	T	C
**rs246452329**	75689947	Nfe2l2	14694		intron1	C/T	I	C	C	C	C	C	C	C	C	C	C	C	C	C	C	C	T	C
**rs233476474**	75689934	Nfe2l2	14707		intron1	C/T	I	T	T	T	T	T	T	T	C	T	T	T	T	T	T	C	C	C
**rs265025739**	75689927-75689925	Nfe2l2	14714		intron1	AC add	I	GA																
**rs27978377**	75689917	Nfe2l2	14724		intron1	A/C	I	A	A	A	A	A	A	A	C	A	A	A	A	A	A	A	A	A
**rs245096150**	75689875	Nfe2l2	14766		intron1	A/G	I	G	G	G	G	G	G	G	G	G	G	G	G	G	G	G	A	G
**rs27978378**	75689874	Nfe2l2	14767		intron1	C/G	I	C	C	C	C	C	C	C	C	C	C	C	C	C	C	G	C	C
**rs254640525**	75689731	Nfe2l2	14910		intron1	A/T	I	A	A	A	A	A	A	A	A	A	A	A	A	A	A	A	T	A
**rs27978379**	75689680	Nfe2l2	14961		intron1	C/T	I	T	T	T	T	T	T	T	C	T	T	T	T	T	T	C	T	T
**rs218266298**	75689621	Nfe2l2	15020		intron1	A/C	I	A	A	A	A	A	A	A	C	A	A	A	A	A	A	A	A	A
**rs259856667**	75689615	Nfe2l2	15026		intron1	T del		T																
**rs257944751**	75689600	Nfe2l2	15041		intron1	C/T	I	C	C	C	C	C	C	C	T	C	C	C	C	C	C	C	C	C
**rs238120833**	75689491	Nfe2l2	15150		intron1	C/T	I	T	T	T	T	T	T	T	T	T	T	T	T	T	T	T	C	T
**rs27978380**	75689484	Nfe2l2	15157		intron1	C/T	I	T	T	T	T	T	T	T	C	T	T	T	T	T	T	C	C	T
**rs257699286**	75689445	Nfe2l2	15196		intron1	C/T	I	T	T	T	T	T	T	T	T	T	T	T	T	T	T	T	C	T
**rs235456892**	75689306	Nfe2l2	15335		intron1	C/T	I	C	C	C	C	C	C	C	C	C	C	C	C	C	C	C	T	C
**rs211871995**	75689259-75689258	Nfe2l2	15382-15383		intron1	TG add	I	CT																
**rs27978381**	75689220	Nfe2l2	15421		intron1	A/G	I	A	A	A	A	A	A	A	G	A	A	A	A	A	A	A	A	A
**rs246534671**	75689175	Nfe2l2	15466		intron1	C/T	I	T	T	T	T	T	T	T	T	T	T	T	T	T	T	T	C	T
**rs242903958**	75689158-75689157	Nfe2l2	15483-15484		intron1	GTAGCTCCATGAG add	I	AT																
**rs224093477**	75689145	Nfe2l2	15496		intron1	A/G	I	A	A	A	A	A	A	A	G	A	A	A	A	A	A	G	G	G
**rs264522944**	75689072	Nfe2l2	15569		intron1	A/G	I	G	G	G	G	G	G	G	G	G	G	G	G	G	G	G	A	G
**rs251207380**	75689038	Nfe2l2	15603		intron1	C/T	I	T	T	T	T	T	T	T	T	T	T	T	T	T	T	T	C	T
**rs229770818**	75688962	Nfe2l2	15679		intron1	C/T	I	C	C	C	C	C	C	C	C	C	C	C	C	C	C	C	T	C
**rs27978382**	75688892	Nfe2l2	15749		intron1	A/G	I	G	G	G	G	G	G	G	G	G	G	G	G	G	G	A	G	G
**rs240489473**	75688891	Nfe2l2	15750		intron1	C/T	I	C	C	C	C	C	C	C	C	C	C	C	C	C	C	C	T	C
**rs223397427**	75688862	Nfe2l2	15779		intron1	C/T	I	T	T	T	T	T	T	T	T	T	T	T	T	T	T	T	C	T

ID	Ch2 bp Location	Gene	Distance (gene)	Distance (mRNA)	Region	Alleles (genome)	Consequence	C57BL/6J	129S1/SvImJ	A/J	AKR/J	BALB/cJ	C3H/HeJ	C57BL/6NJ	CAST/EiJ	CBA/J	DBA/2J	FVB/NJ	LP/J	NOD/ShiLtJ	NZO/HlLtJ	PWK/PhJ	SPRET/EiJ	WSB/EiJ
**rs260519058**	75688827	Nfe2l2	15814		intron1	C/T	I	C	C	C	C	C	C	C	C	C	C	C	C	C	C	C	T	C
**rs27978383**	75688826	Nfe2l2	15815		intron1	G/T	I	G	G	G	G	G	G	G	T	G	G	G	G	G	G	G	G	G
**rs218323665**	75688741	Nfe2l2	15900		intron1	A/G	I	G	G	G	G	G	G	G	A	G	G	G	G	G	G	G	A	G
**rs262531027**	75688720	Nfe2l2	15921		intron1	C/T	I	T	T	T	T	T	T	T	C	T	T	T	T	T	T	T	T	T
**rs237008118**	75688633-75688629	Nfe2l2	16008-16012		intron1	CACAA del	I	CACAA																
**rs243264033**	75688625	Nfe2l2	16016		intron1	C/G	I	C	C	C	C	C	C	C	G	C	C	C	C	C	C	C	C	C
**rs225285756**	75688562	Nfe2l2	16079		intron1	A/G	I	A	A	A	A	A	A	A	A	A	A	A	A	A	A	A	G	A
**rs263298220**	75688539	Nfe2l2	16102		intron1	G/T	I	T																
**rs27978384**	75688530	Nfe2l2	16111		intron1	A/G	I	G	G	G	G	G	G	G	G	G	G	G	G	A	G	G	G	G
**rs235506009**	75688521	Nfe2l2	16120		intron1	A/T	I	T	T	T	T	T	T	T	T	T	T	T	T	T	T	T	A	T
**rs222576029**	75688485	Nfe2l2	16156		intron1	T del	I	T																
**rs216967066**	75688484	Nfe2l2	16157		intron1	C/T	I	C	C	C	C	C	C	C	C	C	C	C	C	C	C	C	T	C
**rs249171752**	75688443	Nfe2l2	16198		intron1	C/T	I	T	T	T	T	T	T	T	T	T	T	T	T	T	T	T	C	T
**rs230212360**	75688434	Nfe2l2	16207		intron1	C/G	I	G	G	G	G	G	G	G	G	G	G	G	G	G	G	G	C	G
**rs211860287**	75688427	Nfe2l2	16214		intron1	A/G	I	A	A	A	A	A	A	A	G	A	A	A	A	A	A	G	G	A
**rs255977063**	75688382	Nfe2l2	16259		intron1	C/T	I	T	T	T	T	T	T	T	C	T	T	T	T	T	T	T	T	T
**rs229500580**	75688355	Nfe2l2	16286		intron1	A/G	I	G	G	G	G	G	G	G	A	G	G	G	G	G	G	G	G	G
**rs236656737**	75688342-75688341	Nfe2l2	16299-16300		intron1	G add	I	TG																
**rs234238255**	75688285-75688284	Nfe2l2	16355-16356		intron1	G add	I	AA																
**rs216769737**	75688236	Nfe2l2	16405		intron1	A/G	I	G	G	G	G	G	G	G	A	G	G	G	G	G	G	G	G	G
**rs246500082**	75688226	Nfe2l2	16415		intron1	A/G	I	G	G	G	G	G	G	G	G	G	G	G	G	G	G	G	A	G
**rs229208464**	75688214	Nfe2l2	16427		intron1	C/T	I	C	C	C	C	C	C	C	C	C	C	C	C	C	C	C	T	C
**rs29921147**	75688206	Nfe2l2	16435		intron1	C/T	I	T	C	C	T	C	C	T	C	C	C	T	C	C	C	C	C	C
**rs237960931**	75688205	Nfe2l2	16436		intron1	C/T	I	C	C	C	C	C	C	C	C	C	C	C	C	C	C	C	T	C
**rs227963693**	75688175	Nfe2l2	16466		intron1	A/G	I	G	G	G	G	G	G	G	G	G	G	G	G	G	G	G	A	G
**rs262877843**	75688143	Nfe2l2	16498		intron1	A/G	I	G	G	G	G	G	G	G	G	G	G	G	G	G	G	G	A	G
**rs246155572**	75688108	Nfe2l2	16533		intron1	G/T	I	T	T	T	T	T	T	T	G	T	T	T	T	T	T	T	G	T
**rs225089492**	75688088	Nfe2l2	16553		intron1	C/T	I	T	T	T	T	T	T	T	T	T	T	T	T	T	T	T	C	T
**rs218203558**	75688046-75688045	Nfe2l2	16595-16596		intron1	T add	I	GT																
**rs251298499**	75688038	Nfe2l2	16603		intron1	A/G	I	G	G	G	G	G	G	G	A	G	G	G	G	G	G	G	G	G
**rs241588688**	75688024	Nfe2l2	16617		intron1	G/T	I	T	T	T	T	T	T	T	T	T	T	T	T	T	T	T	G	T
**rs222301087**	75688004	Nfe2l2	16637		intron1	C/T	I	C	C	C	C	C	C	C	C	C	C	C	C	C	C	C	T	C
**rs215760764**	75688009-75687991	Nfe2l2	16642-16660		intron1	TCACCAGGTCTTACGGCTT del	I	TCACCAGGTC TTACGGCTT																
**rs249253594**	75687972	Nfe2l2	16669		intron1	C/T	I	C	C	C	C	C	C	C	C	C	C	C	C	C	C	C	T	C
**rs230276235**	75687900	Nfe2l2	16741		intron1	A/T	I	T	T	T	T	T	T	T	T	T	T	T	T	T	T	T	A	T
**rs27978385**	75687887	Nfe2l2	16754		intron1	C/G	I	C	C	C	C	C	C	C	G	C	C	C	C	C	C	C	C	C
**rs257687706**	75687879	Nfe2l2	16762		intron1	A/T	I	A	A	A	A	A	A	A	A	A	A	A	A	A	A	A	T	A
**rs237771825**	75687870	Nfe2l2	16771		intron1	C/T	I	C	C	C	C	C	C	C	T	C	C	C	C	C	C	C	C	C
**rs259393135**	75687843-75687842	Nfe2l2	16798-16799		intron1	TAG add	I	TT																
**rs216360636**	75687824	Nfe2l2	16817		intron1	C/T	I	C	C	C	C	C	C	C	C	C	C	C	C	C	C	C	T	C
**rs246170119**	75687768	Nfe2l2	16873		intron1	A/G	I	G	G	G	G	G	G	G	A	G	G	G	G	G	G	G	A	G
**rs219258971**	75687764	Nfe2l2	16877		intron1	A del	I	A																
**rs251636391**	75687698-75687696	Nfe2l2	16943-16945		intron1	TTG del	I	TTG																
**rs262585959**	756876965-75687695	Nfe2l2	16945-16946		intron1	TTGTTG add	I	TT																
**rs228911769**	75687677	Nfe2l2	16964		intron1	C/T	I	C	C	C	C	C	C	C	C	C	C	C	C	C	C	C	C	T
**rs27978386**	75687605	Nfe2l2	17036		intron1	A/G	I	G	G	G	G	G	G	G	G	G	G	G	G	G	G	G	G	A
**rs244238284**	75687600	Nfe2l2	17041		intron1	A/C	I	A	A	A	A	A	A	A	C	A	A	A	A	A	A	A	A	A
**rs27978387**	75687599	Nfe2l2	17042		intron1	C/T	I	T	T	T	T	T	T	T	T	T	T	T	T	T	T	C	T	T
**rs27978388**	75687544	Nfe2l2	17097		intron1	A/G	I	A	A	A	A	A	A	A	A	A	A	A	A	A	A	G	A	A
**rs217411560**	75687542-75687533	Nfe2l2	17099-17108		intron1	CACATGGGAA del	I	CACATGG GAA																
**rs242917439**	75687486	Nfe2l2	17155		intron1	C/T	I	T	T	T	T	T	T	T	C	T	T	T	T	T	T	C	C	T
**rs256353471**	75687476	Nfe2l2	17165		intron1	C del		C																
**rs233323092**	75687423	Nfe2l2	17218		intron1	A/T	I	A	A	A	A	A	A	A	A	A	A	A	A	A	A	A	T	A
**rs257385269**	75687421	Nfe2l2	17220		intron1	C/T	I	C	C	C	C	C	C	C	C	C	C	C	C	C	C	C	T	C
**rs241655620**	75687410	Nfe2l2	17231		intron1	A/G	I	G	G	G	G	G	G	G	G	G	G	G	G	G	G	G	A	G
**rs27978389**	75687364	Nfe2l2	17277		intron1	C/T	I	C	C	C	C	C	C	C	C	C	C	C	C	C	C	T	C	C
**rs255005538**	75687285	Nfe2l2	17356		intron1	A/G	I	G	G	G	G	G	G	G	G	G	G	G	G	G	G	A	G	G
**rs27978390**	75687218	Nfe2l2	17423		intron1	A/G	I	G	G	G	G	G	G	G	A	G	G	G	G	G	G	G	G	G
**rs27978391**	75687201	Nfe2l2	17440		intron1	A/T	I	A	A	A	A	A	A	A	A	A	A	A	A	A	A	T	A	A
**rs262159183**	75687176	Nfe2l2	17465		intron1	C/T	I	T	T	T	T	T	T	T	T	T	T	T	T	T	T	T	C	T

ID	Ch2 bp Location	Gene	Distance (gene)	Distance (mRNA)	Region	Alleles (genome)	Consequence	C57BL/6J	129S1/SvImJ	A/J	AKR/J	BALB/cJ	C3H/HeJ	C57BL/6NJ	CAST/EiJ	CBA/J	DBA/2J	FVB/NJ	LP/J	NOD/ShiLtJ	NZO/HlLtJ	PWK/PhJ	SPRET/EiJ	WSB/EiJ
**rs27978392**	75687133	Nfe2l2	17508		intron1	G/T	I	T	T	T	T	T	T	T	G	T	T	T	T	T	T	T	G	T
**rs260241376**	75687102	Nfe2l2	17539		intron1	C del	I	C																
**rs241531799**	75687098-75687088	Nfe2l2	17543-17553		intron1	AGCCACTAAGT del	I	AGCCACTAAGT																
**rs27978393**	75687089	Nfe2l2	17552		intron1	C/G	I	G																
**rs27978394**	75687074	Nfe2l2	17567		intron1	A/G	I	G	G	G	G	G	G	G	A	G	G	G	G	G	G	G	G	G
**rs252576568**	75687022	Nfe2l2	17619		intron1	A/G	I	A	A	A	A	A	A	A	G	A	A	A	A	A	A	G	G	A
**rs236094953**	75687021	Nfe2l2	17620		intron1	G/T	I	T	T	T	T	T	T	T	G	T	T	T	T	T	T	G	G	T
**rs234919878**	75686927	Nfe2l2	17714		intron1	A del	I	A																
**rs33657301**	75686766	Nfe2l2	17875		intron1	C/T	I	T	C	C	T	C	C	T	T	C	C	T	C	T	C	T	T	T
**rs6291081**	75686739	Nfe2l2	17902		intron1	A/C	I	A	A	A	A	A	A	A	A	A	A	A	A	A	A	C	A	A
**rs237257955**	75686729-75686728	Nfe2l2	17912-17913		intron1	GGT add	I	GG																
**rs250065181**	75686726	Nfe2l2	17915		intron1	G del	I	G																
**rs246201446**	75686726-75686725	Nfe2l2	17915-17916		intron1	GGGG add	I	TG																
**rs6290579**	75686681	Nfe2l2	17960		intron1	A/G	I	A	A	A	A	A	A	A	A	A	A	A	A	A	A	G	G	A
**rs236195686**	75686630	Nfe2l2	18011		intron1	A/G	I	G	G	G	G	G	G	G	A	G	G	G	G	G	G	G	G	G
**rs6289911**	75686506	Nfe2l2	18135		intron1	G/T	I	T	T	T	T	T	T	T	T	T	T	T	T	T	T	G	T	T
**rs6289504**	75686497	Nfe2l2	18144		intron1	A/T	I	A	A	A	A	A	A	A	A	A	A	A	A	A	A	T	T	A
**rs6289492**	75686488	Nfe2l2	18153		intron1	A/G	I	G	G	G	G	G	G	G	G	G	G	G	G	G	G	A	G	G
**rs257457265**	75686450	Nfe2l2	18191		intron1	G/T	I	T	T	T	T	T	T	T	T	T	T	T	T	T	T	T	G	T
**rs247603873**	75686394	Nfe2l2	18247		intron1	A/G	I	A	A	A	A	A	A	A	A	A	A	A	A	A	A	A	G	A
**rs29963020**	75686363	Nfe2l2	18278		intron1	A/G	I	A	G	G	A	G	G	A	G	G	G	A	G	G	G	A	G	G
**rs27978395**	75686356	Nfe2l2	18285		intron1	A/G	I	G	A	A	G	A	A	G	A	A	A	G	A	A	A	G	G	A
**rs241220935**	75686349	Nfe2l2	18292		intron1	C/T	I	C	C	C	C	C	C	C	T	C	C	C	C	C	C	C	C	C
**rs223623711**	75686347	Nfe2l2	18294		intron1	C/T	I	T	T	T	T	T	T	T	T	T	T	T	T	T	T	T	C	T
**rs262547165**	75686321	Nfe2l2	18320		intron1	A/C	I	A	A	A	A	A	A	A	A	A	A	A	A	A	A	A	C	A
**rs249506237**	75686294	Nfe2l2	18347		intron1	C/T	I	C	C	C	C	C	C	C	C	C	C	C	C	C	C	C	T	C
**rs221242453**	75686269	Nfe2l2	18372		intron1	C/G	I	C	C	C	C	C	C	C	C	C	C	C	C	C	C	C	G	C
**rs27978396**	75686196	Nfe2l2	18445		intron1	C/G	I	G																
**rs263951189**	75686194-75686193	Nfe2l2	18447-18448		intron1	CA add	I	TC																
**rs252619336**	75686142	Nfe2l2	18499		intron1	C/T	I	C	C	C	C	C	C	C	C	C	C	C	C	C	C	C	T	C
**rs27978397**	75686131	Nfe2l2	18510		intron1	G/T	I	G	G	G	G	G	G	G	G	G	G	G	G	G	G	T	G	G
**rs27978398**	75686130	Nfe2l2	18511		intron1	C/T	I	C	T	T	C	T	T	C	C	T	T	C	T	T	T	C	C	T
**rs27978399**	75686107	Nfe2l2	18534		intron1	C/T	I	T	T	T	T	T	T	T	C	T	T	T	T	T	T	T	C	T
**rs234289601**	75686047	Nfe2l2	18594		intron1	A/T	I	A	A	A	A	A	A	A	A	A	A	A	A	A	A	A	T	A
**rs27978400**	75686020	Nfe2l2	18621		intron1	A/G	I	G	G	G	G	G	G	G	G	G	G	G	G	A	G	G	G	G
**rs250663244**	75685898	Nfe2l2	18743		intron1	C/T	I	T	T	T	T	T	T	T	T	T	T	T	T	T	T	T	C	T
**rs27978401**	75685785	Nfe2l2	18856		intron1	C/T	I	T	T	T	T	T	T	T	C	T	T	T	T	T	T	T	T	T
**rs215062303**	75685744	Nfe2l2	18897		intron1	A/G	I	G	G	G	G	G	G	G	G	G	G	G	G	G	G	G	A	G
**rs27978402**	75685668	Nfe2l2	18973		intron1	C/T	I	C	C	C	C	C	C	C	C	C	C	C	C	C	C	T	T	C
**rs27978403**	75685644	Nfe2l2	18997		intron1	C/T	I	C																
**rs228658200**	75685574	Nfe2l2	19067		intron1	A/G	I	G	G	G	G	G	G	G	G	G	G	G	G	G	G	G	A	G
**rs254740588**	75685568	Nfe2l2	19073		intron1	C/T	I	T	T	T	T	T	T	T	T	T	T	T	T	T	T	T	C	T
**rs252770560**	75685551	Nfe2l2	19090		intron1	A/G	I	A	A	A	A	A	A	A	A	A	A	A	A	A	A	A	G	A
**rs27978404**	75685507	Nfe2l2	19134		intron1	A/G	I	A	A	A	A	A	A	A	G	A	A	A	A	A	A	G	G	A
**rs265179807**	75685504	Nfe2l2	19137		intron1	C/T	I	C	C	C	C	C	C	C	C	C	C	C	C	C	C	C	T	C
**rs27978405**	75685454	Nfe2l2	19187		intron1	C/T	I	C	C	C	C	C	C	C	C	C	C	C	C	C	C	T	T	C
**rs27978406**	75685447	Nfe2l2	19194		intron1	G/T	I	T	T	T	T	T	T	T	G	T	T	T	T	T	T	T	T	T
**rs261488043**	75685354	Nfe2l2	19287		intron1	C/T	I	C	C	C	C	C	C	C	C	C	C	C	C	C	C	C	T	C
**rs242223826**	75685345	Nfe2l2	19296		intron1	A/G	I	G	G	G	G	G	G	G	G	G	G	G	G	G	G	G	A	G
**rs27978407**	75685330	Nfe2l2	19311		intron1	C/T	I	C	C	C	C	C	C	C	C	C	C	C	C	T	C	C	C	C
**rs27978408**	75685263	Nfe2l2	19378		intron1	A/G	I	A	A	A	A	A	A	A	A	A	A	A	A	G	A	A	A	A
**rs243646204**	75685219	Nfe2l2	19422		intron1	C/T	I	T	T	T	T	T	T	T	C	T	T	T	T	T	T	C	T	T
**rs223008439**	75684985	Nfe2l2	19656		intron1	A/G	I	G	G	G	G	G	G	G	G	G	G	G	G	G	G	G	A	G
**rs27978409**	75684829	Nfe2l2	19812		intron1	C/T	I	C	C	C	C	C	C	C	T	C	C	C	C	C	C	T	T	C
**rs233552476**	75684808	Nfe2l2	19833		intron1	A/G	I	A	A	A	A	A	A	A	A	A	A	A	A	A	A	A	G	A
**rs27978410**	75684768	Nfe2l2	19873		intron1	A/G	I	A	G	G	A	G	G	A	A	G	G	A	G	G	G	G	G	G
**rs232536970**	75684771-75684766	Nfe2l2	19876-19881		intron1	GAAGGA del	I	GAAGGA																
**rs253088076**	75684670	Nfe2l2	19971		intron1	C/T	I	T	T	T	T	T	T	T	T	T	T	T	T	T	T	T	C	T
**rs220539340**	75684680-75684666	Nfe2l2	19981-19995		intron1	TGCCTAAGTATTTTT del	I	TGCCTAAGTATTTTT																
**rs223272915**	75684593-75684592	Nfe2l2	20048-20049		intron1	A add	I	CC																
**rs27978411**	75684559	Nfe2l2	20082		intron1	A/G	I	A	A	A	A	A	A	A	G	A	A	A	A	A	A	G	G	A
**rs213492116**	75684550	Nfe2l2	20091		intron1	C/T	I	C	C	C	C	C	C	C	C	C	C	C	C	C	C	C	T	C

ID	Ch2 bp Location	Gene	Distance (gene)	Distance (mRNA)	Region	Alleles (genome)	Consequence	C57BL/6J	129S1/SvImJ	A/J	AKR/J	BALB/cJ	C3H/HeJ	C57BL/6NJ	CAST/EiJ	CBA/J	DBA/2J	FVB/NJ	LP/J	NOD/ShiLtJ	NZO/HlLtJ	PWK/PhJ	SPRET/EiJ	WSB/EiJ
**rs249243149**	75684540	Nfe2l2	20101		intron1	A/T	I	A	A	A	A	A	A	A	A	A	A	A	A	A	A	A	T	A
**rs231310013**	75684537	Nfe2l2	20104		intron1	A/G	I	G	G	G	G	G	G	G	A	G	G	G	G	G	G	G	G	G
**rs213860116**	75684509	Nfe2l2	20132		intron1	C/T	I	C	C	C	C	C	C	C	T	C	C	C	C	C	C	C	C	C
**rs258282522**	75684413	Nfe2l2	20228		intron1	C/T	I	T	T	T	T	T	T	T	C	T	T	T	T	T	T	C	C	T
**rs227740832**	75684382	Nfe2l2	20259		intron1	A/C	I	A	A	A	A	A	A	A	A	A	A	A	A	A	A	C	A	A
**rs246641702**	75684365	Nfe2l2	20276		intron1	A del	I	A																
**rs27978412**	75684333	Nfe2l2	20308		intron1	C/T	I	C	C	C	C	C	C	C	T	C	C	C	C	C	C	T	T	T
**rs242285058**	75684321	Nfe2l2	20320		intron1	C/T	I	T	T	T	T	T	T	T	C	T	T	T	T	T	T	C	C	T
**rs215035587**	75684303-75684302	Nfe2l2	20338-20339		intron1	AAAAA add	I	CA																
**rs27978413**	75684276	Nfe2l2	20365		intron1	C/T	I	C	C	C	C	C	C	C	T	C	C	C	C	C	C	C	C	C
**rs264812265**	75684213	Nfe2l2	20428		intron1	A/G	I	G	G	G	G	G	G	G	G	G	G	G	G	G	G	G	A	G
**rs240899800**	75684181	Nfe2l2	20460		intron1	G/T	I	T	T	T	T	T	T	T	T	T	T	T	T	T	T	T	G	T
**rs222806418**	75684034	Nfe2l2	20607		intron1	A/C/G	I	G	G	G	G	G	G	G	C	G	G	G	G	G	G	G	A	G
**rs256771833**	75683993	Nfe2l2	20648		intron1	A/C	I	A	A	A	A	A	A	A	A	A	A	A	A	A	A	A	C	A
**rs239663884**	75683894	Nfe2l2	20747		intron1	G/T	I	T																
**rs257753830**	75683894	Nfe2l2	20747		intron1	T del	I	T																
**rs218082599**	75683893	Nfe2l2	20748		intron1	G/T	I	G																
**rs220746248**	75683892	Nfe2l2	20749		intron1	G/T	I	G																
**rs263505398**	75683892-75683891	Nfe2l2	20749-20750		intron1	TG del	I	TG																
**rs235365209**	75683892-75683884	Nfe2l2	20749-20757		intron1	TTTTTTTG del	I	TTTTTTTG																
**rs253138419**	75683876	Nfe2l2	20765		intron1	G/T	I	T																
**rs244355406**	75683874-75683869	Nfe2l2	20767		intron1	TTTTTT del	I	TTTTTT																
**rs234208943**	75683818	Nfe2l2	20823		intron1	C/T	I	T	T	T	T	T	T	T	C	T	T	T	T	T	T	C	C	T
**rs212690251**	75683653	Nfe2l2	20988		intron1	A/G	I	G	G	G	G	G	G	G	G	G	G	G	G	G	G	G	A	G
**rs261069701**	75683574	Nfe2l2	21067		intron1	C/T	I	C	C	C	C	C	C	C	C	C	C	C	C	C	C	C	T	C
**rs237174768**	75683543	Nfe2l2	21098		intron1	A/G	I	G	G	G	G	G	G	G	G	G	G	G	G	G	G	G	A	G
**rs214351399**	75683541	Nfe2l2	21100		intron1	A/T	I	A	A	A	A	A	A	A	A	A	A	A	A	A	A	A	T	A
**rs247325037**	75683512	Nfe2l2	21129		intron1	A/G	I	A	A	A	A	A	A	A	A	A	A	A	A	A	A	A	G	A
**rs233974636**	75683511	Nfe2l2	21130		intron1	C/T	I	C	C	C	C	C	C	C	C	C	C	C	C	C	C	C	T	C
**rs217804255**	75683501	Nfe2l2	21140		intron1	C/T	I	C	C	C	C	C	C	C	C	C	C	C	C	C	C	C	T	C
**rs27978414**	75683485	Nfe2l2	21156		intron1	C/T	I	C	T	T	C	T	T	C	C	T	T	C	T	C	T	C	C	C
**rs225276876**	75683451	Nfe2l2	21190		intron1	C/T	I	C	C	C	C	C	C	C	C	C	C	C	C	C	C	C	T	C
**rs219519006**	75683424-75683421	Nfe2l2	21217-21220		intron1	GGTA del	I	GGTA																
**rs262319437**	75683346-75683345	Nfe2l2	21295-21296		intron1	GG add	I	CG																
**rs232690739**	75683337-75683336	Nfe2l2	21304-21305		intron1	AC del	I	AC																
**rs261677569**	75683307	Nfe2l2	21334		intron1	A/G	I	G	G	G	G	G	G	G	G	G	G	G	G	G	G	G	A	G
**rs256546172**	75683291	Nfe2l2	21350		intron1	C/T	I	C	C	C	C	C	C	C	C	C	C	C	C	C	C	C	T	C
**rs27978415**	75683284	Nfe2l2	21357		intron1	C/T	I	C	C	C	C	C	C	C	T	C	C	C	C	C	C	C	C	C
**rs27978416**	75683271	Nfe2l2	21370		intron1	C/G	I	G	G	G	G	G	G	G	G	G	G	G	G	G	G	G	G	C
**rs239721304**	75683236	Nfe2l2	21405		intron1	A/G	I	G	G	G	G	G	G	G	G	G	G	G	G	G	G	G	A	G
**rs220440401**	75683214	Nfe2l2	21427		intron1	A/G	I	G	G	G	G	G	G	G	G	G	G	G	G	G	G	G	A	G
**rs27978417**	75683187	Nfe2l2	21454		intron1	A/C	I	C	C	C	C	C	C	C	C	C	C	C	C	C	C	A	C	C
**rs27978418**	75683181	Nfe2l2	21460		intron1	C/T	I	C	C	C	C	C	C	C	C	C	C	C	C	T	C	C	C	C
**rs220980711**	75683136	Nfe2l2	21505		intron1	C/T	I	T	T	T	T	T	T	T	T	T	T	T	T	T	T	T	C	T
**rs255387847**	75683125	Nfe2l2	21516		intron1	A/G	I	G	G	G	G	G	G	G	A	G	G	G	G	G	G	G	G	G
**rs238894116**	75683047	Nfe2l2	21594		intron1	C/T	I	C	C	C	C	C	C	C	C	C	C	C	C	C	C	C	T	C
**rs225062191**	75683039	Nfe2l2	21602		intron1	A/G	I	A																
**rs27978419**	75683036	Nfe2l2	21605		intron1	C/G	I	G	G	G	G	G	G	G	G	G	G	G	G	G	G	C	G	G
**rs27978420**	75682955	Nfe2l2	21686		intron1	A/G	I	G	G	G	G	G	G	G	G	G	G	G	G	G	G	G	G	A
**rs234059247**	75682946	Nfe2l2	21695		intron1	G/T	I	T	T	T	T	T	T	T	T	T	T	T	T	T	T	T	G	T
**rs228940386**	75682904-75682903	Nfe2l2	21737-21738		intron1	GT del	I	GT																
**rs217482709**	75682811	Nfe2l2	21830		intron1	A/C	I	A	A	A	A	A	A	A	A	A	A	A	A	A	A	A	C	A
**rs248417948**	75682718	Nfe2l2	21923		intron1	A/G	I	A	A	A	A	A	A	A	A	A	A	A	A	A	A	G	A	A
**rs235798440**	75682688	Nfe2l2	21953		intron1	A/G	I	A	A	A	A	A	A	A	A	A	A	A	A	A	A	A	G	A
**rs212667806**	75682659	Nfe2l2	21982		intron1	A/C	I	C	C	C	C	C	C	C	C	C	C	C	C	C	C	C	C	A
**rs33331598**	75682634	Nfe2l2	22007		intron1	A/T	I	A	T	T	A	T	T	A	A	T	T	A	T	A	T	T	A	A
**rs29674908**	75682633	Nfe2l2	22008		intron1	A/T	I	A	T	T	A	T	T	A	A	T	T	A	T	T	T	T	A	A
**rs218520528**	75682625	Nfe2l2	22016		intron1	G del	I	G																
**rs33392658**	75682625	Nfe2l2	22016		intron1	G/T	I	G	T	T	G	T	T	G	G	T	T	G	T	T	T	T	T	G
**rs33069533**	75682582	Nfe2l2	22059		intron1	A/G	I	A	G	G	A	G	G	A	G	G	G	A	G	G	G	G	G	A
**rs226574641**	75682515	Nfe2l2	22126		intron1	A/G	I	A	A	A	A	A	A	A	A	A	A	A	A	A	A	A	G	A

ID	Ch2 bp Location	Gene	Distance (gene)	Distance (mRNA)	Region	Alleles (genome)	Consequence	C57BL/6J	129S1/SvImJ	A/J	AKR/J	BALB/cJ	C3H/HeJ	C57BL/6NJ	CAST/EiJ	CBA/J	DBA/2J	FVB/NJ	LP/J	NOD/ShiLtJ	NZO/HlLtJ	PWK/PhJ	SPRET/EiJ	WSB/EiJ
**rs247845240**	75682419-75682418	Nfe2l2	22222-22223		intron1	TA del	I	TA																
**rs263152656**	75682405-75682403	Nfe2l2	22236-22238		intron1	TTT del	I	TTT																
**rs249354677**	75682403-75682402	Nfe2l2	22238-22239		intron1	TTTTT add	I	CT																
**rs229062009**	75682384-75682383	Nfe2l2	22257-22258		intron1	C add	I	TC																
**rs259536965**	75682374	Nfe2l2	22267		intron1	A/G	I	G	G	G	G	G	G	G	G	G	G	G	G	G	G	G	G	A
**rs232802991**	75682354	Nfe2l2	22287		intron1	A/G	I	A	A	A	A	A	A	A	A	A	A	A	A	A	A	A	A	G
**rs254858925**	75682337-75682336	Nfe2l2	22304-22305		intron1	T add	I	AT																
**rs32935671**	75682302	Nfe2l2	22339		intron1	A/C	I	C	A	A	C	A	A	C	C	A	A	C	A	C	A	C	C	C
**rs264637599**	75682282	Nfe2l2	22359		intron1	C/T	I	C	C	C	C	C	C	C	C	C	C	C	C	C	C	C	T	C
**rs219603117**	75682234-75682233	Nfe2l2	22407-22408		intron1	T add	I	AT																
**rs32858611**	75682191	Nfe2l2	22450		intron1	A/G	I	A	G	G	A	G	G	A	G	G	G	A	G	G	G	G	G	G
**rs221938247**	75682093	Nfe2l2	22548		intron1	A/G	I	A	A	A	A	A	A	A	A	A	A	A	A	A	A	A	G	A
**rs219190709**	75681989-75681988	Nfe2l2	22652-22653		intron1	TTTA add	I	TA																
**rs231775532**	75681977-75681976	Nfe2l2	22664-22665		intron1	TTTTTTTT add	I	CT																
**rs253805492**	75681925	Nfe2l2	22716		intron1	A/G	I	A	A	A	A	A	A	A	A	A	A	A	A	A	A	A	G	A
**rs234151932**	75681904	Nfe2l2	22737		intron1	C/T	I	C	C	C	C	C	C	C	C	C	C	C	C	C	C	C	T	C
**rs27978421**	75681886	Nfe2l2	22755		intron1	A/T	I	T	T	T	T	T	T	T	T	T	T	T	T	T	T	T	T	A
**rs27978422**	75681849	Nfe2l2	22792		intron1	C/T	I	T	T	T	T	T	T	T	C	T	T	T	T	T	T	C	C	T
**rs218555017**	75681814-75681812	Nfe2l2	22827-22829		intron1	TAG del	I	TAG																
**rs237320475**	75681786	Nfe2l2	22855		intron1	C/T	I	C	C	C	C	C	C	C	C	C	C	C	C	C	C	C	T	C
**rs212421595**	75681785	Nfe2l2	22856		intron1	C/T	I	C	C	C	C	C	C	C	C	C	C	C	C	C	C	C	T	C
**rs262651526**	75681771	Nfe2l2	22870		intron1	A/C/T	I	T	T	T	T	T	T	T	C	T	T	T	T	T	T	T	A	T
**rs253619508**	75681713-75681703	Nfe2l2	22928-22938		intron1	AAAAAGTAAAA del	I	AAAAAGTAAAA																
**rs243753654**	75681635-75681620	Nfe2l2	23006-23021		intron1	ACACACACACACACAC del	I	ACACACACACACACAC																
**rs52263804**	75681580	Nfe2l2	23061		intron1	C/T	I	T	C	C	T	C	C	T	T	C	C	T	C	T	C	T	T	T
**rs216180461**	75681542	Nfe2l2	23099		intron1	A/G	I	G	G	G	G	G	G	G	G	G	G	G	G	G	G	G	A	G
**rs245473635**	75681540	Nfe2l2	23101		intron1	C/T	I	C	C	C	C	C	C	C	C	C	C	C	C	C	C	C	T	C
**rs27978423**	75681459	Nfe2l2	23182		intron1	A/G/T	I	A	A	A	A	A	A	A	G	A	A	A	A	A	A	T	A	A
**rs27978424**	75681420	Nfe2l2	23221		intron1	C/T	I	T	T	T	T	T	T	T	C	T	T	T	T	T	T	T	T	T
**rs253643368**	75681402	Nfe2l2	23239		intron1	A/G	I	G	G	G	G	G	G	G	G	G	G	G	G	G	G	G	A	G
**rs27978425**	75681399	Nfe2l2	23242		intron1	C/T	I	C	C	C	C	C	C	C	C	C	C	C	C	C	C	C	C	T
**rs254672551**	75681365-75681364	Nfe2l2	23276-23277		intron1	C add	I	TC																
**rs33117644**	75681306	Nfe2l2	23335		intron1	C/T	I	C	T	T	C	T	T	C	C	T	T	C	T	T	T	C	C	C
**rs243356759**	75681242-75681238	Nfe2l2	23399-23403		intron1	TGGGT del	I	TGGGT																
**rs246830545**	75681170	Nfe2l2	23471		intron1	A/G	I	G	G	G	G	G	G	G	G	G	G	G	G	G	G	G	A	G
**rs225685586**	75681123	Nfe2l2	23518		intron1	A/G	I	G	G	G	G	G	G	G	G	G	G	G	G	G	G	G	A	G
**rs259796141**	75681073	Nfe2l2	23568		intron1	A/G	I	G	G	G	G	G	G	G	G	G	G	G	G	G	G	G	A	G
**rs240359404**	75681058	Nfe2l2	23583		intron1	A/C	I	C	C	C	C	C	C	C	C	C	C	C	C	C	C	C	A	C
**rs224404301**	75681025-75681026	Nfe2l2	23616-23617		intron1	TT del	I	TT																
**rs223639296**	75680973	Nfe2l2	23668		intron1	A/G	I	G	G	G	G	G	G	G	G	G	G	G	G	G	G	G	A	G
**rs27978426**	75680965	Nfe2l2	23676		intron1	C/T	I	C	C	C	C	C	C	C	C	C	C	C	C	T	C	C	C	C
**rs27978427**	75680962	Nfe2l2	23679		intron1	C/T	I	C	C	C	C	C	C	C	C	C	C	C	C	C	C	T	C	C
**rs220186933**	75680959	Nfe2l2	23682		intron1	A/G	I	G	G	G	G	G	G	G	G	G	G	G	G	G	G	G	A	G
**rs258265207**	75680955	Nfe2l2	23686		intron1	C/T	I	C	C	C	C	C	C	C	C	C	C	C	C	C	C	T	C	C
**rs27978428**	75680920	Nfe2l2	23721		intron1	A/G	I	G	G	G	G	G	G	G	G	G	G	G	G	G	G	A	A	G
**rs216239109**	75680838	Nfe2l2	23803		intron1	A/G	I	A	A	A	A	A	A	A	G	A	A	A	A	A	A	A	A	A
**rs251491229**	75680806	Nfe2l2	23835		intron1	C/T	I	T	T	T	T	T	T	T	T	T	T	T	T	T	T	T	C	T
**rs235405984**	75680790	Nfe2l2	23851		intron1	C/T	I	C	C	C	C	C	C	C	C	C	C	C	C	C	C	T	T	C
**rs217182304**	75680786	Nfe2l2	23855		intron1	C/T	I	T	T	T	T	T	T	T	T	T	T	T	T	T	T	C	C	T
**rs258936346**	75680771	Nfe2l2	23870		intron1	A/T	I	T	T	T	T	T	T	T	T	T	T	T	T	T	T	T	A	T
**rs27978429**	75680657	Nfe2l2	23984		intron1	C/T	I	T	T	T	T	T	T	T	T	T	T	T	T	T	T	C	T	T
**rs211759178**	75680648	Nfe2l2	23993		intron1	A/G	I	A	A	A	A	A	A	A	A	A	A	A	A	A	A	A	G	A
**rs251129306**	75680535	Nfe2l2	24106		intron1	A/C	I	A	A	A	A	A	A	A	A	A	A	A	A	A	A	A	C	A
**rs27978430**	75680531	Nfe2l2	24110		intron1	C/T	I	C	T	T	C	T	T	C	C	T	T	C	T	T	T	C	C	C
**rs27978431**	75680379	Nfe2l2	24262		intron1	A/T	I	A	T	T	A	T	T	A	T	T	T	A	T	A	T	T	T	T
**rs27978432**	75680340	Nfe2l2	24301		intron1	A/C	I	A	A	A	A	A	A	A	A	A	A	A	A	A	A	C	A	A

ID	Ch2 bp Location	Gene	Distance (gene)	Distance (mRNA)	Region	Alleles (genome)	Consequence	C57BL/6J	129S1/SvImJ	A/J	AKR/J	BALB/cJ	C3H/HeJ	C57BL/6NJ	CAST/EiJ	CBA/J	DBA/2J	FVB/NJ	LP/J	NOD/ShiLtJ	NZO/HlLtJ	PWK/PhJ	SPRET/EiJ	WSB/EiJ
**rs229099039**	75680317	Nfe2l2	24324		intron1	C/T	I	T	T	T	T	T	T	T	T	T	T	T	T	T	T	T	C	T
**rs264128136**	75680215	Nfe2l2	24426		intron1	C/G	I	G	G	G	G	G	G	G	G	G	G	G	G	G	G	G	C	G
**rs245222883**	75680214	Nfe2l2	24427		intron1	C/T	I	T	T	T	T	T	T	T	T	T	T	T	T	T	T	T	C	T
**rs220566279**	75680155	Nfe2l2	24486		intron1	A/G	I	G	G	G	G	G	G	G	G	G	G	G	G	G	G	G	A	G
**rs262495243**	75680125	Nfe2l2	24516		intron1	C/T	I	T	T	T	T	T	T	T	T	T	T	T	T	T	T	T	C	T
**rs243198614**	75680033	Nfe2l2	24608		intron1	C/T	I	C	C	C	C	C	C	C	C	C	C	C	C	C	C	C	T	C
**rs221698110**	75680013	Nfe2l2	24628		intron1	C/G	I	C	C	C	C	C	C	C	C	C	C	C	C	C	C	C	G	C
**rs251546337**	75679986	Nfe2l2	24655		intron1	A/G	I	G	G	G	G	G	G	G	G	G	G	G	G	G	G	A	A	G
**rs220372806**	75679977-75679967	Nfe2l2	24664-24674		intron1	CAAATAAAAAA del	I	CAAATAAAAAA																
**rs235456838**	75679968	Nfe2l2	24673		intron1	A/G	I	A	A	A	A	A	A	A	A	A	A	A	A	A	A	A	G	A
**rs222177937**	75679966	Nfe2l2	24675		intron1	C/G	I	G	G	G	G	G	G	G	G	G	G	G	G	G	G	G	C	G
**rs27978433**	75679945	Nfe2l2	24696		intron1	C/T	I	T	C	C	T	C	C	T	T	C	C	T	C	T	C	T	T	T
**rs236978645**	75679909	Nfe2l2	24732		intron1	A/C	I	C	C	C	C	C	C	C	C	C	C	C	C	C	C	C	A	C
**rs216777294**	75679899-75679898	Nfe2l2	24742-24743		intron1	AG del	I	AG																
**rs257522941**	75679887-75679884	Nfe2l2	24754-24757		intron1	CCCG del	I	CCCG																
**rs252158258**	75679887-75679883	Nfe2l2	24754-24758		intron1	CCCCG del	I	CCCCG																
**rs261072869**	75679887-75679881	Nfe2l2	24754-24760		intron1	CCCCCCG del	I	CCCCCCG																
**rs211801942**	75679876	Nfe2l2	24765		intron1	C/G	I	C																
**rs249628159**	75679876-75679875	Nfe2l2	24765-24767		intron1	CC del	I	CC																
**rs255894738**	75679858	Nfe2l2	24783		intron1	A/G	I	G	G	G	G	G	G	G	G	G	G	G	G	G	G	A	G	G
**rs231235581**	75679843	Nfe2l2	24798		intron1	A/G	I	G	G	G	G	G	G	G	G	G	G	G	G	G	G	G	A	G
**rs215914381**	75679808	Nfe2l2	24833		intron1	A/G	I	G	G	G	G	G	G	G	G	G	G	G	G	G	G	A	G	G
**rs253915353**	75679707	Nfe2l2	24934		intron1	T del	I	T																
**rs246427849**	75679705	Nfe2l2	24936		intron1	C/T	I	C	C	C	C	C	C	C	C	C	C	C	C	C	C	C	T	C
**rs250359732**	75679705-75679704	Nfe2l2	24936-24937		intron1	T add	I	CC																
**rs229152145**	75679703	Nfe2l2	24938		intron1	C/G	I	C	C	C	C	C	C	C	C	C	C	C	C	C	C	C	G	C
**rs218274481**	75679698-75679697	Nfe2l2	24943-24944		intron1	C add	I	TC																
**rs265891073**	75679697	Nfe2l2	24944		intron1	C/T	I	T	T	T	T	T	T	T	T	T	T	T	T	T	T	C	C	T
**rs249724035**	75679639	Nfe2l2	25002		intron1	A/T	I	A	A	A	A	A	A	A	A	A	A	A	A	A	A	A	T	A
**rs27978434**	75679636	Nfe2l2	25005		intron1	A/G	I	G	G	G	G	G	G	G	G	G	G	G	G	A	G	G	G	G
**rs262846659**	75679627	Nfe2l2	25014		intron1	C/T	I	C	C	C	C	C	C	C	C	C	C	C	C	C	C	C	T	C
**rs27978435**	75679598	Nfe2l2	25043		intron1	C/T	I	T	T	T	T	T	T	T	T	T	T	T	T	T	T	C	T	T
**rs13460861**	75679262	Nfe2l2	25379	446	exon2	A/G	Cns F71L	A	G	G	A	G	G	A	G	G	G	A	G	A	G	G	G	G
**rs13460859**	75679202	Nfe2l2	25439	506	exon2	C/T	Cs H91H	G	A	A	G	A	A	G	G	A	A	G	A	G	A	G	G	G
**rs27978436**	75679187	Nfe2l2	25454	521	exon2	G/A*	Cs T96=	G	G	G	G	G	G	G	A	G	G	G	G	G	G	A	G	G
**rs226131070**	75679178	Nfe2l2	25463	530	exon2	A/G	Cs S99=	G	G	G	G	G	G	G	G	G	G	G	G	G	G	G	A	G
**rs27978437**	75679151	Nfe2l2	25490		intron2	C/G	I	C	C	C	C	C	C	C	G	C	C	C	C	C	C	G	G	C
**rs234919466**	75679141	Nfe2l2	25500		intron2	A/G	I	G	G	G	G	G	G	G	A	G	G	G	G	G	G	G	G	G
**rs27978438**	75679104	Nfe2l2	25537		intron2	A/C	I	C	C	C	C	C	C	C	C	C	C	C	C	C	C	A	A	C
**rs257621708**	75679101	Nfe2l2	25540		intron2	C/G	I	C	C	C	C	C	C	C	C	C	C	C	C	C	C	C	G	C
**rs108910930**	75679064	Nfe2l2	25577		intron2	C/T	I	C	C	C	C	C	C	C	T	C	C	C	C	C	C	T	C	C
**rs259195219**	75679061-75979059	Nfe2l2	25580-25582		intron2	CTC del	I	CTC																
**rs215602300**	75679044	Nfe2l2	25597		intron2	G/T	I	G	G	G	G	G	G	G	T	G	G	G	G	G	G	T	T	G
**rs27978439**	75678977	Nfe2l2	25664		intron2	A/C	I	A	A	A	A	A	A	A	C	A	A	A	A	A	A	C	C	A
**rs27978440**	75678965	Nfe2l2	25676		intron2	C/T	I	T	C	C	T	C	C	T	C	C	C	T	C	T	C	C	C	C
**rs217925599**	75678887	Nfe2l2	25754		intron2	C/T	I	T	T	T	T	T	T	T	T	T	T	T	T	T	T	T	C	T
**rs27978441**	75678826	Nfe2l2	25815		intron2	A/G	I	G	A	A	G	A	A	G	A	A	A	G	A	G	A	A	G	G
**rs254710041**	75678807-75678804	Nfe2l2	25834-25837		intron2	TTTC del	I	TTTC																
**rs227483570**	75678798	Nfe2l2	25843		intron2	C/T	I	C	C	C	C	C	C	C	C	C	C	C	C	C	C	C	T	C
**rs258979414**	75678747	Nfe2l2	25894		intron2	C/T		C																
**rs258691522**	75678715-75678714	Nfe2l2	25926-25927		intron2	TTAC add	I	AT																
**rs27978442**	75678677	Nfe2l2	25964		intron2	C/T	I	T	C	C	T	C	C	T	C	C	C	T	C	C	C	C	C	C
**rs27978443**	75678639	Nfe2l2	26002		intron2	A/G	I	A	A	A	A	A	A	A	G	A	A	A	A	A	A	G	G	A
**rs227683834**	75678576	Nfe2l2	26065	547	exon2	A/T	Cns V105D	A	A	A	A	A	A	A	A	A	A	A	A	A	A	A	T	A
**rs257321187**	75678506	Nfe2l2	26135	617	exon2	C/T	Cs P128=	T	T	T	T	T	T	T	C	T	T	T	T	T	T	T	T	T
**rs27978444**	75678500	Nfe2l2	26141	623	exon2	A/T	Cs V130=	T	T	T	T	T	T	T	A	T	T	T	T	T	T	A	T	T
**rs27978445**	75678465	Nfe2l2	26176		intron3	C/T	I	C	C	C	C	C	C	C	T	C	C	C	C	C	C	T	C	C

ID	Ch2 bp Location	Gene	Distance (gene)	Distance (mRNA)	Region	Alleles (genome)	Consequence	C57BL/6J	129S1/SvImJ	A/J	AKR/J	BALB/cJ	C3H/HeJ	C57BL/6NJ	CAST/EiJ	CBA/J	DBA/2J	FVB/NJ	LP/J	NOD/ShiLtJ	NZO/HlLtJ	PWK/PhJ	SPRET/EiJ	WSB/EiJ
**rs256294642**	75678386-<br>75678385	Nfe2l2	24255-<br>24256		intron3	T add	I	AT																
**rs27978446**	75678346	Nfe2l2	26295		intron3	A/G	I	A	A	A	A	A	A	A	A	A	A	A	A	G	A	A	A	A
**rs244398374**	75678272	Nfe2l2	26369		intron3	C/T	I	C	C	C	C	C	C	C	T	C	C	C	C	T	C	T	C	C
**rs220047705**	75678130	Nfe2l2	26511		intron3	T del		T																
**rs239003075**	75678130-<br>75678129	Nfe2l2	26511-<br>26512		intron3	TTTT add		AT																
**rs223748107**	75678129	Nfe2l2	26512		intron3	A/T	I	A	A	A	A	A	A	A	A	A	A	A	A	T	A	A	A	A
**rs242153116**	75678129	Nfe2l2	26512		intron3	A del		A																
**rs251839690**	75678121	Nfe2l2	26520		intron3	C/T	I	C	C	C	C	C	C	C	C	C	C	C	C	C	C	T	C	C
**rs29502945**	75678047	Nfe2l2	26594		intron3	C/T	I	C	T	T	C	T	T	C	C	T	T	C	T	C	T	C	C	C
**rs237817856**	75678045-<br>75678044	Nfe2l2	26596-<br>26597		intron3	TAC add		AT																
**rs14620377**	75677988	Nfe2l2	26653		intron3	T del		T																
**rs221540999**	75677962	Nfe2l2	26679		intron3	A/G	I	A	A	A	A	A	A	A	A	A	A	A	A	A	A	A	G	A
**rs27978447**	75677902	Nfe2l2	26739		intron3	C/T	I	C	C	C	C	C	C	C	T	C	C	C	C	C	C	T	T	C
**rs236044343**	75677793	Nfe2l2	26848		intron3	C/T	I	C	C	C	C	C	C	C	C	C	C	C	C	C	C	T	C	C
**rs27978448**	75677781	Nfe2l2	26860		intron3	A/T	I	A	A	A	A	A	A	A	A	A	A	A	A	A	A	T	T	A
**rs27978449**	75677770	Nfe2l2	26871		intron3	A/G	I	A	G	G	A	G	G	A	G	G	G	A	G	G	G	G	G	G
**rs236117794**	75677761	Nfe2l2	26880		intron3	G/T	I	T	T	T	T	T	T	T	G	T	T	T	T	T	T	T	T	T
**rs214621680**	75677733	Nfe2l2	26908		intron3	C/G	I	G	G	G	G	G	G	G	G	G	G	G	G	G	G	G	C	G
**rs27978450**	75677721	Nfe2l2	26920		intron3	C/T	I	C	C	C	C	C	C	C	C	C	C	C	C	C	C	C	C	T
**rs227743136**	75677686	Nfe2l2	26955	662	exon4	A/G	Cs H143=	G	G	G	G	G	G	G	G	G	G	G	G	G	G	G	A	G
**rs215327202**	75677664	Nfe2l2	26977	684	exon4	C/G	Cns A151P	C	C	C	C	C	C	C	G	C	C	C	C	C	C	C	C	C
**rs247539755**	75677663	Nfe2l2	26978	685	exon4	A/G	Cns A151V	G	G	G	G	G	G	G	A	G	G	G	G	G	G	G	G	G
**rs233164668**	75677640	Nfe2l2	27001	708	exon4	C/T	Cns N159D	T	T	T	T	T	T	T	T	T	T	T	T	T	T	T	C	T
**rs27978451**	75677437	Nfe2l2	27204		intron4	A/G	I	G	A	A	G	A	A	G	G	A	A	G	A	G	A	G	G	G
**rs241652525**	75677244	Nfe2l2	27397		intron4	A/C	I	A	A	A	A	A	A	A	A	A	A	A	A	A	A	A	C	A
**rs231556518**	75677189	Nfe2l2	27452		intron4	A/G	I	G	G	G	G	G	G	G	G	G	G	G	G	G	G	G	A	G
**rs234095816**	75677162-<br>75677160	Nfe2l2	27479-<br>27481	826-828	exon5	GCT del	Cds-indel<br>(Q198)	GCT																
**rs27978452**	75677145	Nfe2l2	27496	843	exon5	C/T	Cns A204T	C	C	C	C	C	C	C	C	C	C	C	C	C	C	C	C	T
**rs225047937**	75677086-<br>75677078	Nfe2l2	27555-<br>27563	902-908	exon5	GAGATCGAT del	Cds-indel<br>(S223)	GAGATCG<br>AT																
**rs241469868**	75676998	Nfe2l2	27643	990	exon5	A/T	Cn T253S	T	T	T	T	T	T	T	T	T	T	T	T	T	T	T	A	T
**rs221602571**	75676923	Nfe2l2	27718	1065	exon5	A/C	Cns S278A	A	A	A	A	A	A	A	A	A	A	A	A	A	A	A	C	A
**rs252576472**	75676876	Nfe2l2	27765	1112	exon5	A/G	Cs S293=	G	G	G	G	G	G	G	G	G	G	G	G	G	G	G	A	G
**rs250802933**	75676841	Nfe2l2	27800	1147	exon5	C/T	Cns D305G	T	T	T	T	T	T	T	T	T	T	T	T	T	T	T	C	T
**rs225425698**	75676792	Nfe2l2	27849	1196	exon5	C/T	Cs P321=	C	C	C	C	C	C	C	C	C	C	C	C	C	C	C	T	C
**rs13459064**	75676732	Nfe2l2	27909	1256	exon5	C/T	Cs T341=	C	T	T	C	T	T	C	T	T	T	C	T	C	T	T	C	C
**rs27978453**	75676717	Nfe2l2	27924	1271	exon5	C/T	Cs A346=	C	C	C	C	C	C	C	C	C	C	C	C	C	C	T	C	C
**rs214335034**	75676636	Nfe2l2	28005	1352	exon5	A/G	Cs D373=	A	A	A	A	A	A	A	A	A	A	A	A	A	A	A	G	A
**rs249583644**	75676603	Nfe2l2	28038	1385	exon5	A/C	Cs P384=	A	A	A	A	A	A	A	A	A	A	A	A	A	A	A	C	A
**rs27978454**	75676592	Nfe2l2	28049	1396	exon5	A/G	Cns P388L	G	G	G	G	G	G	G	G	G	G	G	G	G	G	A	G	G
**rs215384328**	75676589	Nfe2l2	28052	1399	exon5	C/G	Cns A389G	G	G	G	G	G	G	G	G	G	G	G	G	G	G	G	C	G
**rs251728286**	75676571	Nfe2l2	28070	1417	exon5	A/G	Cns T395I	G																
**rs247602334**	75676567	Nfe2l2	28074	1421	exon5	C/T	Cs V396=	T	T	T	T	T	T	T	T	T	T	T	T	T	T	T	C	T
**rs234216231**	75676530	Nfe2l2	28111	1458	exon5	C/T	Cns M409V	T	T	T	T	T	T	T	T	T	T	T	T	T	T	T	C	T
**rs212904337**	75676526	Nfe2l2	28115	1462	exon5	C/T	Cns R410H	C	C	C	C	C	C	C	C	C	C	C	C	C	C	C	T	C
**rs252650779**	75676522	Nfe2l2	28119	1466	exon5	C/T	Cs E411=	T	T	T	T	T	T	T	T	T	T	T	T	T	T	T	C	T
**rs231273560**	75676516	Nfe2l2	28125	1472	exon5	C/T	Cs Q413=	T	T	T	T	T	T	T	T	T	T	T	T	T	T	T	C	T
**rs257886949**	75676413	Nfe2l2	28228	1575	exon5	G/T	Cs R448=	G	G	G	G	G	G	G	G	G	G	G	G	G	G	G	T	G
**rs241537608**	75676404	Nfe2l2	28237	1584	exon5	C/G	Cns L451V	G	G	G	G	G	G	G	G	G	G	G	G	G	G	G	C	G
**rs227619071**	75676312	Nfe2l2	28329	1676	exon5	C/T	Cs Q481=	T	T	T	T	T	T	T	T	T	T	T	T	T	T	T	C	T
**rs258913831**	75676290	Nfe2l2	28351	1698	exon5	A/G	Cs L489=	A	A	A	A	A	A	A	A	A	A	A	A	A	A	A	G	A
**rs225593319**	75676126	Nfe2l2	28515	1862	exon5	A/T	Cns H543Q	A																
**rs4223233**	75676105	Nfe2l2	28536	1883	exon5	A/G	Cs S550=	G	G	G	G	G	G	G	A	G	G	G	G	G	G	G	G	G
**rs4223232**	75676032	Nfe2l2	28609	1956	exon5	G/T	Cns L575M	G																
**rs4223231**	75675816	Nfe2l2	28825	2172	UTR-3	C/G		G	G	G	G	G	G	G	G	G	G	G	G	G	G	G	C	G
**rs1346860**	75675682	Nfe2l2	28959	2306	UTR-3	A/T		A																
**rs261783170**	75675500		19		3'flanking	A/T		A	A	A	A	A	A	A	A	A	A	A	A	A	A	T	A	A
**rs243572591**	75675499		20		3'flanking	C/T		C	C	C	C	C	C	C	C	C	C	C	C	C	C	T	C	C
**rs220033230**	75675486		33		3'flanking	G/T		G	G	G	G	G	G	G	G	G	G	G	G	G	G	G	T	G
**rs249647265**	75675455		64		3'flanking	C/T		T	T	T	T	T	T	T	T	T	T	T	T	T	T	T	C	T
**rs233494850**	75675392		127		3'flanking	C/G/T		C	C	C	C	C	C	C	C	C	C	C	C	C	C	C	T	C
**rs216601494**	75675386-<br>75675385		133-134		3'flanking	CTGC add		AC																
**rs220635722**	75675382		137		3'flanking	A/T		T	T	T	T	T	T	T	T	T	T	T	T	T	T	T	A	T
**rs264343181**	75675367		152		3'flanking	C/T		C	C	C	C	C	C	C	C	C	C	C	C	C	C	C	T	C

ID	Ch2 bp Location	Gene	Distance (gene)	Distance (mRNA)	Region	Alleles (genome)	Consequence	C57BL/6J	129S1/SvImJ	A/J	AKR/J	BALB/cJ	C3H/HeJ	C57BL/6NJ	CAST/EiJ	CBA/J	DBA/2J	FVB/NJ	LP/J	NOD/ShiLtJ	NZO/HlLtJ	PWK/PhJ	SPRET/EiJ	WSB/EiJ
**rs239674834**	75675342		177		3'flanking	C/T		C	C	C	C	C	C	C	C	C	C	C	C	C	C	C	T	C
**rs213419141**	75675334		185		3'flanking	C/T		C	C	C	C	C	C	C	C	C	C	C	C	C	C	T	T	C
**rs249190426**	75675331		188		3'flanking	A/G		A	A	A	A	A	A	A	A	A	A	A	A	A	A	A	G	A
**rs232907814**	75675324		195		3'flanking	G/T		G	G	G	G	G	G	G	G	G	G	G	G	T	G	G	G	G
**rs214021885**	75675323		196		3'flanking	C/T		C	C	C	C	C	C	C	C	C	C	C	C	C	C	C	T	C
**rs48178164**	75675303		216		3'flanking	A/G		A	G	G	A	G	G	A	G	G	G	A	G	G	G	G	G	G
**rs227681967**	75675294		225		3'flanking	C/T		C	C	C	C	C	C	C	C	C	C	C	C	C	C	C	T	C
**rs261486797**	75675285		234		3'flanking	G/T		T	T	T	T	T	T	T	G	T	T	T	T	T	T	G	T	T
**rs259443848**	75675278		241		3'flanking	A/G		G	G	G	G	G	G	G	G	G	G	G	G	G	G	G	A	G
**rs232922866**	75675264		255		3'flanking	C/T		T	T	T	T	T	T	T	T	T	T	T	T	T	T	T	C	T
**rs264795377**	75675263		256		3'flanking	C/T		C	C	C	C	C	C	C	C	C	C	C	C	C	C	C	T	C
**rs240835090**	75675187		332		3'flanking	A/G		G	G	G	G	G	G	G	G	G	G	G	G	G	G	G	A	G
**rs219770048**	75675183		336		3'flanking	A/G		A	A	A	A	A	A	A	A	A	A	A	A	A	A	A	G	A
**rs265363464**	75675114		405		3'flanking	C del		C																
**rs255005507**	75675103		416		3'flanking	G/T		G																
**rs33428762**	75675093		426		3'flanking	A/T		T	A	A	T	A	A	T	T	A	A	T	A	A	A	T	T	A
**rs239999563**	75674955		564		3'flanking	C/T		C	C	C	C	C	C	C	C	C	C	C	C	C	C	C	T	C
**rs220693620**	75674944		575		3'flanking	C/T		T	T	T	T	T	T	T	T	T	T	T	T	T	T	C	T	T
**rs260798971**	75674892		627		3'flanking	A/C		C	C	C	C	C	C	C	C	C	C	C	C	C	C	C	A	C
**rs241893311**	75674886		633		3'flanking	C/T		C	C	C	C	C	C	C	C	C	C	C	C	C	C	C	T	C
**rs27978455**	75674852		667		3'flanking	A/G		G	G	G	G	G	G	G	G	G	G	G	G	G	G	A	G	G
**rs261018791**	75674803		716		3'flanking	C/T		C	C	C	C	C	C	C	C	C	C	C	C	C	C	C	T	C
**rs27978456**	75674741		778		3'flanking	C/T		C	C	C	C	C	C	C	T	C	C	C	C	C	C	C	T	C
**rs213718140**	75674715		804		3'flanking	A/C		A	A	A	A	A	A	A	C	A	A	A	A	A	A	A	A	A
**rs247267701**	75674707		812		3'flanking	A/G		A	A	A	A	A	A	A	G	A	A	A	A	A	A	A	A	A
**rs32940094**	75674558		961		3'flanking	A/G		G	A	A	G	A	A	G	G	A	A	G	A	A	A	G	G	A
**rs229553908**	75674504		1015		3'flanking	A/G		G	G	G	G	G	G	G	G	G	G	G	G	G	G	G	A	G
**rs261847312**	75674488		1031		3'flanking	A/G		A	A	A	A	A	A	A	G	A	A	A	A	A	A	A	A	A
**rs27978457**	75674452		1067		3'flanking	A/T		A	A	A	A	A	A	A	T	A	A	A	A	A	A	A	T	A
**rs255074765**	75674352		1167		3'flanking	A/C		A	A	A	A	A	A	A	A	A	A	A	A	A	A	C	A	A
**rs226837531**	75674343		1176		3'flanking	C/T		C	C	C	C	C	C	C	T	C	C	C	C	C	C	C	C	C
**rs266125361**	75674318		1201		3'flanking	A/G		A	A	A	A	A	A	A	A	A	A	A	A	A	A	G	A	A
**rs247345565**	75674238		1281		3'flanking	C/T		C	C	C	C	C	C	C	C	C	C	C	C	C	C	C	T	C
**rs220900392**	75674222		1297		3'flanking	C/T		C	C	C	C	C	C	C	T	C	C	C	C	C	C	C	C	C
**rs255704871**	75674195		1324		3'flanking	A/G		A	A	A	A	A	A	A	A	A	A	A	A	A	A	A	G	A
**rs27978458**	75674158		1361		3'flanking	C/T		C	T	T	C	T	T	C	C	T	T	C	T	T	T	C	C	T
**rs27978459**	75674098		1421		3'flanking	C/T		T	C	C	T	C	C	T	C	C	C	T	C	C	C	C	C	C
**rs29717614**	75674091		1428		3'flanking	C/T		C	T	T	C	T	T	C	T	T	T	C	T	T	T	T	T	T
**rs233974698**	75674088		1431		3'flanking	C/T		T	T	T	T	T	T	T	T	T	T	T	T	T	T	T	C	T
**rs219797696**	75674026		1493		3'flanking	C/T		T	T	T	T	T	T	T	C	T	T	T	T	T	T	T	C	T
**rs27978460**	75674006		1513		3'flanking	C/T		T	T	T	T	T	T	T	C	T	T	T	T	T	T	C	C	T
**rs27978461**	75673995		1524		3'flanking	G/T		T	T	T	T	T	T	T	G	T	T	T	T	T	T	G	G	T
**rs212589942**	75673978		1541		3'flanking	A/T		A	A	A	A	A	A	A	T	A	A	A	A	A	A	T	T	A
**rs27978462**	75673968		1551		3'flanking	A/G		A	A	A	A	A	A	A	G	A	A	A	A	A	A	A	G	A
**rs27978463**	75673849		1670		3'flanking	A/G		A	A	A	A	A	A	A	G	A	A	A	A	A	A	A	G	A
**rs27978464**	75673846		1673		3'flanking	A/T		T	T	T	T	T	T	T	A	T	T	T	T	T	T	A	A	T
**rs27978465**	75673640		1879		3'flanking	A/G		G	G	G	G	G	G	G	A	G	G	G	G	G	G	G	G	G
**rs27978466**	75673591		1928		3'flanking	A/C		C	C	C	C	C	C	C	C	C	C	C	C	C	C	A	C	C
**rs264435069**	75673581		1938		3'flanking	A/G		G	G	G	G	G	G	G	G	G	G	G	G	G	G	G	A	G
**rs27978467**	75673572		1947		3'flanking	A/G		G	G	G	G	G	G	G	A	G	G	G	G	G	G	G	G	G
**rs27993468**	75673571		1948		3'flanking	C/T		C	C	C	C	C	C	C	C	C	C	C	C	C	C	T	C	C
**rs264610028**	75673539		1980		3'flanking	A/T		A	A	A	A	A	A	A	A	A	A	A	A	A	A	A	T	A

Cobmined database from MPD and NCBI dbSNP as of 1-18-2013.

Sequence location reference: Mus musculus strain C57BL/6J chromosome 2, GRCm38.p1 C57BL/6J, NCBI Reference Sequence: NM_010902.3.

*Error in dbSNP fixed.
